# Murine fetal bone marrow does not support functional hematopoietic stem and progenitor cells until birth

**DOI:** 10.1038/s41467-022-33092-4

**Published:** 2022-09-15

**Authors:** Trent D. Hall, Hyunjin Kim, Mahmoud Dabbah, Jacquelyn A. Myers, Jeremy Chase Crawford, Antonio Morales-Hernandez, Claire E. Caprio, Pramika Sriram, Emilia Kooienga, Marta Derecka, Esther A. Obeng, Paul G. Thomas, Shannon McKinney-Freeman

**Affiliations:** 1 Department of Hematology, Division of Experimental Hematology, St. Jude Children’s Research Hospital, 262 Danny Thomas Place, Memphis, TN 38105 USA; 2 Department of Immunology, St. Jude Children’s Research Hospital, 262 Danny Thomas Place, Memphis, TN 38105 USA; 3 Department of Oncology, St. Jude Children’s Research Hospital, 262 Danny Thomas Place, Memphis, TN 38105 USA

**Keywords:** Haematopoietic stem cells, Haematopoiesis, Stem-cell niche

## Abstract

While adult bone marrow (BM) hematopoietic stem and progenitor cells (HSPCs) and their extrinsic regulation is well studied, little is known about the composition, function, and extrinsic regulation of the first HSPCs to enter the BM during development. Here, we functionally interrogate murine BM HSPCs from E15.5 through P0. Our work reveals that fetal BM HSPCs are present by E15.5, but distinct from the HSPC pool seen in fetal liver, both phenotypically and functionally, until near birth. We also generate a transcriptional atlas of perinatal BM HSPCs and the BM niche in mice across ontogeny, revealing that fetal BM lacks HSPCs with robust intrinsic stem cell programs, as well as niche cells supportive of HSPCs. In contrast, stem cell programs are preserved in neonatal BM HSPCs, which reside in a niche expressing HSC supportive factors distinct from those seen in adults. Collectively, our results provide important insights into the factors shaping hematopoiesis during this understudied window of hematopoietic development.

## Abstract (web summary)

Relatively little is known about the first hematopoietic stem and progenitor cells to arrive in the fetal bone marrow. Here they characterize the frequency, function, and molecular identity of fetal BM HSPCs and their bone marrow niche, and show that most BM HSPCs have little hematopoietic function until birth.

## Introduction

Hematopoietic stem cells (HSCs) support lifelong hematopoiesis and are utilized regularly to treat hematologic disease^1,2^. During ontogeny, HSCs arise from the embryonic vasculature, migrate to the fetal liver (FL), and then expand before moving to bone marrow (BM)^3^. Understanding HSC regulation during ontogeny is key to dissecting the origins of pediatric hematologic disease and engineering populations from pluripotent stem cells^4^. Although much is known about HSC origins and specification, less clear is precisely when these cells, and the downstream hierarchy of hematopoietic stem and progenitor cells (HSPCs), are established in the BM.

Although the femur is vascularized by embryonic day 15 (E15)^5^ and HSC clonogenic potential is detected by E15.5^6^, the first transplantable HSCs are not detected before E16.5^7^. Also, while HSPCs—a heterogeneous pool of HSCs and lineage-biased multipotent progenitors (MPPs)—exist at consistent frequencies in adult murine BM^8,9^, there is little information regarding these populations in murine perinatal BM. Recent reports suggest that MPPs play a key role in steady-state hematopoiesis^10–12^. A better understanding of the stage-specific regulation of HSCs and MPPs during development will help clarify their roles during steady-state hematopoiesis, which may yield insight into the origins of hematopoietic malignancies—especially pediatric disease^10–13^.

Little is known about how or when the BM becomes a supportive environment for HSPCs. Fetal BM (FBM) HSCs may receive homing/maintenance cues from *Nestin*^+^ cells that display reduced expression of *Cxcl12* and other factors relative to adult BM stroma^14^. Similarly, perinatal *Cxcl12*^+^ cells have been identified, but display lower *Cxcl12* expression compared to adult *Cxcl12*^+^ cells^15^. A subpopulation of *Osterix*^+^ cells expressing *Pdgfrb* and *Foxc1*—genes essential for the development of HSC niche cells—can be found in E16.5 FBM^15^ and long-term repopulating HSCs are absent from *Osterix*^-/-^ E17.5 FBM, which also lack osteolineage cells^7^. In contrast, much work has gone into dissecting the regulatory role of the adult BM niche^16,17^. Many adult BM cells contribute to HSC maintenance, including osteolineage cells^18,19^, non-myelinating Schwann cells^20^, adipocytes^21^, macrophages^22^, megakaryocytes^23^, T-regulatory cells^24^ and neutrophils^25^. However, it is the adult BM vasculature and perivascular cells that serve as the primary niche for HSCs^16,17^. Arterioles and sinusoids both contribute to the endothelial HSC niche, with a bevy of perivascular cells contributing to HSC quiescence, BM retention and BM repopulation^26–31^. A similar exploration of the perinatal BM is needed to better understand exogenous HSC regulation during development.

Therefore, to define the hematopoietic and stromal components of murine perinatal BM, we interrogated BM HSPCs in the whole skeleton from colonization until after birth via competitive transplantation, immunophenotyping, functional interrogation of select progenitors, and single-cell RNA-sequencing (scRNA-seq) of murine BM hematopoietic and stromal cells during perinatal development. Here, we show that BM HSPCs do not acquire robust function or molecular identity until near birth, which correlates with a shift in the relative frequencies and numbers of distinct HSPCs and the develop of a supportive niche. We also identify BM niche interactions with HSPCs throughout perinatal development.

## Results

### Rare long-term repopulating activity in FBM as early as E15.5

**Fig. 1:**
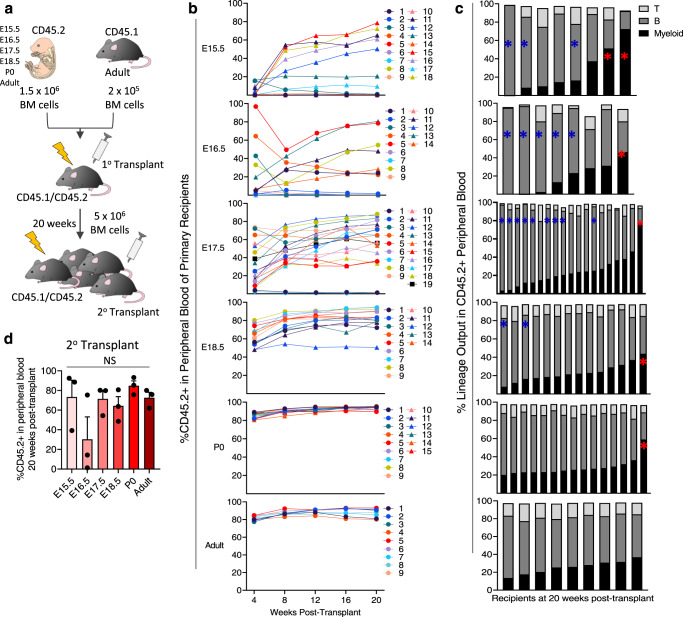
Long-term repopulating cells are detected in FBM at E15.5. **a** FBM primary and secondary transplant. **b** PB reconstitution in FBM primary recipients (*n* = 9–18). **c** Lineage output in individual primary recipients 20 weeks post-transplant (*n* = 8–19); myeloid bias (%myeloid > %B cell), red asterisk; b-lymphoid bias (%B cell >70%), blue asterisk. **d** %CD45.2+ PB in secondary recipients of FBM. Data are means ± SE of three independent transplants (*n* = 5/transplant, Mann–Whitney test, two-tailed). 1^o^, primary; 2^o^, secondary; BM bone marrow, FBM fetal BM; NS not significant. Source data are provided in the Source Data File. See also Supplementary Fig. 1.

To interrogate fetal and neonatal BM across ontogeny, BM was isolated from whole skeletons of CD45.2^+^ E15.5, E16.5, E17.5, E18.5 and postnatal day 0 (P0) embryos/neonates and transplanted at 1.5 × 10^6^ cells/mouse into irradiated CD45.1^+^/CD45.2^+^ recipients along with 2 × 10^5^ CD45.1^+^ adult BM competitor cells. Adult BM was used as a positive control (Fig. 1a). As expected, adult BM yielded long-term multi-lineage repopulation of recipients (Fig. 1b, c and Supplementary Fig. 1a). P0 BM recipients also displayed robust CD45.2^+^ contribution and balanced peripheral blood (PB) lineage output in 14/15 recipients (Fig. 1b, c).

However, PB CD45.2^+^ engraftment became heterogeneous and began to significantly decline in E18.5-E15.5 FBM recipients, many of whom also showed evidence of lineage bias (Fig. 1b, c and Supplementary Fig. 1c). For example, although 19/19 E17.5 FBM recipients displayed >1% CD45.2^+^ PB at 20 weeks post-transplant, two of these recipients only reached 1.1% and 1.3% CD45.2^+^ PB (Fig. 1b). E16.5 FBM recipients displayed reduced repopulation, as only 8/14 displayed long-term CD45.2^+^ PB > 1% (Fig. 1b). Here, variable CD45.2^+^ lineage output was seen, with 5/8 recipients engrafted with mostly B cells and 1/8 engrafted with mostly myeloid cells (Fig. 1c). Surprisingly, 8/18 E15.5 FBM recipients displayed long-term CD45.2^+^ PB (Fig. 1b). Many E15.5 FBM recipients displayed low CD45.2^+^ PB 4 weeks post transplant that gradually increased overtime (Fig. 1b), suggesting a lack of functional hematopoietic progenitors compared to later developmental timepoints, as progenitors repopulate transiently and thus contribute disproportionately to early blood production^9^. As 1.5 × 10^6^ cells is about one embryo equivalent of FBM at E15.5 (Supplementary Fig. 1b**)** and only 50% of these recipients displayed CD45.2^+^ PB > 1% long-term (Fig. 1b), E15.5 FBM long-term repopulating cells are extremely rare. Long-term repopulating cells displaying uni- or bipotential lineage output were detected from E15.5-E17.5, revealing the presence of lineage-biased or “lineage-bypassing” HSCs at these timepoints (Fig. 1c**)**^32^. However, as average myeloid and B cell lineage output between recipients of E15.5 and adult BM was not significantly different at 20 weeks post-transplant (Supplementary Fig. 1d, e), and similar trends for myeloid, B, and T cell reconstitution post-transplant were seen in recipients of E15.5 through adult BM (Supplementary Fig. 1f), there is no clear enrichment for lineage-biased HSCs at any specific developmental timepoint.

Secondary transplants confirmed the presence of self-renewing FBM HSCs as early as E15.5, as all secondary recipient cohorts of E15.5 FBM displayed long-term CD45.2^+^ PB and BM (Fig. 1d and Supplementary Fig. 1g). Interestingly, one secondary transplant cohort was reconstituted with 81% donor B cells and <1% myeloid cells (Supplementary Fig. 1h), further suggesting the presence of self-renewing lineage-biased HSCs in E15.5 FBM. In sum, we provide evidence for the presence of long-term, self-renewing HSCs in BM by E15.5 (Fig. 1d and Supplementary Fig. 1h).

### The frequency of FBM HSPCs is distinct from FL and adult BM

**Fig. 2:**
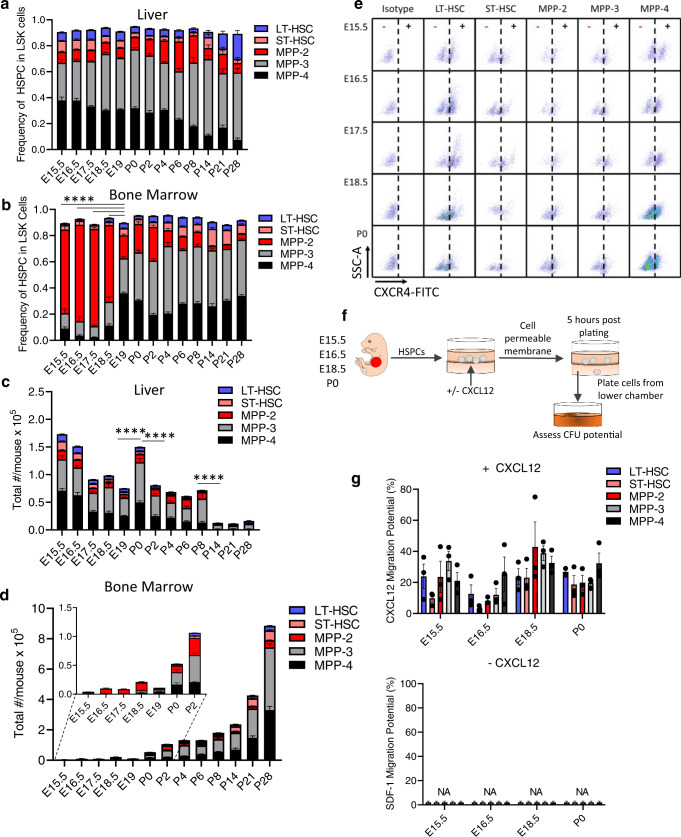
FBM HSPC frequency is distinct from FL and adult BM. Frequency in LSK cells (**a**, **b**) and absolute number (**c**, **d**) of HSPCs in murine liver (**a**, **c**) and BM (**b**, **d**). Data are means ± SE of 10–17 embryos from 2–3 litters. **** in **b**: *P* < 0.0001 comparing MPP2 frequencies; **** in **c**: *P* < 0.0001 comparing total HSPC numbers. **e** Representative flow cytometry of cell-surface CXCR4 on E15.5-P0 FL HSPCs (*n* = 2–3). **f** CXCL12 trans-well migration assay. **g** Migration of fetal and newborn liver HPSCs towards CXCL12. Data are mean ± SE of three distinct experiments. *P* values determined by Mann–Whitney test, two-tailed. CFU colony-forming unit, HSC hematopoietic stem cell, HSPC hematopoietic stem and progenitors, LSK Lineage^-^Sca1^+^c-Kit^+^, LT-HSC long^-^term HSC, MPP multipotent progenitor, ST-HSC short-term HSC. Source data are provided in the Source Data File. See also Supplementary Fig. 2.

We next examined immunophenotypic BM and FL HSPCs from E15.5 through adulthood. Here, liver and BM isolated from whole skeletons were assessed for the frequency of long-term HSCs (LT-HSCs), short-term HSCs (ST-HSCs), MPP2s, MPP3s and MPP4s^9^ (Fig. 2a, b and Supplementary Fig. 2a, b). HSPCs in the early liver were relatively constant overtime, with about equal numbers of MPP3s and MPP4s until P6 when a slight shift towards MPP3s emerged (Fig. 2a and Supplementary Fig. 2a). In contrast, MPP2s were dominant in E15.5–E18.5 FBM (Fig. 2b and Supplementary Fig. 2b). FBM LT-HSCs were ~0.01 of Lineage^-^Sca-1^+^c-Kit^+^ (LSK) cells between E15.5-E17.5 and then shifted to 0.03 ± 0.00 at E18.5, similar to P28 BM (Fig. 2b, Supplementary Fig. 2c).

We also assessed absolute numbers of HSPCs. As HSPCs migrate from FL to BM from E15.5 until after birth^3,33^, we expected decreasing numbers of FL HSPCs to coincide with an increase in FBM HSPCs starting at E15.5. We observed a steady decline in total FL HSPCs from E15.5 until P0. Surprisingly, we observed a significant burst in FL HSPCs at P0 (Fig. 2c). This was followed by a drop in FL HSPCs at P2, which remained constant until P8 (Fig. 2c**)**. FL HSPC numbers significantly declined from P8 to P14, from ~60,000 to ~11,000 per mouse (Fig. 2c). Thus, aside from a surprising jump in FL HSPCs at P0, FL HSPCs decline from E15.5 to P8 and are nearly exhausted by P14. BM HSPCs displayed the opposite trend. While P28 BM contained ~960,000 HSPCs/mouse (84% MPP3s or MPP4s), E15.5 FBM contained only ~4,800 HSPCs/mouse. FBM HSPCs increased from E15.5 to P28 with a small drop in numbers at E19 (Fig. 2d). LT-HSCs significantly increased from only ~60 at E15.5 to ~4,800 by P2 (Fig. 2d and Supplementary Fig. 2d). As FL HSPCs expressed CXCR4 and responded to CXCL12 in migration assays (Fig. 2e-g)^34^, FBM HSPCs likely represent both itinerant HSPCs and HSPCs arising from recently engrafted progenitors. Additionally, a larger fraction of each FBM HSPC population examined expressed CXCR4 relative to FL HSPCs at P0 except for LT-HSCs, which could result from internalization of CXCR4 in LT-HSCs after migration to the FBM (Supplementary Fig. 2e). P0 BM HSPCs also displayed significantly increased migration toward CXCL12 compared to FL HSPCs (Supplementary Fig. 2f). In sum, the composition of FL and FBM HSPCs are distinct during early ontogeny and FBM HSPCs shift around birth from an MPP2-dominant to an MPP3/MPP4-dominant compartment (Fig. 2a–d).

### FBM HSPCs are functionally distinct from FL and adult HSPCs

**Fig. 3:**
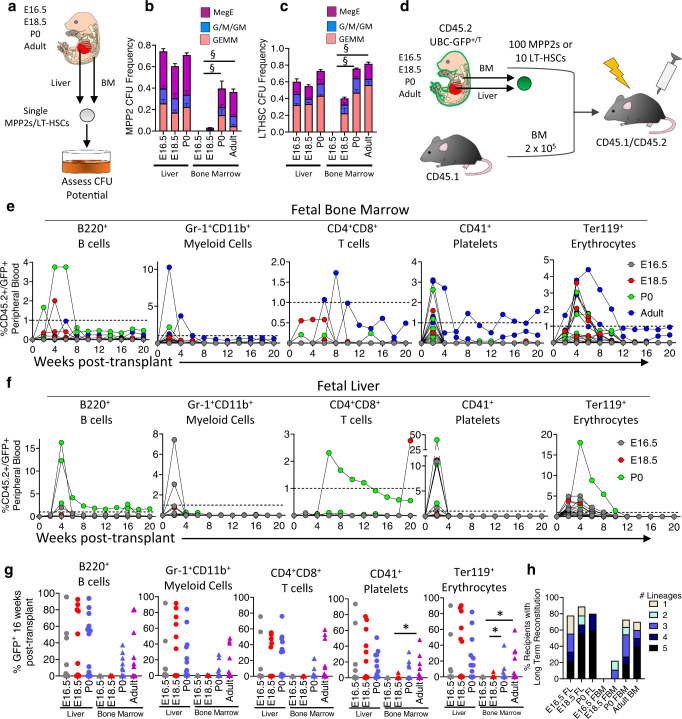
FBM MPP2s are functionally distinct from FL and adult BM MPP2s. **a** CFU assay of single MPP2 and LT-HSC cells. **b** BM and liver MPP2 CFUs frequency. Data are means ± SE of 87–171 single cells from three distinct experiments. **c** Frequency of CFUs among LT-HSCs from BM and liver. Data are means ± SE of 111–175 single cells from three separate experiments. **d** MPP2 and LT-HSC transplant. **e** %CD45.2/GFP PB lineages in BM MPP2 transplant recipients (*n* = 9–10 recipients/timepoint). **f** %CD45.2/GFP PB lineages in liver MPP2 recipients (*n* = 5–9 recipients/timepoint). **g** %CD45.2/GFP PB lineages in LT-HSC recipients at 16 weeks post-transplant (*n* = 9–14 recipients/timepoint). Platelets: *P = 0.0102; Erythrocytes: * top, *P* = 0.0162; * bottom, *P* = 0.0440. **h** %Recipients with long-term PB donor reconstitution in (**g**) (*n* = 9-14 recipients). ^§^*P* = 0.1; *p < 0.05. *P* values determined by Mann–Whitney test, two-tailed. BM bone marrow, CFU colony-forming unit, FBM fetal BM, FL fetal liver, G granulocyte, GEMM granulocyte/erythroid/ monocyte/megakaryocyte, HSC hematopoietic stem cell, LT-HSC long-term HSC, M monocyte, MegE megakaryocyte/erythroid, MPP multipotent progenitor. Source data are provided in the Source Data File. See also Supplementary Fig. 3.

The paucity of CD45^+^ cells in the FBM between E15.5 and E17.5, relative to later timepoints, suggests that FBM HSPCs may contribute little to blood production in the early FBM (Supplementary Fig. 3a, b). To explore this, we tested the function of FBM HSPCs, beginning with MPP2s, as they are the most abundant HSPC population in the FBM and display characteristic activity when interrogated functionally (i.e. a bias for megakaryocyte/erythroid output in CFU assays and when transplanted)^9^. Single MPP2s from E16.5, E18.5, P0 and adult BM and liver were sorted into methylcellulose supplemented with cytokines to assess CFU potential (Fig. 3a). E16.5 FBM MPP2s yielded no CFUs, while E18.5 FBM MPP2s displayed a small amount of activity (0.03% of cells) (Fig. 3b**)**. In contrast, P0 and adult BM MPP2s displayed robust CFU potential with the expected lineage output (Fig. 3b). Importantly, liver MPP2s displayed high CFU potential at all timepoints (Fig. 3b). Thus, although FBM MPP2s are immunophenotypically identical to FL MPP2s and presumably seeded from FL (Fig. 2g), their in vitro CFU potential is distinct, and the first robustly functional BM MPP2s appear around birth (Fig. 3b).

To explore this further, E16.5, E18.5, P0 and adult CD45.2^+^ BM and liver MPP2s were transplanted into irradiated CD45.1^+^/CD45.2^+^ recipients at 100 cells/animal, with 2 × 10^5^ CD45.1^+^ adult supportive cells (Fig. 3d). Here, *UBC-GFP* mice served as donors to facilitate visualization of donor-derived erythrocytes and platelets^35^ (Supplementary Fig. 3c, d). As expected, adult BM MPP2s transiently reconstituted 50% of recipients with myeloid, platelet, and erythroid cells, with one recipient displaying T cell reconstitution (Fig. 3e and Supplementary Fig. 3e). In contrast, E16.5 FBM MPP2s did not reconstitute any recipients. E18.5 FBM MPP2s transiently reconstituted 20% of recipients’ platelets, erythroid, and B cells (Fig. 3e and Supplementary Fig. 3e), while 40% of P0 FBM MPP2 recipients displayed transient reconstitution of all lineages except T cells (Fig. 3e and Supplementary Fig. 3e). Meanwhile, E16.5, E18.5 and P0 FL MPP2s transiently reconstituted multiple lineages in 67%, 83% and 40% of recipients, respectively (Fig. 3f and Supplementary Fig. 3e). Thus, the early FBM MPP2 pool displays little function until around birth, at which point they display robust function, similarly to their liver and adult BM counterparts.

To assess if additional BM HSPCs acquire full functional potential near birth, we next interrogated perinatal LT-HSCs (Fig. 3a, d). As with perinatal MPP2s, and in sharp contrast to FL LT-HSCs, the in vitro CFU potential of single bone marrow LT-HSCs was absent until around birth (Fig. 3c). We next transplanted 10 LT-HSCs into recipients with 2 × 10^5^ supportive cells (Fig. 3d). As expected, E16.5, E18.5, and P0 liver as well as adult BM LT-HSCs displayed robust and multi-lineage long-term PB reconstitution (Fig. 3g, h). In contrast, E16.5 and E18.5 FBM LT-HSCs displayed virtually no long-term reconstitution; robust repopulation of multiple lineages was not apparent until P0 (Fig. 3g, h). Given that we identified hematopoietic repopulating cells as early as E15.5 in the FBM (Fig. 1), these data suggests that *immunophenotypic* HSPCs in the bone marrow display little to no function until near birth.

### Transcriptional signatures of stemness increase at birth in BM HSPCs

**Fig. 4:**
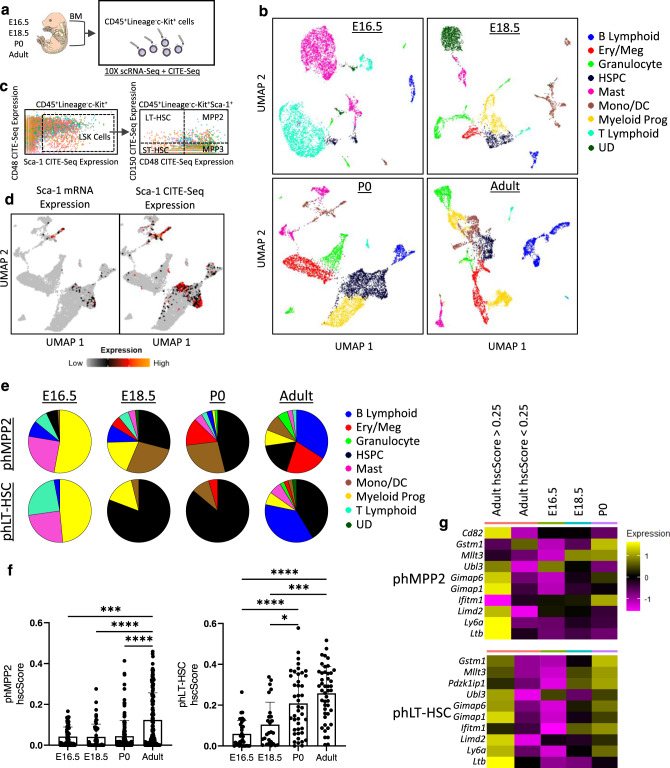
Remodeling of perinatal BM HP and HSPCs during late gestation. **a** ScRNA-seq of HPs of perinatal and adult BM. **b** UMAP projections of cell clusters of HPs from adult, P0, E18.5, and E16.5 BM. **c** CITE-Seq dot plots for identification of phHSPCs. **d** UMAP projection of P0 BM depicting Sca-1 mRNA and CITE-Seq. **e** Distribution of phMPP2s or phLT-HSCs in each cell cluster of adult, P0, E18.5, or E16.5 HPs. **f** hscScore among phMPP2s (left, *n* = 55–147 single cells; ***P = 0.0008; ****P < 0.0001) or phLT-HSCs (right, *n* = 26–46 single cells; *P = 0.0254; ***P = 0.0001; ****P < 0.0001) across development. Data are presented as means ± SD. **g** Expression level of hscScore-related genes within phMPP2s and phLT-HSCs across development. *P* values determined by Mann–Whitney test, two-tailed. BM bone marrow, CITE-Seq cellular indexing of transcriptomes and epitopes by sequencing, Ery/Meg erythroid/megakaryocyte, HP hematopoietic progenitors, HSPCs hematopoietic stem and progenitors, LT-HSC long-term hematopoietic stem cell, Mono/DC monocyte/dendritic cells, MPP multipotent progenitor, ph immunophenotypic, Prog progenitors, scRNA-seq single-cell RNA-sequencing, UD undefined. Source data are provided in the Source Data File. See also Supplementary Fig. 4.

To explore why FBM HSPCs display little function relative to FL and adult BM HSPCs, we performed 10X scRNA-Seq on CD45^+^Lineage^−^c-Kit^+^ hematopoietic progenitors (HPs) from E16.5, E18.5, P0, and adult BM (Fig. 4a). As FBM HSPCs are rare, we used Cellular Indexing of Transcriptomes and Epitopes by Sequencing (CITE-Seq) to label HSPCs with oligo-tagged antibodies that could later be combined with mRNA expression to identify immunophenotypic (ph) HSPCs in our scRNA-Seq dataset^36^ (Fig. 4a and Supplementary Fig. 4a–d). In total, we constructed and sequenced libraries from 5985 adult BM, 6208 P0 BM, 3477 E18.5 FBM, and 5312 E16.5 FBM HP cells. Unbiased clustering based on variable gene expression identified multiple distinct clusters in each HP library. We annotated each cluster based on the expression of cell type-specific markers from public datasets^37–51^ (Fig. 4b, Supplementary Fig. 4e–v, and Supplementary Data 1), and Uniform Manifold Approximation and Projection (UMAP) was used for visualization of transcriptional variation (Fig. 4b, Supplementary Fig. 4g, l, q, v).

E18.5, P0, and adult BM HP libraries contained similarly annotated populations, including HSPCs, granulocyte, monocyte, mast cell, megakaryocyte/erythroid progenitor, and B cell clusters (Fig. 4b and Supplementary Fig. 4e-v). P0 BM contained more HSPCs than adult and E18.5 BM, confirming expansion around birth (Fig. 4b and Supplementary Fig. 4e–v and Fig. 2a–d). E16.5 HPs were striking for the presence of multiple large mast and T cell clusters (Fig. 4b and Supplementary Fig. 4e–v). E16.5 FBM also lacked defined myeloid, erythroid, megakaryocyte, or lymphoid progenitors, but did contain a small cluster of HSPCs (Fig. 4b, Supplementary Fig. 4e-v). CITE-Seq antibodies identified phMPP2s and phLT-HSC^36^ (Fig. 4c); antibody abundances correlated with gene expression (Fig. 4d and Supplementary Fig. 4a–d). phMPP2s failed to segregate into a single hematopoietic lineage and displayed heterogeneity across ontogeny, with phMPP2s falling into clusters annotated as myeloid progenitors with mono/mast signatures (53%, the “Mono/Mast” cluster in Supplementary Fig. 4f, g) at E16.5, HSPCs (29%) at E18.5, HSPCs (46%) at E18.5, and B-lymphoid cells (34%) in the adult (Fig. 4e), consistent with reports that MPP2s are functionally heterogeneous^8,9^. Most phLT-HSCs segregated with HSPCs at all timepoints except E16.5 (Fig. 4e). Interestingly, many phLT-HSCs were found in several T-cell clusters at E16.5 (Fig. 4e).

We next applied hscScore to interrogate changes in the stem cell signature of phMPP2s and phLT-HSCs across development^52^. hscScore assigns a score to each cell based on the expression of genes enriched in functional LT-HSCs, with higher scores correlating with increased functional potential of HSPCs^50^. Integration of hscScore into our dataset revealed a clear trend toward increasing hscScores in phMPP2s across development, and a significant increase in hscScores among phLT-HSCs around birth (Fig. 4f), consistent with our functional studies (Fig. 3). For instance, while most phMPP2s displayed hscScores <0.1 across development, by E18.5/P0 there was an increase in the number of MPP2s with hscScores >0.15, and adult phMPP2s contained many cells with hscScores ranging from 0.15-0.45 (Fig. 4f). Meanwhile, most phLT-HSCs displayed hscScores <0.2 at E16.5, suggesting that many of these cells lack an HSC gene signature, but by P0 the average phLT-HSC hscScore was >0.2 (Fig. 4f). As expected, phLT-HSC hscScores were higher across development than phMPP2, and high hscScores in phLT-HSCs at later timepoints were mostly driven by HSPCs (Supplemental Fig. 4n, s, x). Finally, by analyzing the average gene expression of the top hscScore-related genes in adult phMPP2 and phLT-HSCs, we determined that increasing hscScores in phMPP2s and phLT-HSCs during development is driven by similar genes, including *Ifitm1*, *Mllt3*, and *Ly6a* (Fig. 4g). Thus, FBM MPP2s and LT-HSCs function (Fig. 3) correlates with the acquisition of HSC transcriptional programs, which are absent in phMPP2 and phLT-HSC prior to E18.5.

### Early FBM HSPCs are transcriptionally distinct from FL HSPC

**Fig. 5:**
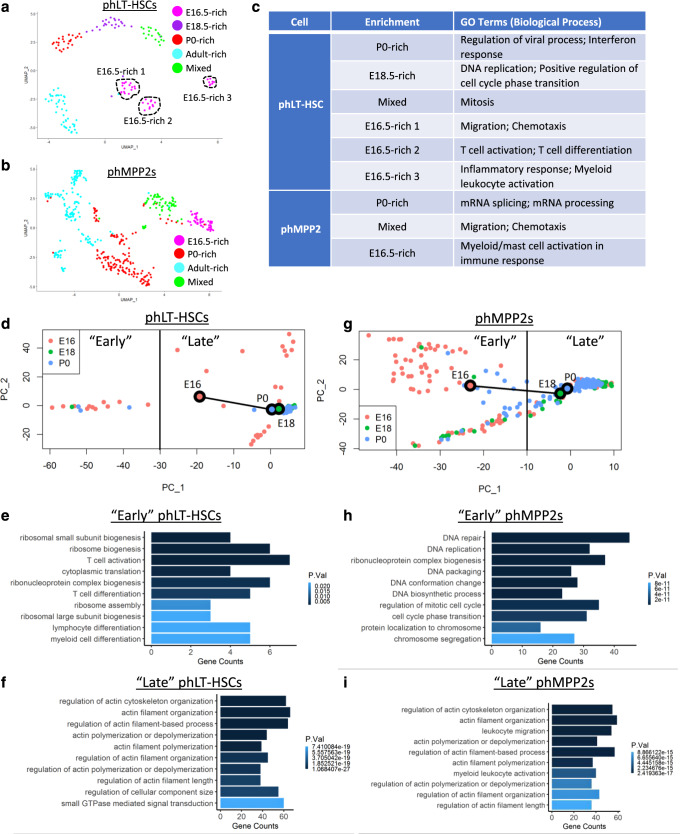
Early FBM HSPCs lack functional HSPC transcriptional programs. Integration of phLT-HSCs (**a**) and phMPP2s (**b**) from all developmental timepoints. **c** Primary GO Terms for phLT-HSCs and phMPP2s. **d** Trajectory analysis and GO terms (**e**, **f**) of perinatal phLT-HSCs. **g** Trajectory anlaysis and GO terms (**h**, **i**) of perinatal phMPP2s. “Early” and “Late” cells were determined by dissecting PC1 according to clear separation of cells by developmental time. P-values determined by Bonferroni correction. BM bone marrow, FBM fetal BM, GO gene ontology, HSPC hematopoietic stem and progenitors, LT-HSC long-term hematopoietic stem cells, MPP multipotent progenitor, ph immunophenotypic. See also Supplementary Fig. 5.

We next combined and re-clustered all phLT-HSCs and phMPP2s across development (Fig. 5a, b and Supplementary Fig. 5a-b). phLT-HSCs clustered along developmental time, segregating into adult-rich, P0-rich, E18.5-rich, and E16.5-rich phLT-HSC clusters, although one cluster was a mix of all perinatal phLT-HSCs (Fig. 5a and Supplementary Fig. 5a). phMPP2s also clustered along developmental time, although there was no clear E18.5-rich cluster (Fig. 5b and Supplementary Fig. 5b). Cluster-specific genes were subjected to Gene Ontology (GO) analysis (Fig. 5c and Supplementary Fig. 5c). E18.5-rich and mixed phLT-HSCs were enriched for cell cycle and mitosis, while P0-rich phLT-HSCs were enriched for regulation of viral process and interferon response (Fig. 5c, Supplementary Fig. 5c, and Supplementary Data 2), consistent with previous studies that LT-HSCs are rapidly cycling around birth^53^ and that increased type I interferon signaling drives a transition from fetal to adult HSPC transcriptional states around birth^54^. The three E16.5-enriched phLT-HSCs clusters displayed unique signatures, including migration/chemotaxis, T-cell activation/differentiation, and inflammatory response/myeloid leukocyte activation (Fig. 5c and Supplementary Fig. 5c). E16.5-like and mixed phMPP2s showed enrichment of inflammation and migration/chemotaxis, respectively, while mRNA splicing/processing was enriched in P0 phMPP2s (Fig. 5c and Supplementary Fig. 5c).

We next performed trajectory analyses on pooled phLT-HSCs and phMPP2s from E16.5, E18.5, and P0 (Fig. 5d–i). We segregated our trajectory plots along developmental time and performed GO analysis on “Early” and “Late” phLT-HSCs or phMPP2s (Fig. 5d–i), which were enriched for E16.5 or P0 cells, respectively. “Early” phLT-HSCs displayed strong enrichment for T cell activation/differentiation (Fig. 5e). It is important to note, however, that this enrichment for T cell activation/differentiation may be driven by a subset of E16.5 phLT-HSCs (Fig. 5c), or may represent a migratory population of T cells with an immunophenotype similar to LT-HSCs. Meanwhile, “Late” phLT-HSCs showed enrichment for actin polymerization and organization (Fig. 5f), which may reflect enhanced engraftment of late-stage perinatal HSCs, as genes involved in cytoskeletal organization are necessary for HSC engraftment^55,56^. “Late” phMPP2s were also enriched for actin polymerization and organization, while “early” phMPP2s showed enrichment for mitosis and DNA replication (Fig. 5h–i).

**Fig. 6:**
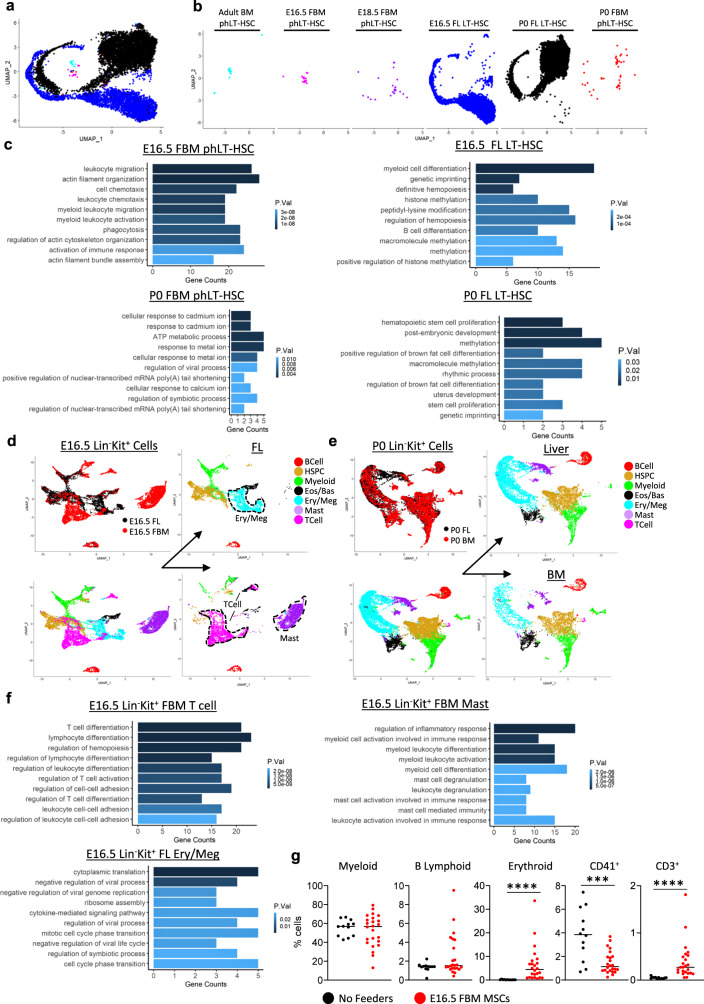
FL and FBM HP and HSPCs are transcriptionally distinct. **a**, **b** Merge of FBM phLT-HSCs across development with E16.5 and P0 FL LT-HSCs. **c** GO terms for FBM phLT-HSCs and FL LT-HSCs at E16.5 and P0. **d** Merge of E16.5 Lin^-^Kit^+^ cells from FBM and FL. **e** Merge of P0 Lin^-^Kit^+^ cells from BM and liver. **f** GO terms for E16.5 FBM T cells (left), E16.5 FBM Mast cells (middle), and E16.5 FL Ery/Meg cells (right). **g** Differentiated cell output of E16.5 FL LT-HSCs cultured with or without E16.5 BM MSCs. ***P = 0.0008; ****P < 0.0001. **c**, **f** *P* values determined by Bonferroni correction. **g** *P* values determined by Mann–Whitney test, two-tailed. BM bone marrow, Eos/Bas eosinophil/basophil, Ery/Meg erythroid/megakaryocyte, FBM fetal BM, FL fetal liver, GO gene ontology, HP hematopoietic progenitors, HSPC hematopoietic stem and progenitors, LT-HSC long-term hematopoietic stem cell, MPP multipotent progenitor, MSCs mesenchymal stroma cells, ph immunophenotypic. Source data are provided in the Source Data File. See also Supplementary Fig 5e.

To assess if transcriptional changes across development were intrinsically driven, we compared FBM phLT-HSCs and HPs with similar phenotypic FL populations. We integrated our FBM data with recently published FL scRNA-Seq data^54^ (Fig. 6a). Interestingly, while E18.5 and P0 FBM phLT-HSCs showed overlap with FL LT-HSCs, E16.5 FBM phLT-HSCs clustered separately, suggesting distinct transcriptional programs governing FBM and FL LT-HSCs at E16.5 (Fig. 6a, b). Indeed, E16.5 FBM phLT-HSCs were enriched for migration and myeloid leukocyte activation, while E16.5 FL LT-HSCs showed high enrichment for myeloid cell differentiation and definitive hematopoiesis (Fig. 6c). Meanwhile, P0 FBM phLT-HSCs were enriched for cadmium/metal ion response, primarily driven by expression of the AP-1 complex genes *Fos* and *Jun*, and the matrix metalloproteinase *Mt1* (Fig. 6c and Supplementary Data 2), while P0 FL LT-HSCs showed enrichment for HSC proliferation (Fig. 6c). The disparity in GO terms among developmentally similar but locally distinct HSC populations suggests a local effect on HSC transcriptional programs.

We next merged our E16.5 and P0 FBM Lin^-^Kit^+^ scRNA-Seq data with analogous FL cells^54^ (Fig. 6d, e). While P0 FBM and FL Lin^-^Kit^+^ HPs integrated well (Fig. 6e), the composition of E16.5 FBM and FL HPs was starkly different, including the expanded T cell and mast cell subsets in the FBM and an expanded Ery/Meg subset in the FL (Fig. 6d). GO analysis revealed that the FBM T cell and FL Ery/Meg populations are likely expanding while FBM mast cells appear inflammatory (Fig. 6f). To test whether the disparity in cell lineages within the E16.5 FL and FBM were due to intrinsic or niche-driven effects, we cultured E16.5 FL HSCs in differentiation media with or without E16.5 FBM mesenchymal stroma cells (MSC) (Fig. 6g). Strikingly, E16.5 FL HSCs cultured on E16.5 MSCs displayed a significant increase in CD3^+^ and erythroid cell production and a significant decrease in megakaryocytic differentiation (Fig. 6g). The increase in differentiation toward CD3^+^ cells was consistent with the lineage disparities between E16.5 FBM and FL cells, and the decrease in megakaryocytic differentiation may in part reflect the lack of ery/meg lineage cells in E16.5 FBM (Fig. 6d). To further assess if this effect was driven by E16.5 FBM, we differentiated E16.5 FL HSCs in the presence of E16.5 or P0 BM MSCs. E16.5 FBM MSCs drove FL HSCs towards significantly more CD3^+^ differentiation and significantly less megakaryocyte differentiation than P0 BM MSCs (Supplementary Fig. 5e), demonstrating that E16.5 FBM likely contributes to the disparities in hematopoietic lineages between the FBM and FL.

In sum, these data demonstrate that LT-HSCs and MPP2s are under inflammatory stress in the E16.5 FBM, which likely contributes to their lack of function in CFU and transplantation assays (Fig. 3 and Fig. 4g). Meanwhile, a subset of LT-HSCs may also give rise to the unexpected T-cell subpopulation of Lin^−^Kit^+^ cells in the E16.5 FBM through extrinsic regulation—a mechanism that may be absent from E16.5 FL (Fig. 6d–g, Supplementary Fig. 5e).

### FBM stroma lacks CXCL12-abundant reticular cells

**Fig. 7:**
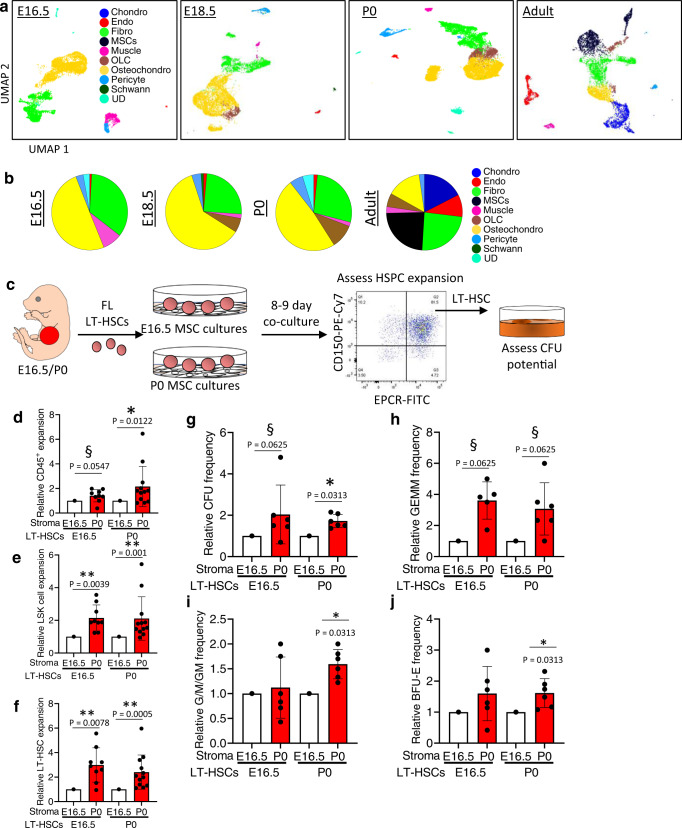
Postnatal bone marrow displays enhanced capacity as an LT-HSC supportive niche compared to fetal bone marrow. **a** UMAP projections of E16.5, E18.5, P0, and adult BM stroma. **b** Distribution of stroma amongst E16.5, E18.5, P0, or adult libraries. **c** Co-culture of fetal liver LT-HSCs on E16.5 and P0 BM MSC cultures to determine the effects on HSPC expansion and CFU potential. Relative expansion of **d** CD45^+^, **e** LSK, and **f** LT-HSC cells on P0 BM stroma compared to E16.5 BM stroma. Data represent mean and standard deviation (*n* = 9–12). Relative frequency of (**g**) total CFU, (**h**) GEMM, (**i**) G/M/GM, and **j** BFU-E colonies from FL LT-HSCs after co-culture with P0 BM stroma compared to E16.5 BM stroma. Data represent mean and standard deviation (*n* = 6). ^§^*P* < 0.1; *P < 0.05; **P < 0.01. *P* values were determined via Wilcoxon Signed Rank Test, two-tailed. BFU-E burst-forming unit-erythroid, BM bone marrow, CFU colony-forming unit, Chondro chondrocytes, Endo, endothelial cells, Fibro fibroblasts, FL fetal liver, G granulocyte, GEMM granulocyte/erythroid/monocyte/megakaryocyte, HSPC hematopoietic stem and progenitors; LSK Lineage^-^Sca1^+^c-Kit^+^, LT-HSC long-term hematopoietic stem cells, M monocyte, MSCs mesenchymal stem cells, OLC osteolineage cells, Osteochondro osteochondro progenitors, UD undefined cluster. Source data are provided in the Source Data File. See also Supplementary Fig. 6 and Supplementary Fig. 7.

To explore the role of the niche in fetal and perinatal BM HSPC biology, we isolated CD45^−^Ter119^−^ cells from E16.5, E18.5, P0, and adult BM, followed by 10X scRNA-Seq (Fig. 7a and Supplementary Fig. 5d). In total, we constructed and sequenced libraries from 7380 adult BM, 16,412 P0 BM, 7272 E18.5 FBM, and 9,786 E16.5 FBM stromal cells. Unbiased clustering identified multiple cell clusters in each library, which were again annotated using cell type-specific markers gleaned from public datasets^38,57–62^ (Fig. 7a, Supplementary Fig. 6a–l). Across development, we detected many osteo, chondrocyte, fibroblast, pericyte, and endothelial populations (Fig. 7a, Supplementary Fig. 6a-l, and Supplementary Data 1). However, there were key differences between perinatal and adult stroma. For instance, most perinatal stromal cells displayed transcriptional signatures indicative of osteochondro progenitors, while osteolineage cells first appeared at E18.5-P0, confirming transition from a predominately cartilaginous environment around birth^63–65^ (Fig. 7a, Supplementary Fig. 6a–l). Also, sinusoidal (SECs) and arterial endothelial cells (AECs) could not be distinguished in E16.5-P0 BM, although this does not preclude their existence and may be due to the small number of endothelial cells profiled in fetal/neonatal BM (Fig. 7a, Supplementary Fig. 6a–l). Meanwhile, adult stroma contained clear MSC populations, including Pdgfra^+^Sca-1^+^ (PαS) MSCs and CXCL12-adundant reticular (CAR) MSCs (Fig. 7a, Supplementary Fig. 6a–l)—both of which were absent from E16.5-P0 BM. CAR cells regulate adult BM HSC and are defined by high expression of *Cxcl12* and *Kitl*, as well as other secreted factors, such as *Angpt1*^63–65^. Many of the CAR associated factors in the perinatal BM were expressed within several fibroblast subsets (Supplementary Fig. 6a–i). To clearly identify a CAR-like population in the perinatal BM,

we constructed an expression signature for CAR cells based on the adult CAR marker genes and looked for cells with a similar expression pattern in perinatal BM (Supplementary Fig. 6m). A clear CAR-like population was not evident at any perinatal timepoint (Supplementary Fig. 6m), suggesting that this specific cell type is absent from the perinatal BM space and only appears later in development. Thus, perinatal BM changes composition around birth and lacks the correlate populations that regulate adult BM HSPCs.

### Neonatal BM can function as a LT-HSC supportive niche

Given the compositional changes in BM stroma (Fig. 7a, b, Supplementary Fig. 6) and sudden burst of functional BM HSPCs around birth (Fig. 3), we hypothesized that P0 BM constitutes a more favorable niche for HSPCs than early FBM. To test this, we established multiple independent mesenchymal stromal cell (MSC) cultures from E16.5 and P0 BM (Supplementary Fig. 7a–c). We then interrogated the ability of these cultures to support LT-HSCs isolated from E16.5 or P0 FL (Fig. 7c). Here, 1000 LT-HSCs were plated on established MSC cultures. Significantly greater expansion of CD45^+^ cells, LSK cells and phenotypic LT-HSCs was observed in P0 MSC cultures relative to E16.5 MSC cultures (Fig. 7d–f). Indeed, E16.5 FL LT-HSCs expanded nearly 3-fold more on P0 BM MSCs than on E16.5 BM MSCs (Fig. 7f). Further, E16.5 and P0 LT-HSCs co-cultured with P0 MSCs displayed greater CFU potential, especially CFU-GEMM potential, compared to LT-HSCs co-cultured with E16.5 MSCs (Fig. 7g–j). In total, these data support our hypothesis that neonatal BM constitutes a more favorable niche for HSPCs than early FBM.

### RNA-Magnet reveals niche cells for perinatal HSPCs

**Fig. 8:**
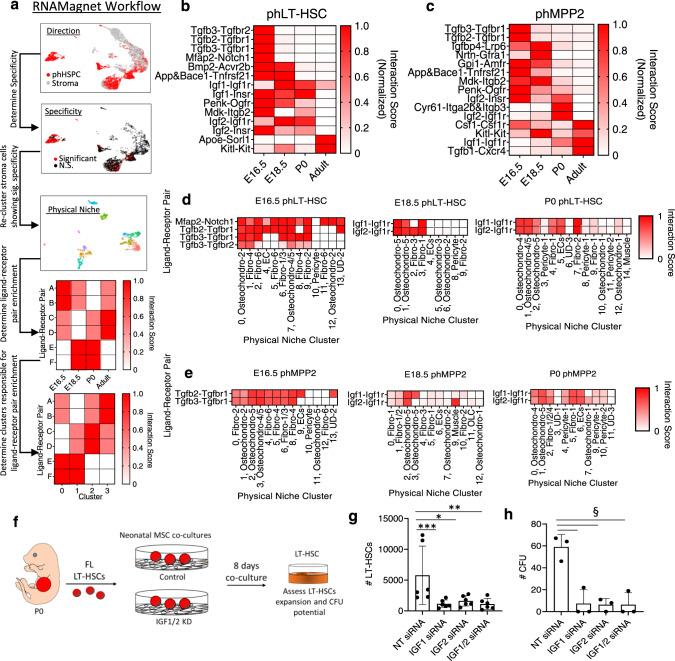
Physical niches and niche factors for perinatal BM HSPCs. **a** RNA-Magnet workflow. **b** Normalized interaction scores of ligand-receptor pairs with significant interaction (secreted ligand from stroma, receptor from phLT-HSCs) **c** Normalized interaction scores of ligand-receptor pairs with significant interaction (secreted ligand from stroma, receptor from phMPP2s). Interaction scores for each significant ligand-receptor pair (rows) across development within clusters of the phLT-HSC (**d**) or phMPP2 (**e**) physical niche (columns, with dominant stromal cell type indicated for each physical niche cluster). **f** Schematic of P0 FL HSCs co-cultures with IGF1 or IGF2 siRNA-treated P0 MSCs. **g** # phLT-HSCs after 8 days of FL LT-HSCs co-culture on control, IGF1, IGF2 or IGF1/2 siRNA-treated P0 MSCs (*n* = 6; *P = 0.0260; **P = 0.0087; ***P = 0.0022). **h** Total # CFUs via phLT-HSCs isolated from co-cultures in (**g**) (*n* = 3, ^§^P = 0.1). Data are presented as means ± SD. *P* values determined by Mann–Whitney Test, two-tailed. BM, bone marrow, CFU colony-forming unit, EC endothelial cells, Fibro fibroblast, FL fetal liver, HSPC hematopoietic stem and progenitors, KD knockdown, LT-HSC long-term hematopoietic stem cells, MPP multipotent progenitors, MSC mesenchymal stroma cells, N.S, not significant, NT non-targeting control, OLC osteolineage cell, Osteochondro osteochondroprogenitor, ph immunophenotypic, Sig. significant, UD undefined cluster. Source data are provided in the Source Data File. See also Supplementary Fig. 8.

To gain further insight into the diminished supportive potential of E16.5 BM stroma, we next sought to identify a physical niche for perinatal HSPCs using RNA-Magnet^38^, an algorithm that predicts physical interactions between BM cells using the expression patterns of cell-surface receptors and their binding partners^38^. For this, we combined scRNA-Seq data for HSPCs with their corresponding stroma (e.g. E16.5 phLT-HSCs with E16.5 stroma) and used RNA-Magnet to identify stroma cells showing significant specificity toward HSPCs (Fig. 8a and Supplementary Fig. 8a–j).

The predicted physical niches for adult phLT-HSCs and phMPP2s were similar and composed of arterial endothelial cells, sinusoidal endothelial cells, MSC-CARs, MSCs-PαS cells, and an unexpected fibroblast population (Fibro-2) (Supplementary Fig. 8e, j). As endothelium and CARs are known to regulate adult BM HSPCs, this validates the fidelity of RNA-Magnet;^16,17^ however, only a subset of CARs showed significant specificity toward phLT-HSCs (Supplementary Fig. 8k). Fibro-2 was mostly differentiated from other fibroblast populations by expression of *Ctsk*, *Acta2*, and *Tagln*, suggesting a myofibroblast-like signature (Supplementary Fig. 6k). Interestingly, adult HPSCs may occupy distinct physical niches, as phST-HSCs, phMPP2s, phMPP3s, and phMPP4s each showed unique direction and specificity toward adult stroma (Supplementary Fig. 8l), consistent with recent reports^66^.

E16.5 phLT-HSC and phMPP2 niches were diverse at the cellular level, suggesting the absence of a highly defined physical niche at this timepoint (Supplementary Fig. 8e, j). Interestingly, an osteochondro progenitor (Osteochondro-5) population emerged at E18.5 as a primary component of the phLT-HSC and phMPP2 niches (Supplementary Fig. 8e, j). Osteochondro-5 displayed higher expression of *Acan, Sox9, Col2a1*, and *Sparc* compared to other osteochondro progenitors, but did not show high expression of many of the traditional factors associated with HSC maintenance (Supplementary Fig. 6e). Two similar populations (Osteochondro-4 and Osteochondro-5) were present at P0 (Supplementary Fig. 6e), constituting the majority of the P0 phLT-HSC and phMPP2 niche (Supplementary Fig. 8e, j). Endothelial cells were consistently part of the fetal and perinatal niches for phLT-HSCs and phMPP2s (Supplementary Fig. 8e, j). While our co-cultures do not contain endothelial cells, it was recently shown that AECs are a source of WNT2, which may support HSPC expansion in the perinatal femur^67^. Thus, endothelial cells and osteochondro progenitors may be important for preserving BM HSPC function around birth.

RNA-Magnet can also define putative signaling interactions between cell types by prioritizing the expression of secreted ligands and their receptors^38^. Therefore, we next sought to identify the primary stroma-derived secreted factors in our defined perinatal BM physical niches acting on HSPCs (Fig. 8a–e). Predicted ligand-receptor pairs implicated TGF-β as the primary secreted ligand interacting with E16.5 phHSPCs (Fig. 8b–e); nearly all stroma subclusters showed high interaction scores for TGF-β ligand-receptor pairs (Fig. 8d, e), suggesting a physical niche rich in signals that inhibit HSPC expansion^68^. Interestingly, the primary ligand-receptor pairs with significant interaction at E18.5 and P0 phLT-HSCs/phMPP2s were IGF1-IGF1R and IGF2-IGF1R (Fig. 8b, c). Importantly, these interactions were low or absent at E16.5. The main components of the E18.5 and P0 phLT-HSC/phMPP2 niches—Osteochondro-4/5—were found in subclusters with high interaction scores for these ligand-receptor pairs (Fig. 8d, e), along with some fibroblast clusters at E18.5 and P0. Although rare, endothelial cells also contributed to the high interaction scores for IGF1/2-IGF1R at P0 but not at E18.5 (Fig. 8d, e). CXCL12-CXCR4 was not identified as having a significant interaction score for any timepoint but did display interesting dynamics as a ligand-receptor pair.

IGF1 is a critical regulator of LT-HSC function and hematopoietic health, as the early hallmarks of LT-HSC aging can be rejuvenated with IGF1^69^. Therefore, we tested if IGF1 and IGF2 are required to support neonatal HSPCs in perinatal MSC co-cultures. We first interrogated levels of IGF1 and IGF2 in the supernatant of E16.5 and P0 BM MSCs cultures and found IGF1 significantly increased in P0 MSC cultures relative to E16.5 MSC cultures (Supplementary Fig. 8m). IGF2 levels were also higher in P0 cultures relative to E16.5 cultures, but this was not statistically significant (Supplementary Fig. 8n). Next, we co-cultured P0 FL LT-HSCs with P0 BM MSCs treated with anti-IGF1, IGF2, or control siRNAs (Fig. 8f). We confirmed efficient and lasting knockdown of IGF1 and IGF2 in siRNA-treated BM MSCs (Supplementary Fig. 8o, p). P0 FL LT-HSCs cultured with IGF1 or IGF2 deficient P0 MSCs produced fewer phenotypic LT-HSCs than those cultured with control P0 MSCs (Fig. 8g), and phenotypic LT-HSCs isolated from IGF-deficient P0 MSC co-cultures yielded far fewer CFUs than those isolated from control cultures (Fig. 8h). These data support a model in which IGF1 and IGF2 play a functional role in the perinatal LT-HSC BM niche.

In sum, the lack of a defined physical niche at E16.5 likely contributes to the compromised function of E16.5 BM HSPCs. This is supported by the diminished function of E16.5 MSC cultures compared to P0 MSC cultures (Fig. 7). The presence of signals antithetical to LT-HSC and MPP2 proliferation (*e.g*. TGF-β) may also contribute to the compromised function of E16.5 BM HSPCs (Fig. 3). Also, while adult-like CAR cells are absent from perinatal BM, a subset of osteochondro progenitors appear as significant contributors to the HSPC niche around E18.5/P0, where they provide HSPCs with cues like IGF1/2, which support P0 HSPC function (Fig. 8).

## Discussion

Our investigation of perinatal BM has shed light on this understudied stage of HSC ontogeny by revealing previously unappreciated shifts in HSPC frequency, HSPC function, and niche composition. We discovered that rare, bona fide HSCs colonize fetal bones by E15.5 (Fig. 1) and that FBM HSPCs transition from an MPP2 to an MPP3/4-dominant cell pool by birth (Fig. 2 and Fig. 3). Early FBM HSPCs display little function relative to their time-matched FL counterparts (Fig. 3), which correlates with an absence of key HSC transcriptional signatures, proto-typical supportive populations (e.g. CAR cells) (Figs. 4–7), and a BM niche supportive of HSPC function (Fig. 7c–e). Further, early FBM contains a diverse array of niche cells with little predicted HSPC interaction, as well as signals known suppress HSPC proliferation and differentiation (e.g., TGF-β) (Fig. 8). In contrast, late perinatal BM HSPCs manifest robust function concomitant with the advent of physical niches (*e.g*., osteochondro progenitors) that provide HSPC-supportive signals (e.g., IGF1/2), distinct from the traditional cellular and humoral factors present in the adult BM (Fig. 8).

We also observed three functionally distinct subsets of phLT-HSCs at E16.5: migrating, T cell producing, and inflammed (Fig. 5c and Supplementary Fig. 5c). phLT-HSCs at E16.5 with T-cell transcriptional programs was surprising, although concomitant with a large, transient T cell cluster (Fig. 4b). Thus, these phLT-HSCs may represent early thymic progenitors (ETPs) that derive either from recent FBM HSC arrivals or one of two ETP waves during embryogenesis^70^. phMPP2s displayed less lineage heterogeneity at E16.5 than later timepoints, suggesting enrichment for functional specificity during ontogeny (Fig. 4e). Our data suggests that E16.5 phMPP2s are primarily responding to an unsuitable, inflamed niche (Fig. 5c and Supplementary Fig. 5c)

Surprisingly, FBM MPP2s and LT-HSCs are functionally and transcriptionally distinct from their time-matched FL counterparts (Figs. 3–6), suggesting extrinsic regulation of perinatal BM HSPCs. Indeed, the E16.5 niche can drive differentiation toward T cell lineages while diminishing megakaryocyte production (Fig. 6g and Supplementary Fig. 5e), and we observed dramatic changes in BM stroma composition and ex vivo supportive capacity across ontogeny (Fig. 7). CAR cells were present in adult but absent from fetal and perinatal BM (Supplementary Fig. 6b), as seen previously^14^. We identified physical niches and niche-derived signals for HSPCs throughout this period. Stroma-derived TGF-β was a dominant signal in the E16.5 niche (Fig. 8b, c). While TGF-β signaling induces HSC quiescence^68^, it can also have varying effects: stimulating myeloid-biased HSC proliferation while inhibiting lymphoid-biased HSC growth^71^. Likewise, TGF-β isoforms also have distinct and sometimes pleiotropic effects on HSPCs. High doses of TGF-β2—enriched in our analysis—inhibits HSPC growth while low doses induce proliferation^72^. This fits with our observation that HSPCs in the early FBM display little function (Fig. 3).

We also identified a population of osteochondro progenitors (defined here as osteochondro-4/5) that might contribute to the HSPC niche around birth. These osteochondro progenitors comprised the majority of the E18.5/P0 physical niche (Supplementary Fig. 8e, j) and were enriched for IGF1/2-IGF1R ligand-receptor pairing with HSPCs (Fig. 8d, e). Exogenous IGF1 has been shown to rejuvenate aged HSCs^69^, and we observed that IGF1 and IGF2 are critically required to support HSC during neonatal MSC co-cultures (Fig. 8f–h). The rejuvenating power of IGF1 is consistent with a role for IGF1 and IGF2 during early life, and our data support a model in which IGF1 and IGF2 are key factors that support the establishment of HSC in the early BM.

FBM HSPC shift from an MPP2 to an MPP3/4-dominant phenotype right around birth (Fig. 2a–d). Ambient oxygen can trigger rapid differentiation of LT-HSCs^73^. Could acute exposure to oxygen at delivery explain this shift? As the shift in HSPC frequencies is apparent by E19 (Fig. 2a–d), oxygen exposure is unlikely the cause. At delivery, the neonate also moves from the sterile uterus to a world full of microbes^74^. The adult mouse gut microbiome impacts steady-state hematopoiesis and the production of BM myeloid cells^75^. Germ-free or antibiotic-treated mice have fewer HSPCs, likely due to increased quiescence^76^. Thus, bacterial metabolites (or their effect on the niche or immune cells) may drive HSPC proliferation, which may also contribute to the burst of FBM HSPCs around birth. Finally, hormonal changes in the uterus may also play a role in fetal hematopoiesis; known HSPC regulators like prostaglandins are upregulated around birth and facilitate the onset of labor^77–80^. It is tempting to imagine uterine hormones affecting fetal hematopoietic programs. Further work is needed to explore this hypothesis.

In sum, we have established that the function, frequency, intrinsic programs and extrinsic regulation of BM HSPCs undergoes dramatic shifts and remodeling during fetal and perinatal development. This understudied window of HSC ontogeny is dynamic and subject to regulation distinct from that seen in adult BM, and we have identified niche cells and factors that may control the functional development of the first HSPCs to establish hematopoiesis in the BM. Future studies will further reveal how these distinct niche populations and cues establish the adult hematopoietic hierarchy.

## Methods

### Mice

C57BL/6J, B6.SJL, and UBC-GFP^+/T^ mice were purchased from The Jackson Laboratory and housed in a pathogen-free facility^35^. Transplant recipient mice (F1 progeny of CD45.2 × CD45.1 crosses) were generated at St. Jude Children’s Research Hospital. For all timed pregnancies, 1–2 female CD45.2 mice were placed with a single CD45.2 male overnight, followed by assessment for a vaginal plug before 10 a.m. the next morning. Positive females were separated, and embryos were designated embryonic day 0.5 (E0.5). For all in vivo and ex vivo experiments, male and female mice were used. All animal experiments were carried out according to procedures approved by the St. Jude Children’s Research Hospital Institutional Animal Care and Use Committee and comply with all relevant ethical regulations regarding animal research. Protocol number: 531.

### Isolation of hematopoietic and stromal cells

To isolate hematopoietic cells in adult mice, tibias, femurs, pelvic bones, and spines were removed and bone marrow (BM) was released by crushing in ice-cold PBS, followed by passage through 70μm filters. Cells were then resuspended in red blood cell lysis buffer for 5-10 minutes, washed with PBS/2% fetal calf serum (FCS), and resuspended in PBS/2% FCS. To isolate stomal cells in adult mice, crushed bone fragments were cut into small chips, combined with a portion of the released BM fraction, and placed in pre-warmed digestion media (PBS/2% FCS/0.4% Collagenase II/0.02% DNaseI) followed by gentle shaking (75RPM) at 37 °C for 45 min to 1 h. After digestion, cells were passed through a 70 μm filter and lysed for 5–10 min, washed with PBS/2% FCS, and resuspended in PBS/2% FCS. Dissection of fetal tissues was performed under a dissection microscope in ice cold PBS. Briefly, fetal livers (FL) were removed and placed in ice-cold PBS, followed by removal of all internal tissues and the skin. The fetal skeleton (except the bones of the head) was carefully removed and cleaned of any remaining tissues, washed in PBS, and placed in ice-cold PBS for further processing. The isolation of hematopoietic and stromal cells from the fetal BM (FBM) followed the same protocol used for adult BM with the exclusion of the lysis step, as there was little trace of red blood cells. To isolate hematopoietic cells from FL, livers were gently crushed on a 70 μm filter with the rubber end of a 1 mL syringe while passing ice-cold PBS over the tissue. Following this isolation step, FL hematopoietic cells were lysed, washed with PBS/2% FCS, and resuspended in PBS/2% FCS.

### Bone marrow transplantation

For all experiments, recipient mice (CD45.2/CD45.1) were lethally irradiated with two doses of 5.8 Gy on the same day as transplantation. Donor (CD45.2) and support (CD45.1) BM cells were isolated and counted using a hemacytometer, with trypan blue used to assess viability. For whole BM (WBM) transplantation experiments, 1.5 × 10^6^ donor WBM cells were combined with 2 × 10^5^ support WBM cells and intravenously injected into the tail vein of recipient mice. Recipients were maintained on 0.25 mg/mL enrofloxocin in drinking water for 10 days following injection. For limiting cell transplants of UBC-GFP^+/T^ multipotent progenitor 2 (MPP2) cells, 100 donor MPP2s were sorted into 1.5 mL tubes containing PBS/2% FCS, 2 × 10^5^ sorted support WBM cells were added, and the entire cell suspension was injected intravenously into the tail vein of recipient mice. The same method was used for limiting cell transplants of 10 LT-HSCs. All sorts were performed on a BD FACSAria cell sorter. For secondary transplantation, five aliquots of 5 × 10^6^ WBM cells were isolated from a single primary recipient and injected into the tail vein of five lethally irradiated secondary recipients.

### Peripheral blood analysis

Transplant recipients were periodically assessed for donor cell contribution to the PB. PB was collected from the retro-orbital plexus in heparinized capillary tubes, followed by lysis in red blood cell lysis buffer. Cells were then resuspended in PBS/2% FCS and analyzed by flow cytometry for donor and lineage contribution on a BD LSR Fortessa. For WBM primary and secondary transplants and separate bone transplants, PB was stained with CD45.1-FITC, CD45.2-v500, [B220, CD11b, Gr-1]-PerCP-Cy5.5, and [B220, CD4, CD8]-PECy7. The myeloid lineage was considered CD11b^+^Gr-1^+^, the B cell lineage was considered B220^+^, and the T-cell lineage was considered CD4^+^CD8^+^. For MPP2 limiting cell transplants, we also included a separate analysis of non-lysed PB stained with CD41-PerCPe710 (platelets) and Ter119-PECy7 (erythrocytes). All data were analyzed using FlowJo version 10 (Treestar).

### Recipient BM analysis

At a terminal (>20 weeks) timepoint after transplantation, recipients of WBM were euthanized and hematopoietic cells were isolated from the BM. Cells were assessed via flow cytometry on a BD LSR Fortessa for the presence of donor-derived CMPs (Lineage^−^c-Kit^+^Sca-1^−^CD34^+^CD32/16^lo^), GMPs (Lineage^−^c-Kit^+^Sca-1^−^CD34^+^CD32/16^hi^), MEPs (Lineage^−^c-Kit^+^Sca-1^−^CD34^−^CD32/16^−^), CLPs (Lineage^−^c-Kit^mid^Sca-1^mid^CD127^+^), LT-HSCs, (Lineage^-^Sca-1^+^c-Kit^+^ (LSK)Flt3^−^CD48^−^CD150^+^), ST-HSCs (LSKFlt3^−^CD48^−^CD150^−^), MPP2s (LSKFlt3^−^CD48^+^CD150^+^), MPP3s (LSKFlt3^−^CD48^+^CD150−), and MPP4s (LSKFlt3^+^CD48^+^CD150^−^). In all cases, the Lineage^−^ fraction showed low expression of CD4, CD8, B220, Gr-1, and Ter119. All data were analyzed using FlowJo version 10 (Treestar).

### Analysis of hematopoietic stem and progenitors across bone marrow development

Liver and entire skeleton BM hematopoietic cells were isolated from E15.5-postnatal day 0 (P0), P2, P4, P6, P8, P14, P21, and P28 CD45.2 mice and assessed via flow cytometry on a BD LSR Fortessa for the presence of LT-HSCs, ST-HSCs, MPP2s, MPP3s, and MPP4s. All data were analyzed using FlowJo version 10 (Treestar).

### CXCR4 cell-surface expression

Hematopoietic cells from E15.5-P0 livers and P0 FBM were isolated and assessed via flow cytometry on a BD LSR Fortessa for the presence of LT-HSCs, ST-HSCs, MPP2s, MPP3s, and MPP4s, as well as cell-surface CXCR4. All data were analyzed using FlowJo version 10 (Treestar).

### CXCL12 migration assays

FL LSK, LT-HSCs, ST-HSCs, MPP2s, MPP3s, and MPP4s were sorted on a BD Aria cell sorter and dispensed into the top portion of a 24-well trans-well plate containing StemSpan^TM^ Serum-Free Expansion Medium (SFEM, STEMCELL Technologies) with no supportive cytokines. Meanwhile, the bottom portion of each trans-well contained either 0 ng/mL or 100 ng/mL soluble CXCL12 in SFEM. After a 5-hour incubation, the bottom portion of the trans-well was collected and plated in 3 mL M3434 Methocult (STEMCELL Technologies) to determine colony-forming unit (CFU) potential. A known number of sorted cells (which were not subject to the trans-well assay) were also plated into M3434 Methocult to determine the input CFU frequency. After 10–12 days, CFUs were counted, and the migration potential of each population was calculated using the following formula:

(# of migratory CFU)/[(Frequency of input CFU) * (# input cells used in trans-well assay)].

To compare FL versus FBM HP migration potential, 200,000 c-Kit-enriched P0 FL or FBM cells were plated in 24-well trans-well plates with or without CXCL12, as detailed above. After 16 h, wells were harvested, and the number of migrated cells assessed by flow cytometry (BD LSR Fortessa).

### Single-cell CFU assays

Single MPP2s from E16.5-P0 FL/FBM and adult BM were sorted on a BD Aria cell sorter directly into 96-well plates containing 100 μL of M3231 Methocult (STEMCELL Technologies) supplemented with 25 ng/mL SCF, 25 ng/mL Flt3L, 25 ng/mL IL-11, 10 ng/mL IL-3, 10 ng/mL GM-CSF, 25 ng/mL TPO, and 4 U/mL EPO^9^. After 10-12 days of culture, wells were scored for the presence and phenotype of CFUs.

### Mesenchymal stromal cell cultures

To establish MSC cultures, bone marrow was collected from whole skeletons at E16.5 or P0 (see details above), and CD45^+^ cells were depleted via MojoSort anti-mouse CD45 beads (Biolegend). Cells were then washed three times with PBS/2% FCS and cultured in αMEM/10% FCS. 24 h later, non-adherent cells were removed and the remaining stroma monolayer was washed with PBS and then maintained in αMEM/10% FCS. Cells were passaged at 80% confluence. Passage 3 (P3) cultures were used for all subsequent experiments.

### BM MSC differentiation assay

Seventy thousand P3 BM MSCs were tested for adipogenic, osteogenic, or chondrogenic differentiation potential using StemPro MSC Differentiation Kits (Gibco) and culturing for seven, 14 and 21 days, respectively. Media was changed 2–3 times/week. The resulting cultures were then stained with Oil Red O (adipogenic), Alizarin Red S (osteogenic), and Alcian Blue (chondrogenic) to determine differentiation potential.

### Enzyme-linked immunosorbent assay (ELISA)

To measure IGF1 and IGF2 levels in culture supernatants, 100,000 E16.5 or P0 BM MSCs were cultured in 1 mL of PVA-based media. 24 h later, supernatant was collected, centrifuged at 500 × *g* for 5 min to remove cell debris and then assayed using the IGF1/IGF2 Mouse Elisa Kit (Invitrogen) according to the manufacturer’s instructions.

### LT-HSCs and BM MSC co-cultures

For co-culture experiments, 100,000 P3 BM MSCs were plated in 12-well tissue-culture treated plates in αMEM/10% FCS. After 24 h, cells were washed with PBS and 1000 E16.5 or P0 FL LT-HSCs (LSK CD150^+^CD48^−^) were added to the cultures. Cultures were maintained in polyvinyl alcohol (PVA, Sigma) supplemented with HEPES, Insulin-Transferrin-Selenium-X, mTPO and mSCF^81^. After 8–9 days, the number of CD45^+^ cells, LSK cells, and LT-HSCs (c-Kit^+^Sca1^+^EPCR^+^CD150^+^)^82^ were analyzed by flow cytometry to determine the relative expansion of each cell type. Here, 200 c-Kit^+^Sca1^+^EPCR^+^ CD150^+^ cells were also isolated from cultures via FACS and plated into methylcellulose (M3434) to interrogate CFU potential.

For knockdown of IGF1 and IGF2 in P0 BM MSCs, 100,000 P0 MSCs/well were plated in 12-well plates in 10% FCS αMEM media. After 24 h, the media was replaced with 1 mL fresh αMEM media. Fifty nanomolar IGF1, IGF2, IGF1 and IGF2, or control siRNA (Horizon Discovery) suspended in 1 mL of Darmafect transfection reagent (Horizon Discovery) was then titrated to each well. After 24 h, media was removed and 1000 P0 FL LT-HSCs added in PVA-based media. HSCs and MSCs were co-cultured for another 8 days and then analyzed as described for CD45+ cells, HSPCs and phenotypic HSCs. IGF1 and IGF2 knockdown efficiency was confirmed with ELISA one, four and seven days post-siRNA treatment.

### FL LT-HSCs differentiation assay

Ten thousand E16.5 or P0 BM MSCs/well were seeded into 96-well plates. After 24 h, 50 E16.5 or P0 FL LT-HSCs were added in StemSpan SFEM (Stem Cell Technologies) supplied with 10% FCS. Eight days later, cells were harvested, stained with antibodies for CD45, CD3, CD19, Gr-1, CD11b, CD41, Ter119 and DAPI, and analyzed by flow cytometry (BD LSR Fortessa). The resulting data were analyzed using FlowJo version 10 (Treestar).

### Single-cell RNA sequencing

E16.5, E18.5, P0, and adult HP (CD45^+^Lineage^−^c-Kit^+^) and stromal cells (CD45^−^Ter119^−^) were isolated from BM as described above and sorted on a BD Aria cell sorter. Prior to sorting, cells were stained with a panel of CITE-Seq antibodies for downstream sequencing analyses. mRNA expression libraries were constructed according to the Chromium single cell 3′ reagent v3.1 protocol, while CITE-Seq expression libraries were constructed according to the protocol described in ref. 36. Libraries were sequenced using Illumina NovaSeq paired-end sequencing per the 10X protocol to a minimum depth of 50,000 reads per cell. Each library represents a pool of HP or stromal cells collected from 38 (E16.5), 9 (E18.5), 6 (P0), and 4 (Adult) mice. For E16.5 to P0 samples, mice were pooled from 2 to 3 litters.

### Analysis of single-cell data

Following Illumina sequencing, raw data was processed with CellRanger (v3.1.0, 10X Genomics) using the corresponding mouse reference (mm10 v3.0.0) using the ‘count’ and ‘aggregate’ functions with default settings. Aggregated count matrices were imported into R version 4.0 and all analysis was performed using the Seurat R package version 3.1.5^83^. All genes present in at least 30 cells were retained for analysis. Each sample was filtered separately, excluding cells with ≥ the 98^th^ percentile of the number of genes per cell or the number of UMIs per cell (to exclude putative doublets), and also excluding cells with≥ 7.5% mitochondrial expression or less than 300 UMIs (to exclude putatively dead or dying cells). Expression data were log-normalized using default parameters, whereas CITE-Seq data were normalized using the “CLR” method. We identified variable features using the ‘vst’ method in Seurat, again with default parameters. Cell cycle phases were inferred using the CellCycleScoring function in Seurat, with phase-specific markers obtained from ref. 84. Variable features were scaled by regressing out the effects of per cell UMI count, percent of mitochondrial expression, and inferred S-phase and G2M-phase scores. Scaled variable features were then used for Principal Component Analysis and UMAP analysis. All libraries were processed identically, and UMAP clustering based on library, developmental timepoint, and cell type confirmed no batch effects. Clustering was performed according to the default parameters in Seurat. The top 2000 variable features and different numbers of PCA dimensions were used for the clustering. The numbers of dimensions were determined based on the elbow plot to represent each data subset and varied from 18-26. To annotate clusters, we used the FindAllMarkers function in Seurat to provide a list of gene expression comparisons among clusters, as well as the percentage of cells in each cluster with significant levels of gene expression. We also utilized ROC-test based statistics to define the key genetic markers in each cluster. Finally, we compared the gene expression patterns seen in our clusters to previously compiled single cell RNA-Sequencing datasets of adult BM stroma and hematopoietic cells^37–44,46–48,50,51,57–62,85,86^. All UMAP and expression plots were generated using packages in R Version 4.0. Contaminating blood cells were removed from the processed objects shown in Fig. 7. The CAR-specific expression signature was defined using adult CAR marker genes and further filtered based on adjusted *p* value < 0.05 and the difference in ‘pct1’ (the adult CAR population) and ‘pct2’ (all other cell types) >0.5.

### Merged phLT-HSCs and phMPP2s

FBM phLT-HSCs and phMPP2s from E16.5, E18.5, P0, and adult were combined using the Seruat (v3.2.2) merge function. Variable features (*n* = 2000) were defined for the merged dataset using the vst selection method. Data were scaled using default parameters and PC dimensions 1–10 (phLT-HSCs) and 1–20 (phMPP2s) were used for UMAP visualization.

### Comparative analysis of FBM and FL LT-HSCs and HPs

FBM LT-HSCs were combined with E16.5 and P0 FL using the Seurat (v3.2.2) merge function. Variable features (*n* = 2000) were defined for the merged dataset using the vst selection method^54^. Data were scaled using default parameters and PC dimensions 1–20 were used for UMAP visualization. The UMAP plot was further split based on cell type identity. Marker genes were identified between FBM and FL timepoints using a log2FC threshold > 0.5 and min.pct = 0.25. E16.5 FBM HPs (Lin^-^c-Kit^+^) were integrated with E16.5 FL HPs using Seurat (v3.2.2) FindIntegrationAnchors and IntegrateData functions and dimensions 1–30 and the default assay was set to “integrated”^54^. Next, data were scaled using default parameters and runPCA/runUMAP were run using dimensions 1–20. Final UMAP visualizations were split by compartment.

### RNA-Magnet

RNA-Magnet (v.0.1.0) was applied to the single-cell gene expression data. The R package was downloaded from a GitHub repository (https://github.com/veltenlab/rnamagnet) and was imported into R version 4.0^38^. The MAGIC (Markov affinity-based graph imputation of cells) package is embedded in the package and performed before RNA-Magnet for denoising and filling in putatively missing transcripts of the single-cell gene expression matrix^87^. The RNAMagnetAnchors function was then used to identify physical niches, and calculates adhesiveness, specificity scores, and the direction of the given cells. Here, the adhesiveness indicates overall strength of an interaction, the specificity is an interaction score to the anchor populations, and the direction represents one of the populations that a given cell is most attracted to. The RNAMagnetSignaling function was subsequently used to compute interaction scores for secreted ligand-receptor pairs among different cell types. We also derived p-values of the interactions scores with the permutation test described below.

### Trajectory and GO analyses

Principal components (PCs) were generated using the RunPCA function in Seurat R package version 3.2.3 (npcs = 10) and used as an input for Slingshot trajectory analysis (v1.6.1)^88^. For GO (Gene Ontology) analysis, we applied the FindMarkers function in the Seurat R package (v3-4) to extract differentially expressed genes between two cell subsets, and then performed the enrichGO function of clusterProfiler (v3.16.1)^89^. For example, to determine genes to analyze for P0-rich GO terms for phLT-HSCs (Fig. 5A), we identified the genes with positive average logFC values with an adjusted *p* value < 0.05 and difference in “pct1” (P0-rich cells) and “pct2” (all other cells) >0.

### Statistical comparisons

Experimental results are reported as mean ± SD or SE, as indicated in the figure legends. Sample sizes and numbers of replicates, are also included in the legends. Indication of significance shown in Figs. 1–4, 6–8, and Supplementary Figs. 2, 3, 5, and 8 were all derived from two-tailed Wilcoxon Signed Rank Tests or two-tailed Mann-Whitney Tests. To determine statistical significance of Specificity and Interaction Scores for RNA-Magnet, we performed permutation tests with RNA-Magnet on randomized anchors, where the cell types were randomly assigned, keeping the number of cells in each random anchor consistent with that in each original anchor. P-values were computed from the permutations and corrected for multiple testing based on the bonferroni approach.

### Reagents

All reagents and resources, including their vendor, catalog numbers, and dilutions (for antibodies) can be found in Supplementary Data 3.

### Reporting summary

Further information on research design is available in the Nature Research Reporting Summary linked to this article.

## Supplementary information

Supplementary Information

Peer Review File

Description of Additional Supplementary Files

Supplementary Data 1

Supplementary Data 2

Supplementary Data 3

Reporting Summary

## Data Availability

The single-cell RNA-sequencing datasets generated during this study have been deposited to GEO under accession number GSE178951. Published scRNA-Seq data used in this paper can be found under accession number GSE128761 and GSE122467. All accession numbers are listed in Supplementary Data 3. Source data are provided with this paper.

## Source data

Source Data

## Code availability

All original code has been deposited at GitHub [https://github.com/trenthall612/Hall-et-al] and is publicly available as of the date of publication.
