# Supplementary material for: Murine fetal bone marrow does not support functional hematopoietic stem and progenitor cells until birth
Source: Nat Commun. 2022 Sep 15;13:5403. doi: 10.1038/s41467-022-33092-4 (PMC9477881; doi:10.1038/s41467-022-33092-4)
Supplement: Supplementary file 1 — Supplementary Information [file 41467_2022_33092_MOESM1_ESM.pdf]

Supplementary Information.

Supplementary Figures 1-8

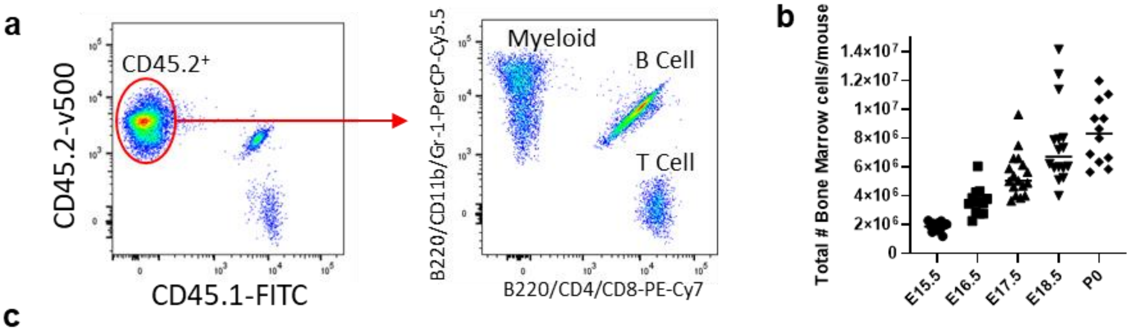

c

	E15.5	E16.5	E17.5	E18.5	P0	Adult
E15.5	1	0.274965	0.001228	1.05E-05	1.62E-06	4.19E-05
E16.5	0.274965	1	0.002163	9.72E-06	2.58E-08	4.89E-06
E17.5	0.001228	0.002163	1	0.000766	1.08E-09	0.000247
E18.5	1.05E-05	9.72E-06	0.000766	1	0.000207	0.165682
P0	1.62E-06	2.58E-08	1.08E-09	0.000207	1	0.000294
Adult	4.19E-05	4.89E-06	0.000247	0.165682	0.000294	1

d

	E15.5	E16.5	E17.5	E18.5	P0	Adult
E15.5	1	0.573737	0.749996	0.569891	0.286725	0.541423
E16.5	0.573737	1	0.449334	0.525171	0.245045	0.321267
E17.5	0.749996	0.449334	1	0.970935	0.154977	0.382947
E18.5	0.569891	0.525171	0.970935	1	0.0576	0.438946
P0	0.286725	0.245045	0.154977	0.0576	1	0.928699
Adult	0.541423	0.321267	0.382947	0.438946	0.928699	1

e

	E15.5	E16.5	E17.5	E18.5	P0	Adult
E15.5	1	0.441803	0.523936	0.811144	0.561179	0.470214
E16.5	0.441803	1	0.620955	0.232183	0.087332	0.112131
E17.5	0.523936	0.620955	1	0.28253	0.030163	0.026833
E18.5	0.811144	0.232183	0.28253	1	0.057539	0.040529
P0	0.561179	0.087332	0.030163	0.057539	1	0.107328
Adult	0.470214	0.112131	0.026833	0.040529	0.107328	1

f

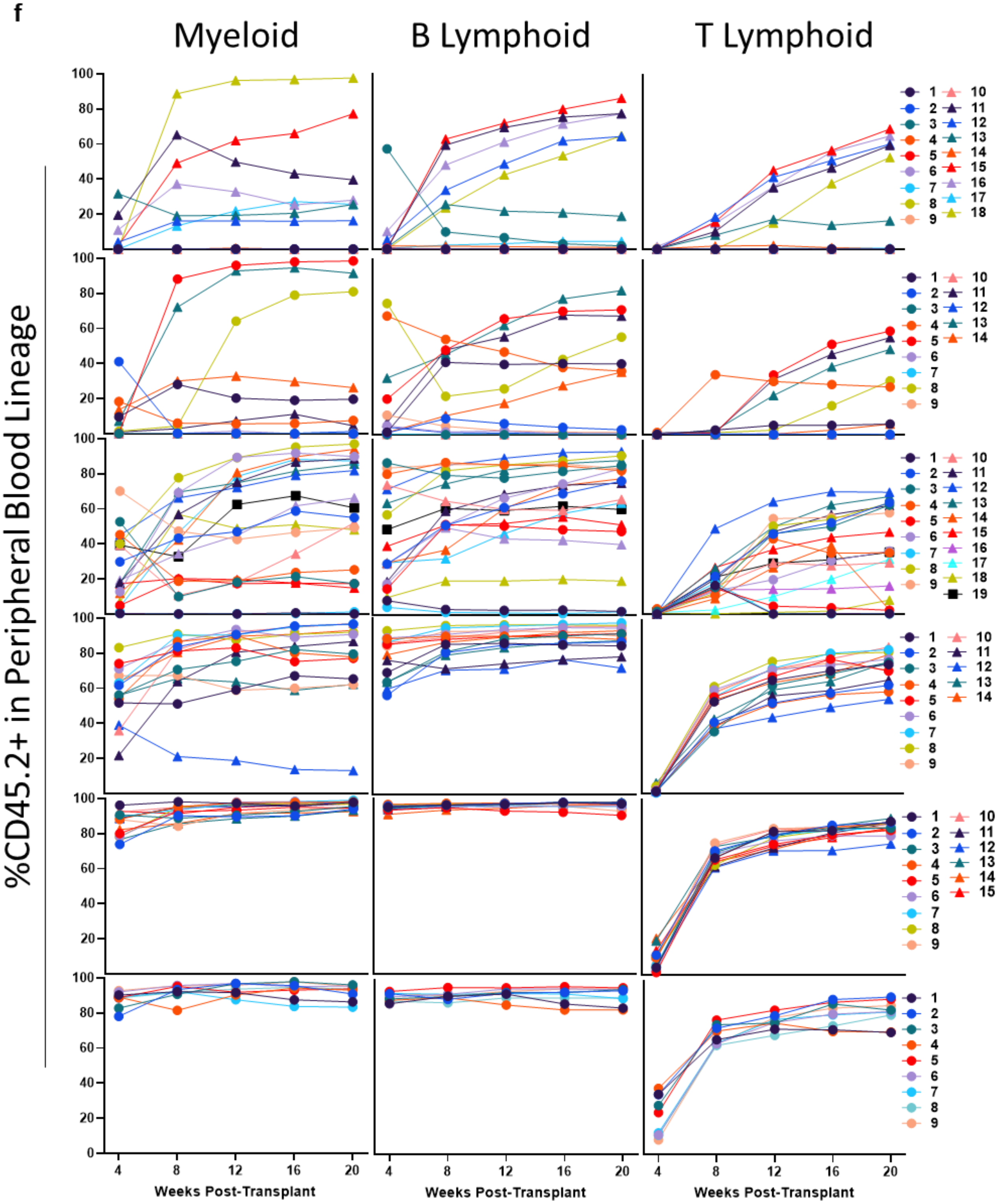

Myeloid
B Lymphoid
T Lymphoid
%CD45.2+ in Peripheral Blood Lineage
Weeks Post-Transplant
Weeks Post-Transplant
Weeks Post-Transplant

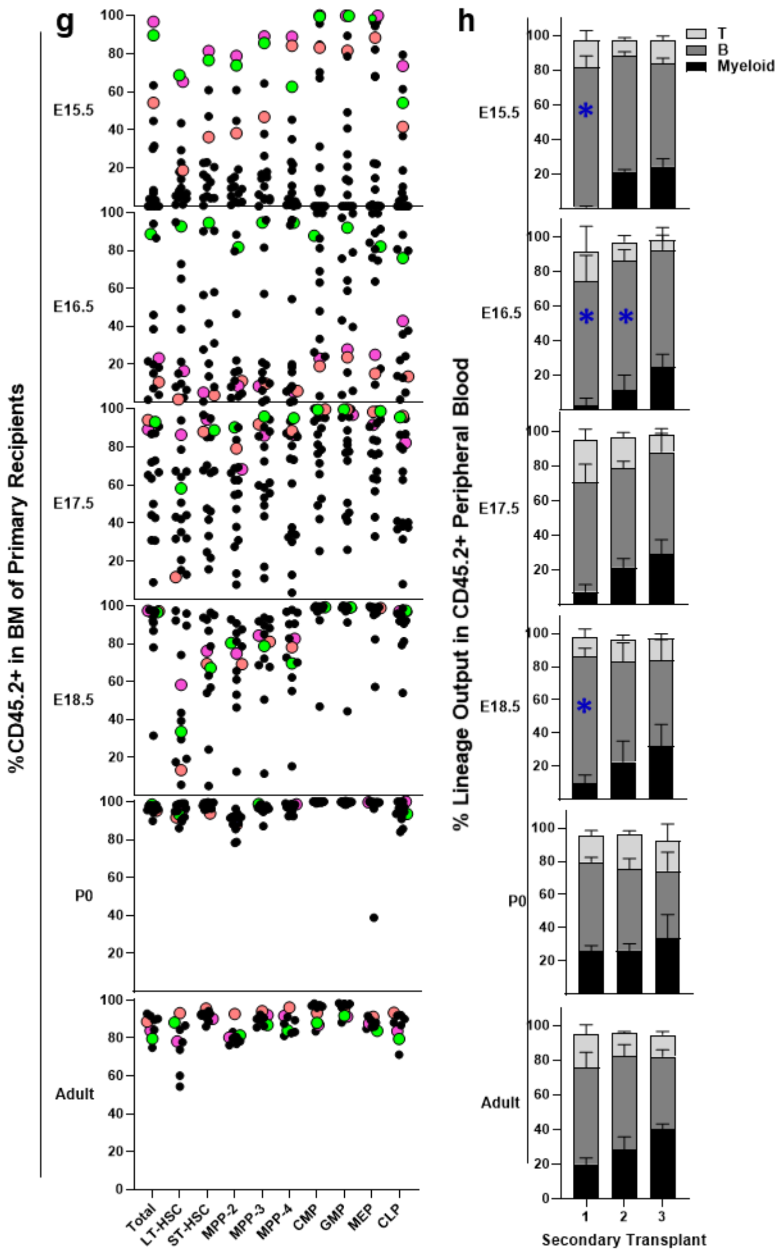
g
h
% CD45.2+ in BM of Primary Recipients
E15.5
E16.5
E17.5
E18.5
P0
Adult
Total
LT-HSC
ST-HSC
MPP-2
MPP-3
MPP-4
CMP
GMP
MEP
CLP
T
B
Myeloid
% Lineage Output in CD45.2+ Peripheral Blood
Secondary Transplant
1
2
3

Supplementary Figure 1 (related to Fig. 1). PB gating strategy, perinatal BM cellularity, BM HSPC chimerism, and PB lineage and chimerism data for transplants in Fig. 1. a. Gating strategy for PB analyses in Fig. 1b-c. b. Total number of WBM cells per embryo at each developmental timepoint (n = 10-17 embryos from 3 independent litters). c. P-Values for Wilcoxon signed-rank test comparing average CD45.2+ % from Figure 1b at 20 weeks post-transplant. d. P-Values for Wilcoxon signed-rank test comparing average Myeloid % from Figure 1c at 20 weeks post-transplant. e. P-Values for Wilcoxon signed-rank test comparing average B Cell % from Figure 1b at 20 weeks post-transplant. f. %CD45.2+ cells within myeloid, B lymphoid, and T lymphoid cells 4-20 weeks post-transplant. Related to Fig. 1b. g. Donor contribution to various progenitors in primary recipient bone marrow. Each dot represents a single mouse, with chimerism for each mouse displayed across total BM and BM HSPC subsets. Colored dots represent primary recipients chosen for secondary transplants (n = 9-18). h. Lineage output in secondary transplant cohorts. Data represent means and standard deviation (n = 3 secondary transplants, 5 mice/transplant); b-lymphoid bias (%B cell > 70%), blue asterisk. P-Values determined by Mann-Whitney Test, two-tailed. HSPC, hematopoietic stem and progenitors; PB, peripheral blood; BM, bone marrow; HSC, hematopoietic stem cell; LT-HSC, long-term HSC; ST-HSC, short-term HSC; MPP, multipotent progenitor; CMP, common myeloid progenitor; GMP, granulocyte-macrophage progenitor; MEP, megakaryocyte-erythroid progenitor; CLP, common lymphoid progenitor. Source data are provided in the Source Data File.

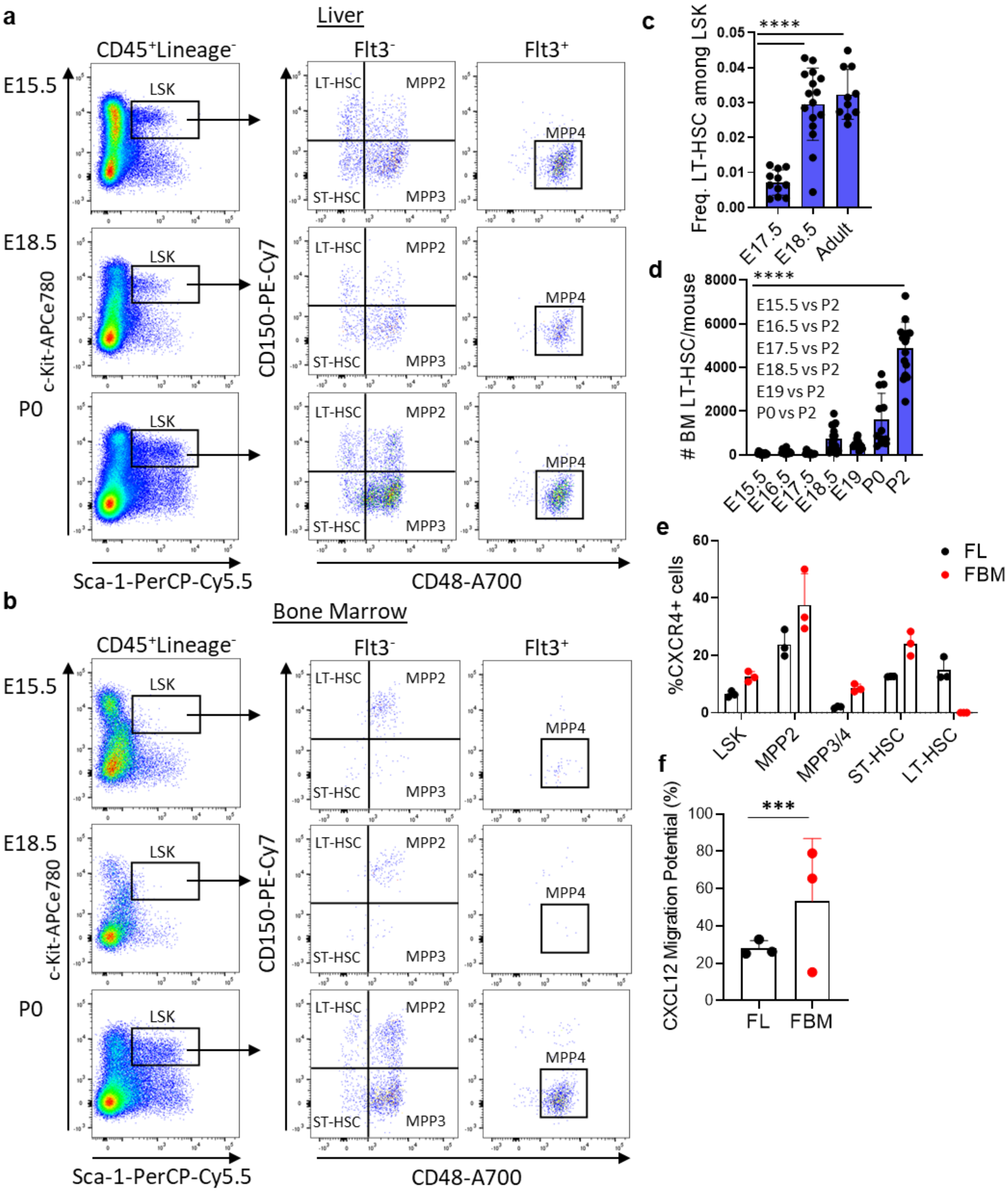
a
Liver
CD45+Lineage-
Flt3-
Flt3+
E15.5
E18.5
P0
LSK
LT-HSC
MPP2
ST-HSC
MPP3
MPP4
c-Kit-APCe780
CD150-PE-Cy7
Sca-1-PerCP-Cy5.5
CD48-A700
b
Bone Marrow
c
Freq. LT-HSC among LSK
****
E17.5
E18.5
Adult
d
BM LT-HSC/mouse
****
E15.5 vs P2
E16.5 vs P2
E17.5 vs P2
E18.5 vs P2
E19 vs P2
P0 vs P2
E15.5
E16.5
E17.5
E18.5
E19
P0
P2
e
%CXCR4+ cells
FL
FBM
LSK
MPP2
MPP3/4
ST-HSC
LT-HSC
f
CXCL12 Migration Potential (%)
***
FL
FBM

Supplementary Figure 2 (related to Fig. 2). HSPC gating strategy for perinatal liver and BM and CXCL12 migration potential of P0 BM. Gating strategy for HSPCs (LT-HSCs, ST-HSCs, MPP2s, MPP3s, MPP4s) in (a) fetal liver and (b) fetal bone marrow. c. Frequency of immunophenotypic LT-HSCs in E17.5, E18.5, and adult BM (n = 10-16 embryos from 2-3 independent litters). d. Total number of immunophenotypic LT-HSCs in E15.5 per mouse in E15.5 through P2 BM (n = 10-16 embryos from 2-3 independent litters). e.%P0 BM or P0 FL HSPCs expressing CXCR4 (n = 3). f. CXCL12 migration potential of P0 FL vs P0 FBM HPs (n = 3, P = 0.001). Data represent mean and standard deviation. ****, $P \leq 0.0001$. P-values determined by Mann-Whitney Test, two-tailed. HSPC, hematopoietic stem and progenitor cells; BM, bone marrow; FL, fetal liver; FBM, fetal BM; LSK, $Lineage^{-}Sca1^{+}c\text{-}Kit^{+}$; HSC, hematopoietic stem cell; LT-HSC, long-term HSC; ST-HSC, short-term HSC; MPP, multipotent progenitor. Source data are provided in the Source Data File.

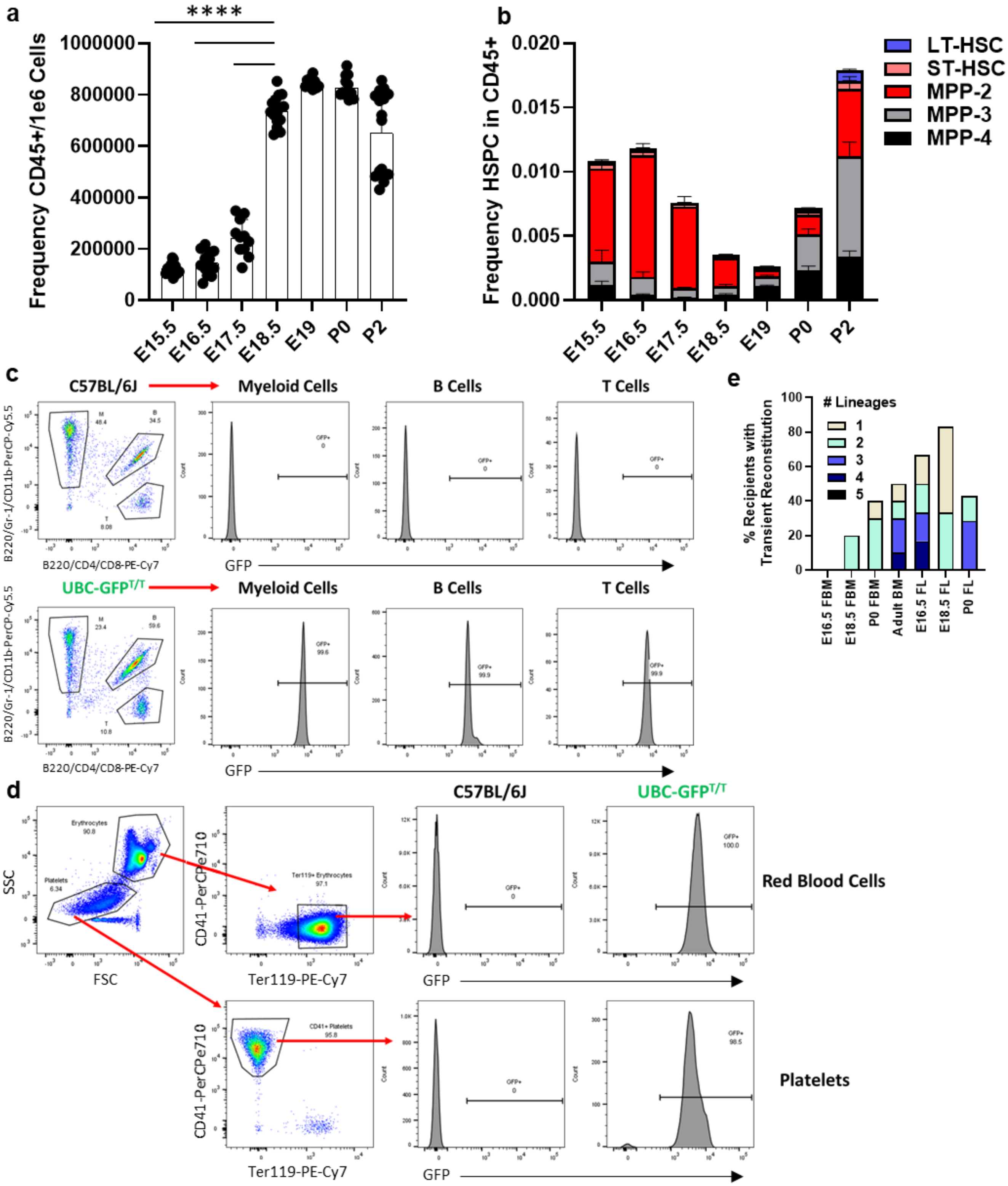
a
****
Frequency CD45+/1e6 Cells
1000000
800000
600000
400000
200000
0
E15.5
E16.5
E17.5
E18.5
E19
P0
P2
b
Frequency HSPC in CD45+
0.020
0.015
0.010
0.005
0.000
LT-HSC
ST-HSC
MPP-2
MPP-3
MPP-4
c
C57BL/6J
Myeloid Cells
B Cells
T Cells
B220/Gr-1/CD11b-PerCP-Cy5.5
B220/CD4/CD8-PE-Cy7
GFP
UBC-GFP T/T
d
Erythrocytes
Platelets
SSC
FSC
CD41-PerCPe710
Ter119-PE-Cy7
Ter119+ Erythrocytes
CD41+ Platelets
C57BL/6J
UBC-GFP T/T
Red Blood Cells
Platelets
e
% Recipients with Transient Reconstitution
# Lineages
1
2
3
4
5
E16.5 FBM
E18.5 FBM
P0 FBM
Adult BM
E16.5 FL
E18.5 FL
P0 FL

Supplementary Figure 3 (related to Fig. 3). Changes in blood and HSPC frequencies in perinatal BM, and PB characterization of *UBC*-GFP mice. The frequency of (a) $CD45^+$ cells in the bone marrow or (b) HSPCs in $CD45^+$ BM from embryos and neonates (n = 10-16 embryos from 2-3 independent litters). All data represent means ± SE. Representation of GFP expression in the (c) myeloid, B cell, T cell, (d) erythrocyte, and platelet PB compartments in C57BL/6J and UBC-$GFP^{T/T}$ mice. (e). % Recipients with transient PB donor reconstitution in Fig. 3e-f (n = 5-10 recipients). ****, $P < 0.0001$. P-values determined by Mann-Whitney Test, two-tailed. HSPC, hematopoietic stem and progenitor cells; BM, bone marrow; PB, peripheral blood; FL, fetal liver; FBM, fetal BM; HSC, hematopoietic stem cell; LT-HSC, long-term HSC; ST-HSC, short-term HSC; MPP, multipotent progenitor. Source data are provided in the Source Data File.

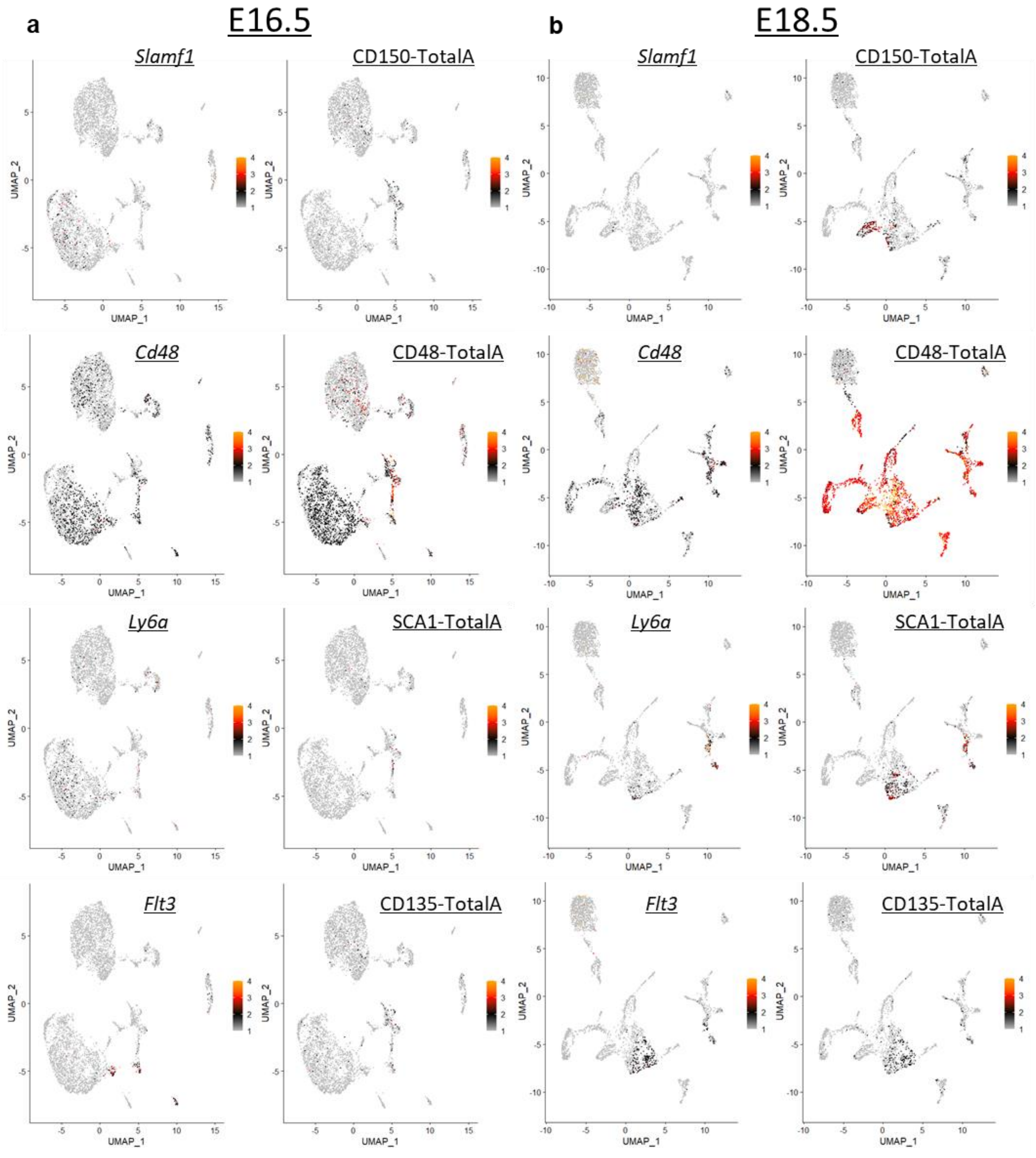

a
E16.5
b
E18.5
Slamf1
CD150-TotalA
Cd48
CD48-TotalA
Ly6a
SCA1-TotalA
Flt3
CD135-TotalA
UMAP_1
UMAP_2

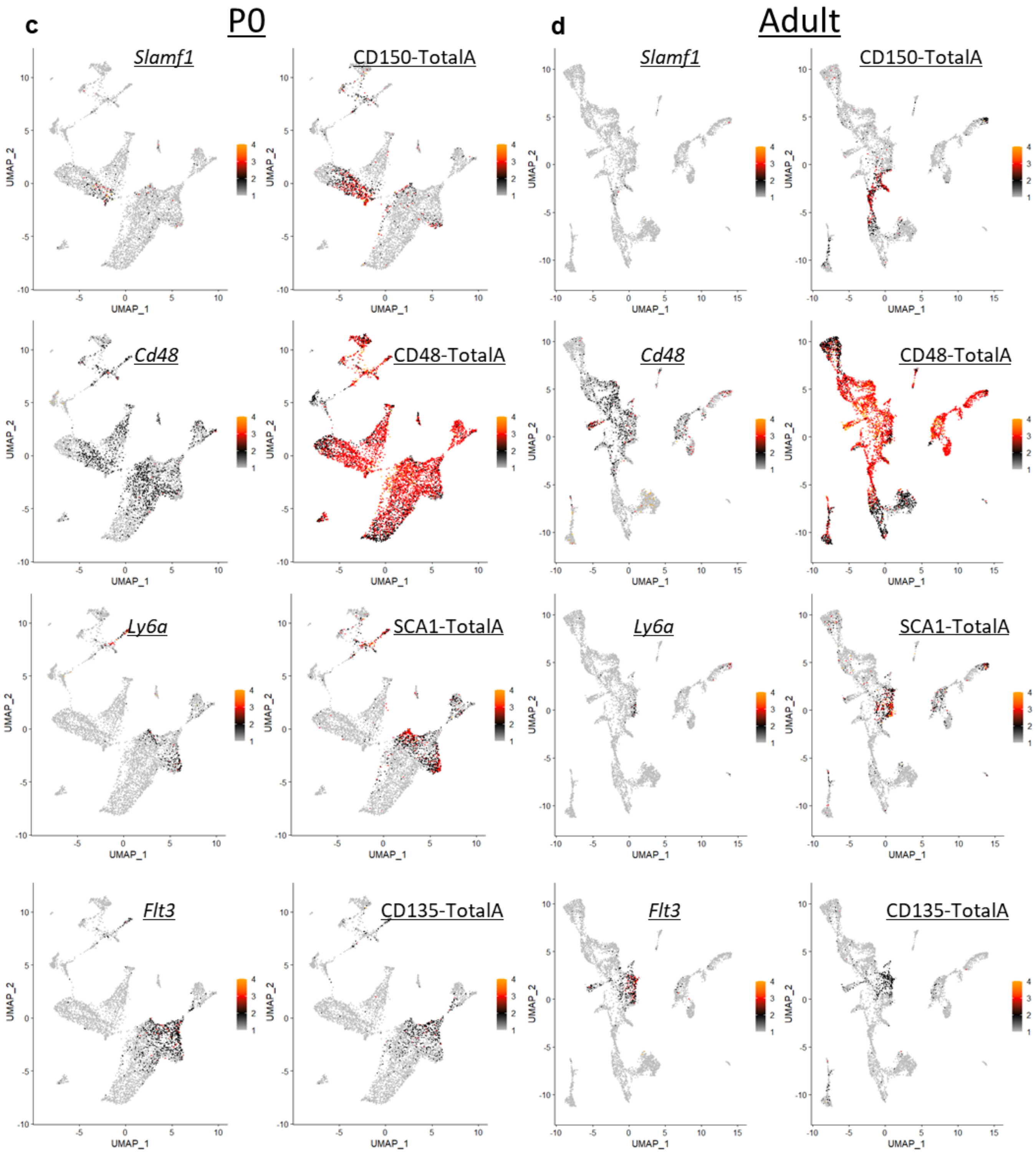
c
P0
d
Adult
Slamf1
CD150-TotalA
Cd48
CD48-TotalA
Ly6a
SCA1-TotalA
Flt3
CD135-TotalA
UMAP_1
UMAP_2

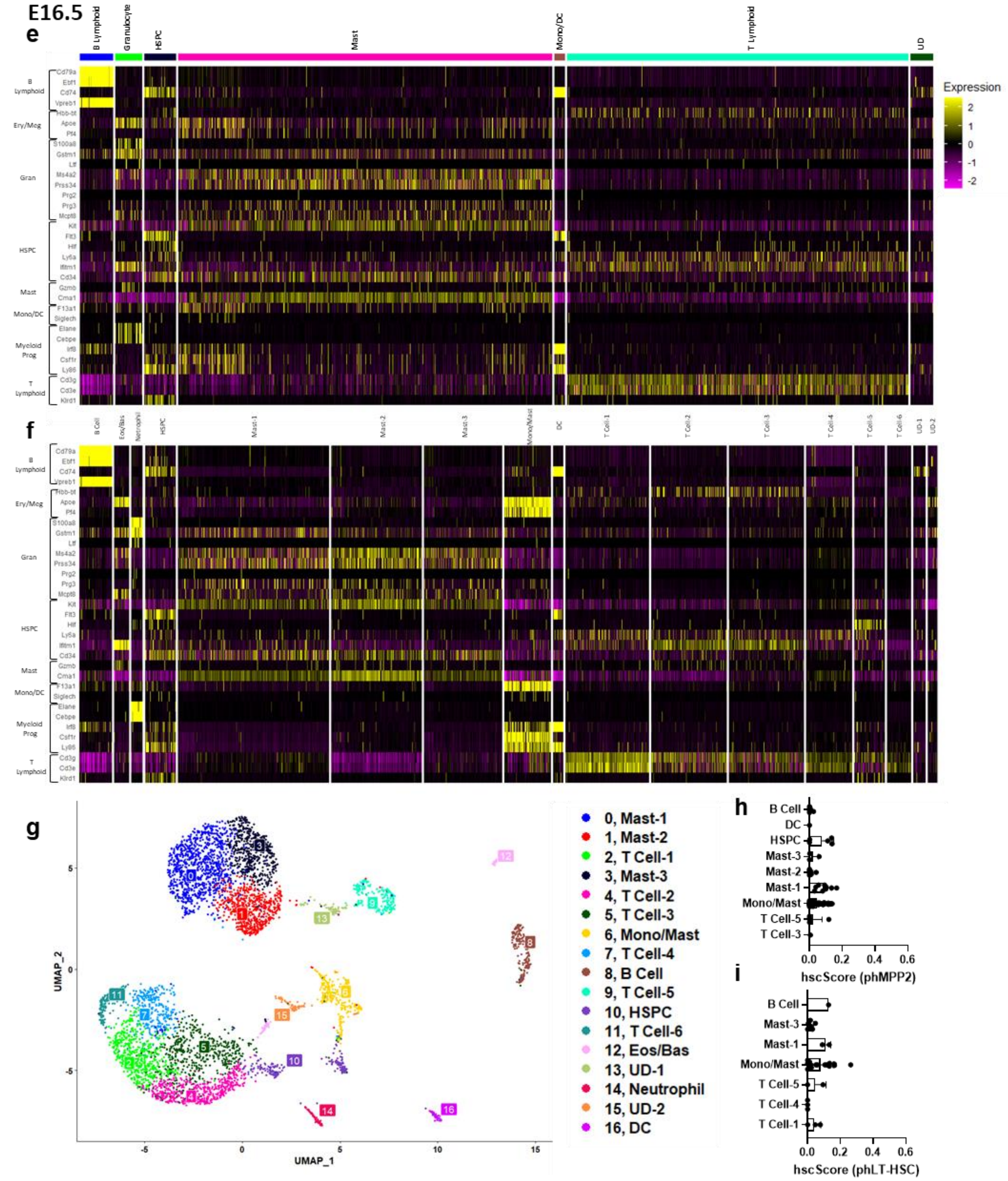

E16.5
e
B Lymphoid
Granulocyte
HSPC
Mast
Mono/DC
T Lymphoid
UD
Expression
B Lymphoid
Ery/Meg
Gran
HSPC
Mast
Mono/DC
Myeloid Prog
T Lymphoid
Cd79a
Ebf1
Cd74
Vpreb1
Hbb-bt
Apoe
Pf4
S100a8
Gstm1
Ltf
Ms4a2
Prss34
Prg2
Prg3
Mcpt8
Kit
Flt3
Hlf
Ly6a
Ifitm1
Cd34
Gzmb
Cma1
F13a1
Siglech
Elane
Cebpe
Irf8
Csf1r
Ly86
Cd3g
Cd3e
Klrd1
f
B Cell
Eos/Bas
Neutrophil
HSPC
Mast-1
Mast-2
Mast-3
Mono/Mast
DC
T Cell-1
T Cell-2
T Cell-3
T Cell-4
T Cell-5
T Cell-6
UD-1
UD-2
g
UMAP_1
UMAP_2
0, Mast-1
1, Mast-2
2, T Cell-1
3, Mast-3
4, T Cell-2
5, T Cell-3
6, Mono/Mast
7, T Cell-4
8, B Cell
9, T Cell-5
10, HSPC
11, T Cell-6
12, Eos/Bas
13, UD-1
14, Neutrophil
15, UD-2
16, DC
h
B Cell
DC
HSPC
Mast-3
Mast-2
Mast-1
Mono/Mast
T Cell-5
T Cell-3
hscScore (phMPP2)
i
B Cell
Mast-3
Mast-1
Mono/Mast
T Cell-5
T Cell-4
T Cell-1
hscScore (phLT-HSC)

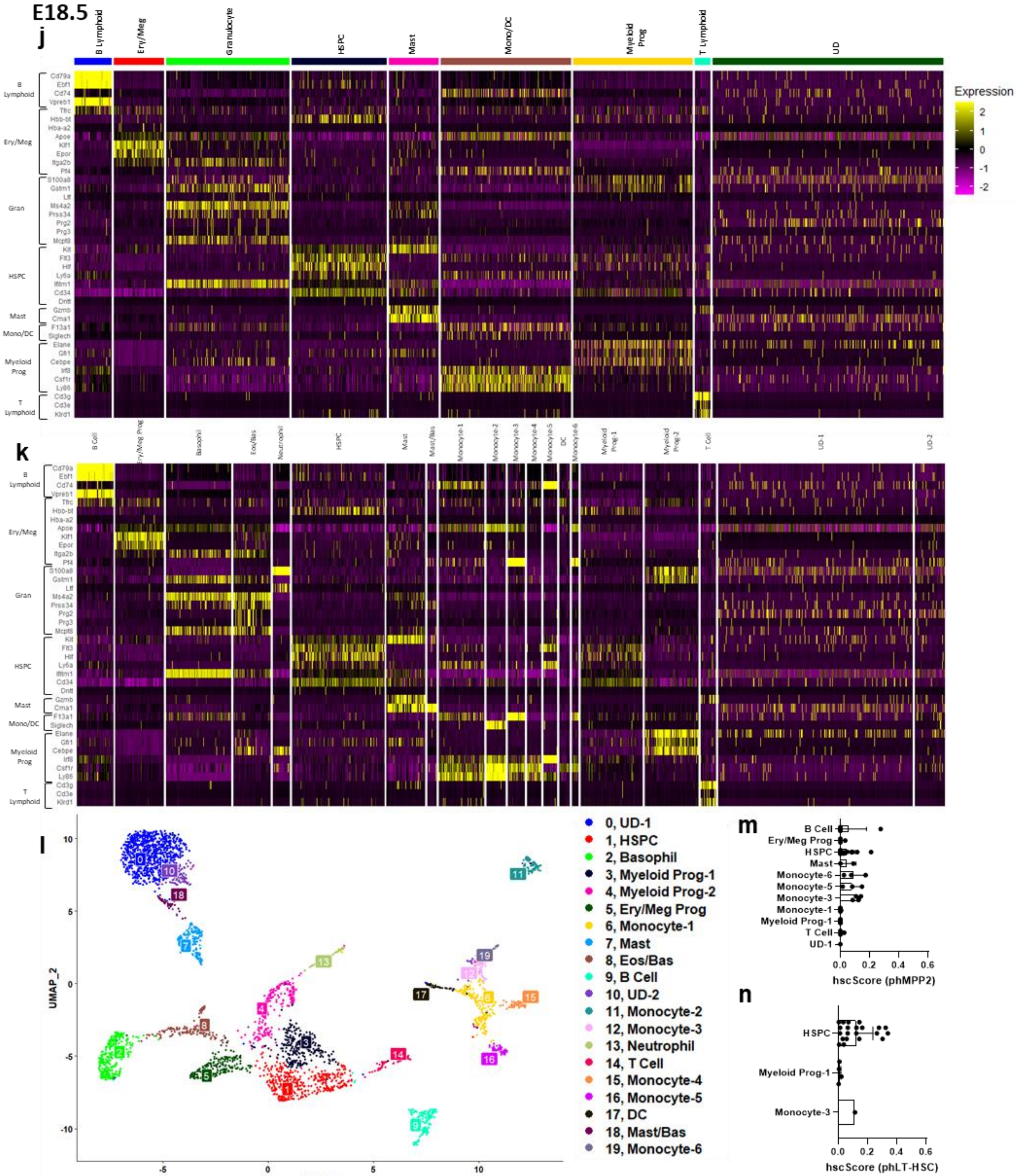

E18.5
j
B Lymphoid
Ery/Meg
Granulocyte
HSPC
Mast
Mono/DC
Myeloid Prog
T Lymphoid
UD
Expression
2
1
0
-1
-2
Gran
k
B Cell
Ery/Meg Prog
Basophil
Eos/Bas
Neutrophil
Mast/Bas
Monocyte-1
Monocyte-2
Monocyte-3
Monocyte-4
Monocyte-5
DC
Monocyte-6
Myeloid Prog-1
Myeloid Prog-2
T Cell
UD-1
UD-2
l
UMAP_1
UMAP_2
0, UD-1
1, HSPC
2, Basophil
3, Myeloid Prog-1
4, Myeloid Prog-2
5, Ery/Meg Prog
6, Monocyte-1
7, Mast
8, Eos/Bas
9, B Cell
10, UD-2
11, Monocyte-2
12, Monocyte-3
13, Neutrophil
14, T Cell
15, Monocyte-4
16, Monocyte-5
17, DC
18, Mast/Bas
19, Monocyte-6
m
hscScore (phMPP2)
n
hscScore (phLT-HSC)

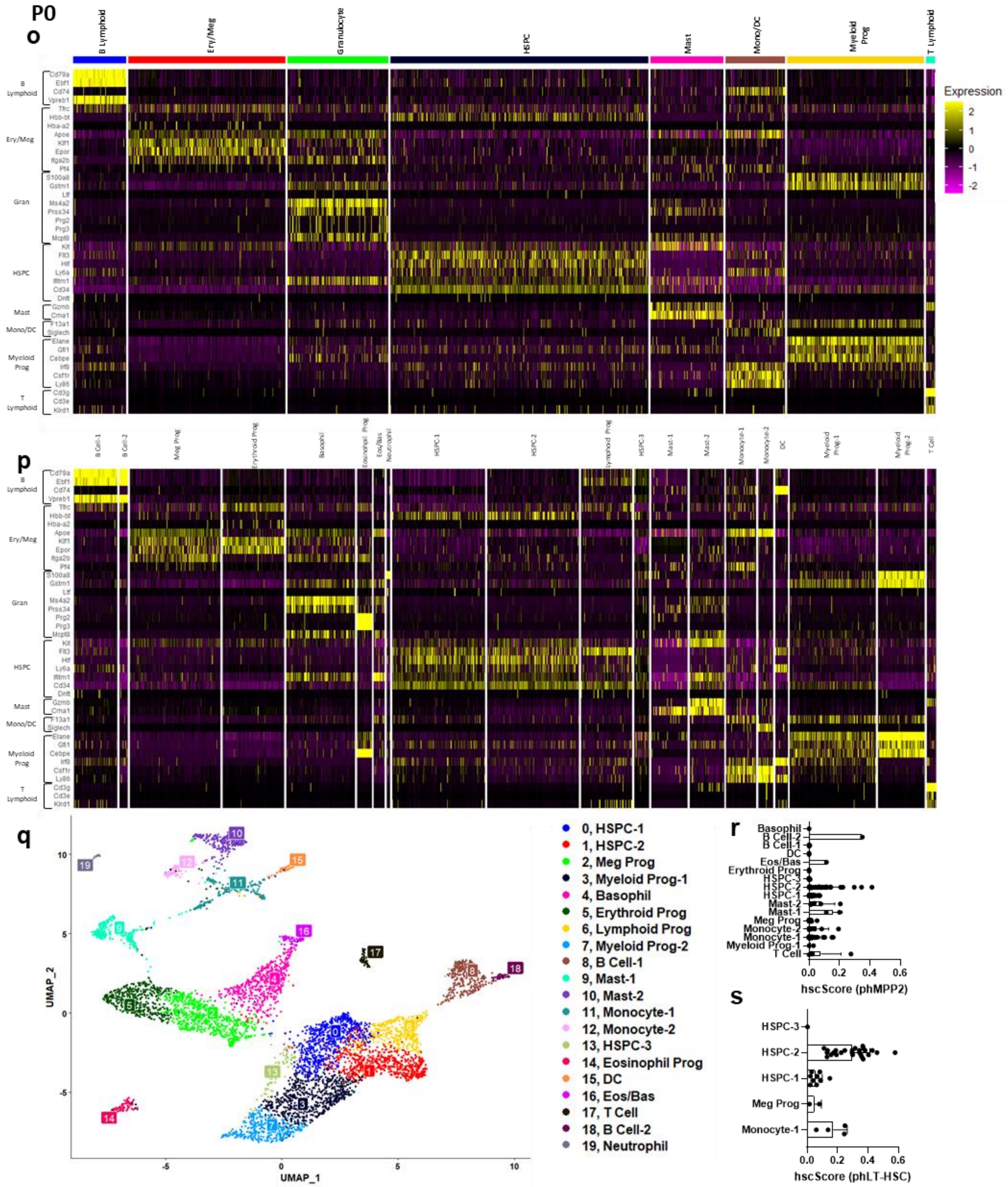

P0
o
B Lymphoid
Ery/Meg
Granulocyte
HSPC
Mast
Mono/DC
Myeloid Prog
T Lymphoid
Expression
2
1
0
-1
-2
Cd79a
Ebf1
Cd74
Vpreb1
Tfrc
Hbb-bt
Hba-a2
Apoe
Klf1
Epor
Itga2b
Pf4
S100a8
Gstm1
Ltf
Ms4a2
Prss34
Prg2
Prg3
Mcpt8
Kit
Flt3
Hlf
Ly6a
Ifitm1
Cd34
Dntt
Gzmb
Cma1
F13a1
Siglech
Elane
Gfi1
Cebpe
Irf8
Csf1r
Ly86
Cd3g
Cd3e
Klrd1
Gran
p
B Cell-1
B Cell-2
Meg Prog
Erythroid Prog
Basophil
Eosinophil Prog
Eos/Bas
Neutrophil
HSPC-1
HSPC-2
Lymphoid Prog
HSPC-3
Mast-1
Mast-2
Monocyte-1
Monocyte-2
DC
Myeloid Prog-1
Myeloid Prog-2
T Cell
q
UMAP_1
UMAP_2
0, HSPC-1
1, HSPC-2
2, Meg Prog
3, Myeloid Prog-1
4, Basophil
5, Erythroid Prog
6, Lymphoid Prog
7, Myeloid Prog-2
8, B Cell-1
9, Mast-1
10, Mast-2
11, Monocyte-1
12, Monocyte-2
13, HSPC-3
14, Eosinophil Prog
15, DC
16, Eos/Bas
17, T Cell
18, B Cell-2
19, Neutrophil
r
Basophil
B Cell-2
B Cell-1
DC
Eos/Bas
Erythroid Prog
HSPC-3
HSPC-2
HSPC-1
Mast-2
Mast-1
Meg Prog
Monocyte-2
Monocyte-1
Myeloid Prog-1
T Cell
0.0
0.2
0.4
0.6
hscScore (phMPP2)
s
HSPC-3
HSPC-2
HSPC-1
Meg Prog
Monocyte-1
hscScore (phLT-HSC)

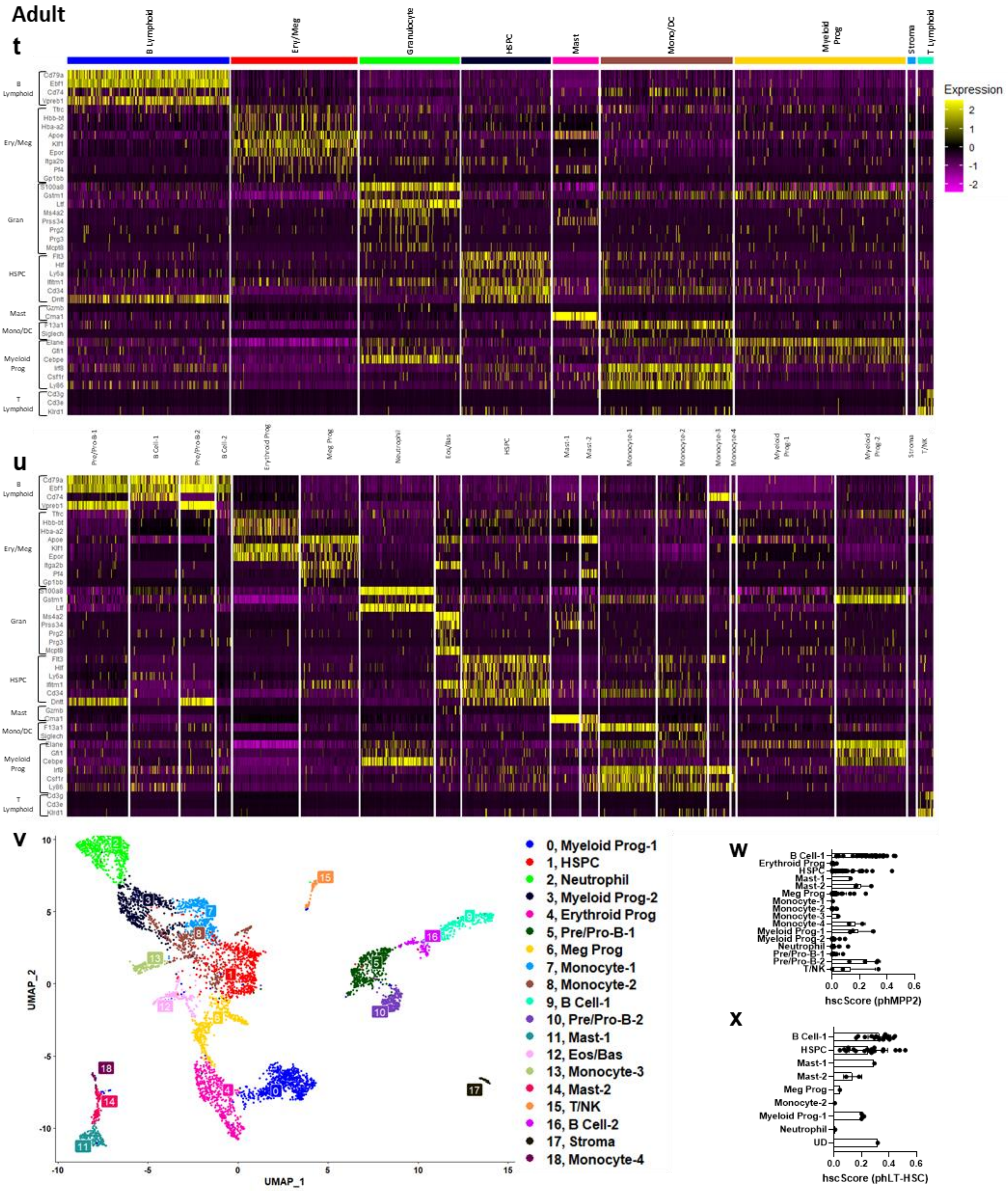

Adult
t
B Lymphoid
Ery/Meg
Granulocyte
HSPC
Mast
Mono/DC
Myeloid Prog
Stroma
T Lymphoid
Expression
2
1
0
-1
-2
Cd79a
Ebf1
Cd74
Vpreb1
Tfrc
Hbb-bt
Hba-a2
Apoe
Klf1
Epor
Itga2b
Pf4
Gp1bb
S100a8
Gstm1
Ltf
Ms4a2
Prss34
Prg2
Prg3
Mcpt8
Flt3
Hlf
Ly6a
Ifitm1
Cd34
Dntt
Gzmb
Cma1
F13a1
Siglech
Elane
Gfi1
Cebpe
Irf8
Csf1r
Ly86
Cd3g
Cd3e
Klrd1
Gran
Myeloid Prog
T Lymphoid
u
Pre/Pro-B-1
B Cell-1
Pre/Pro-B-2
B Cell-2
Erythroid Prog
Meg Prog
Neutrophil
Eos/Bas
HSPC
Mast-1
Mast-2
Monocyte-1
Monocyte-2
Monocyte-3
Monocyte-4
Myeloid Prog-1
Myeloid Prog-2
Stroma
T/NK
v
UMAP_2
UMAP_1
0, Myeloid Prog-1
1, HSPC
2, Neutrophil
3, Myeloid Prog-2
4, Erythroid Prog
5, Pre/Pro-B-1
6, Meg Prog
7, Monocyte-1
8, Monocyte-2
9, B Cell-1
10, Pre/Pro-B-2
11, Mast-1
12, Eos/Bas
13, Monocyte-3
14, Mast-2
15, T/NK
16, B Cell-2
17, Stroma
18, Monocyte-4
w
hscScore (phMPP2)
x
UD
hscScore (phLT-HSC)

Supplementary Figure 4 (related to Fig. 4). Expression of CITE-Seq and lineage markers for hematopoietic populations. a-d. Detection of CITE-Seq antibodies with corresponding mRNA expression across development. e. Heatmap of hematopoietic marker genes for broad populations in E16.5 (e), E18.5 (j), P0 (o), and Adult (t) BM HPs. Heatmap of hematopoietic marker genes in Seurat-defined subclusters in E16.5 (f), E18.5 (k), P0 (p), and Adult (u) FBM HPs. UMAP of Seurat-defined subclusters in E16.5 (g), E18.5 (l), P0 (q), and Adult (v) HPs. hscScore among hematopoietic cells within phMPP2s at E16.5 (h) (n =1-45 single cells), E18.5 (m) (n = 1-16 single cells), P0 (r) (n = 1-30 single cells), and Adult (w) (n = 1-40 single cells). hscScore among hematopoietic cells within phLT-HSCs at E16.5 (i) (n = 1-16 single cells), E18.5 (n) (1-21 single cells), P0 (s) (1-26 single cells), and Adult (x) (1-19 single cells). HSPC, hematopoietic stem and progenitors; Ery/Meg, erythroid/megakaryocyte; Gran, granulocyte; Mono/DC, monocyte/dendritic cell; Myeloid Prog, myeloid progenitors; UD, undefined; Eos/Bas, eosinophil/basophil; phMPP2; immunophenotypic multipotent progenitor-2; phLT-HSC, immunophenotypic long-term hematopoietic stem cell. Source data are provided in the Source Data File.

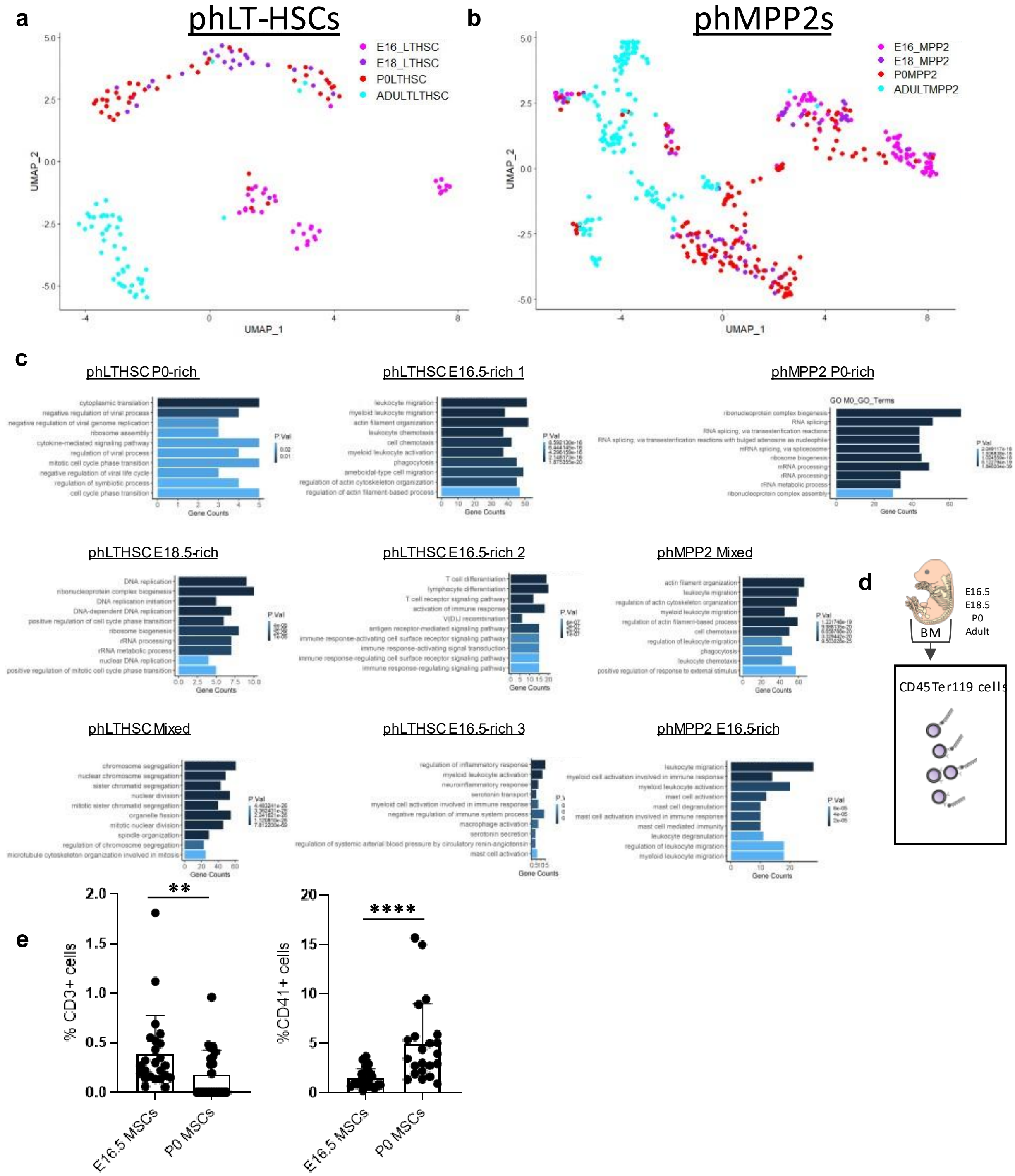
a
phLT-HSCs
E16_LTHSC
E18_LTHSC
P0LTHSC
ADULTLTHSC
UMAP_1
UMAP_2
b
phMPP2s
E16_MPP2
E18_MPP2
P0MPP2
ADULTMPP2
c
phLTHSC P0-rich
phLTHSC E16.5-rich 1
phMPP2 P0-rich
phLTHSC E18.5-rich
phLTHSC E16.5-rich 2
phMPP2 Mixed
phLTHSC Mixed
phLTHSC E16.5-rich 3
phMPP2 E16.5-rich
Gene Counts
P.Val
d
E16.5
E18.5
P0
Adult
BM
CD45-Ter119- cells
e
% CD3+ cells
%CD41+ cells
E16.5 MSCs
P0 MSCs
**
****

Supplementary Figure 5 (related to Fig. 5 and 6g). Clustering of CITE-Seq derived phLT-HSCs and phMPP2s across develop, GO terms associated with Fig. 5c and FL LT-HSC/MSC co-culture. Proportion of (a) phLT-HSCs or (b) phMPP2s at each developmental time-point contributing to each cluster (from Fig. 5a-b). c. GO terms associated with each respective cluster (from Fig. 5a-b). d. Schematic of scRNA-seq of stroma of perinatal and adult BM. e. CD3+ (left, $n = 24$, $P = 0.0047$) and CD41+ (right, $n = 22$-24, $P < 0.0001$) cell output of E16.5 FL LT-HSCs after co-culture with fetal or neonatal BM MSCs. Data are presented as means ± SD. **, $P < 0.01$; ****, $P < 0.0001$. P-Values determined by Mann-WhitneyTest, two-tailed. P-Values for GO terms determined by Bonferroni correction. GO, gene ontology; LT-HSC, long-term hematopoietic stem cell; MPP2, multipotent progenitor-2; phLT-HSC, immunophenotypic LT-HSC; phMPP2, immunophenotypic MPP2; BM, bone marrow; MSC, mesenchymal stroma cells. Source data are provided in the Source Data File.

E16.5

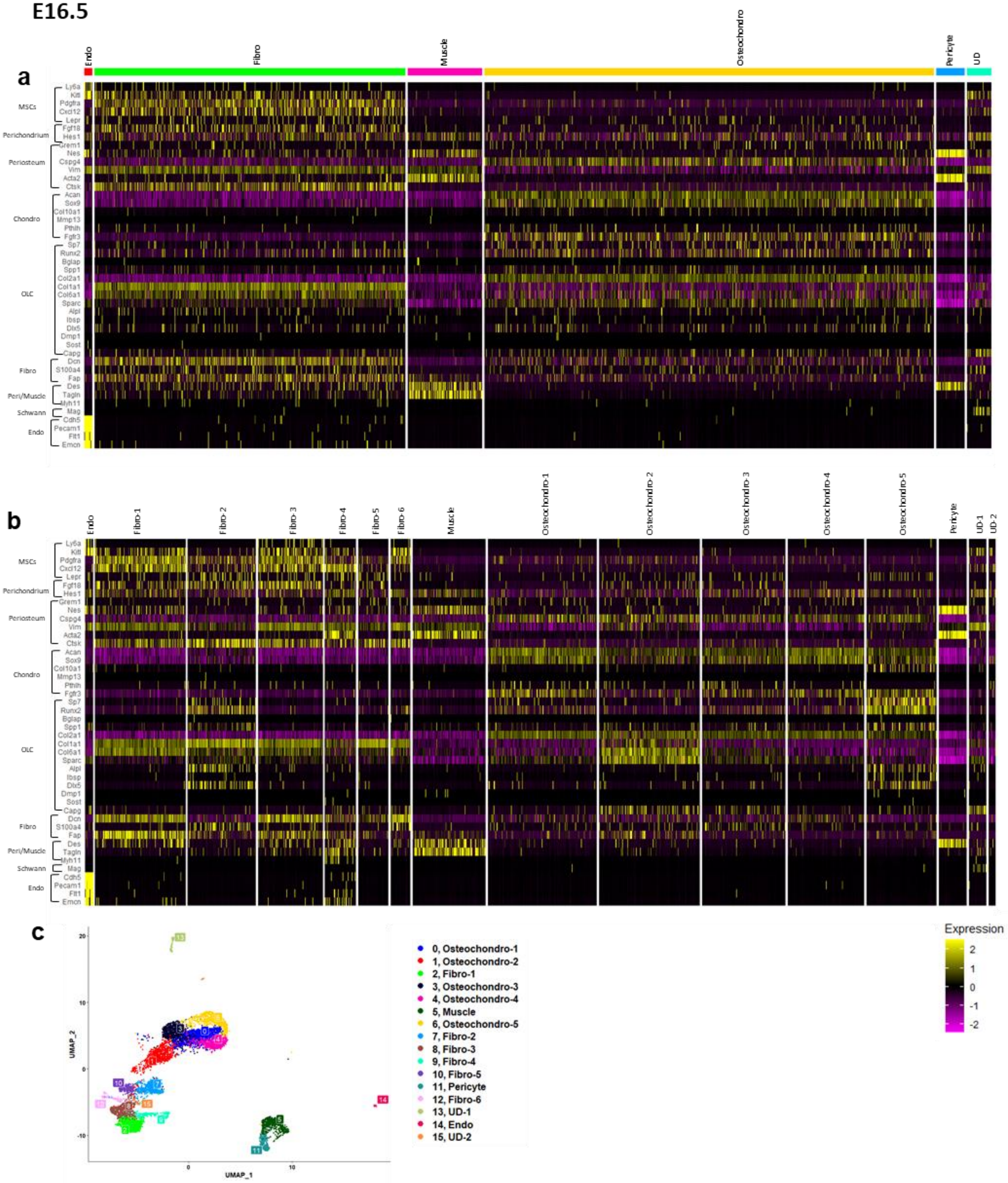
a
Endo
Fibro
Muscle
Osteochondro
Pericyte
UD
MSCs
Perichondrium
Periosteum
Chondro
OLC
Fibro
Peri/Muscle
Schwann
Endo
Ly6a
Kitl
Pdgfra
Cxcl12
Lepr
Fgf18
Hes1
Grem1
Nes
Cspg4
Vim
Acta2
Ctsk
Acan
Sox9
Col10a1
Mmp13
Pthlh
Fgfr3
Sp7
Runx2
Bglap
Spp1
Col2a1
Col1a1
Col6a1
Sparc
Alpl
Ibsp
Dlx5
Dmp1
Sost
Capg
Dcn
S100a4
Fap
Des
Tagln
Myh11
Mag
Cdh5
Pecam1
Flt1
Emcn
b
Endo
Fibro-1
Fibro-2
Fibro-3
Fibro-4
Fibro-5
Fibro-6
Muscle
Osteochondro-1
Osteochondro-2
Osteochondro-3
Osteochondro-4
Osteochondro-5
Pericyte
UD-1
UD-2
c
UMAP_1
UMAP_2
0, Osteochondro-1
1, Osteochondro-2
2, Fibro-1
3, Osteochondro-3
4, Osteochondro-4
5, Muscle
6, Osteochondro-5
7, Fibro-2
8, Fibro-3
9, Fibro-4
10, Fibro-5
11, Pericyte
12, Fibro-6
13, UD-1
14, Endo
15, UD-2
Expression
2
1
0
-1
-2

E18.5

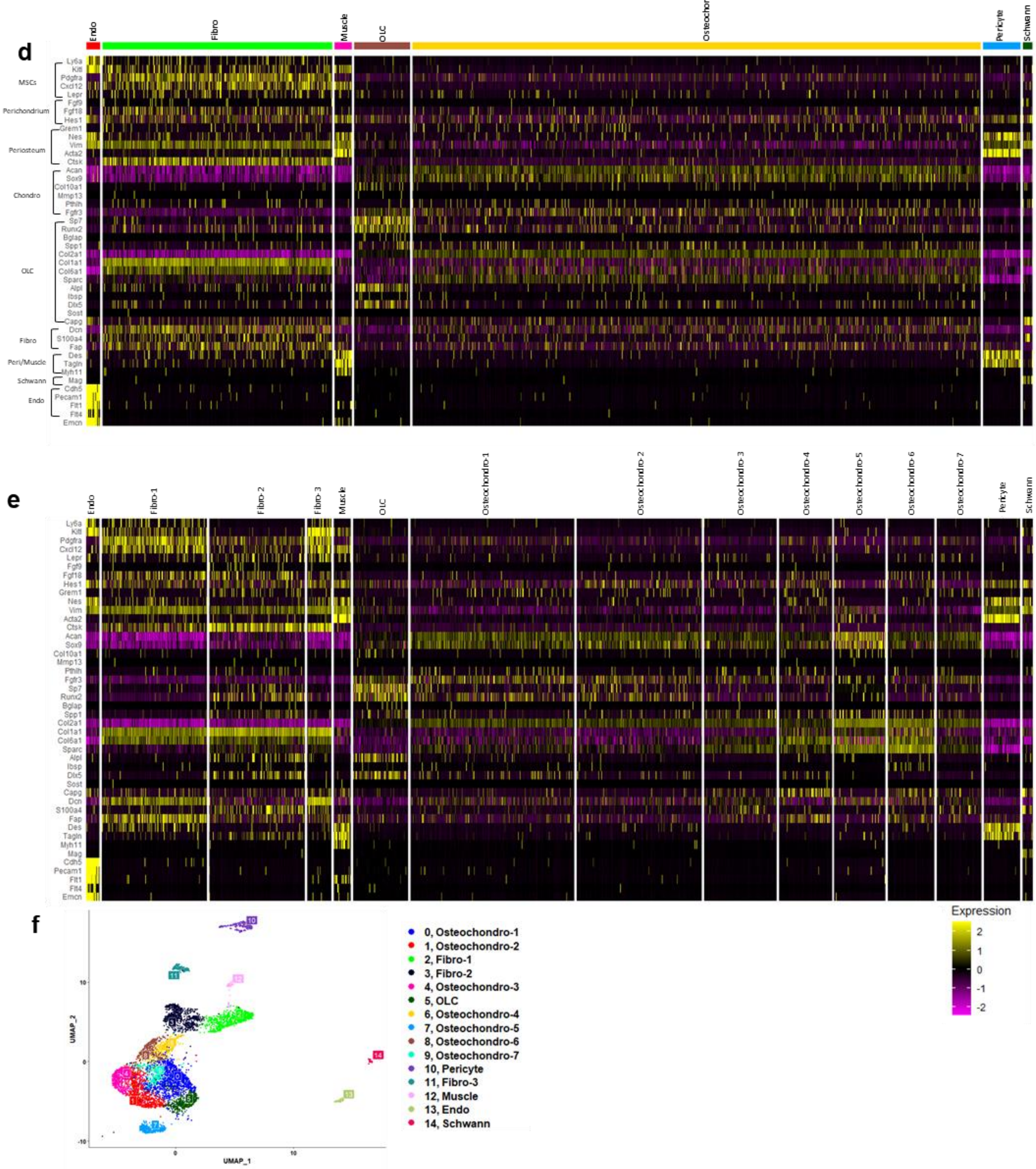
d
Endo
Fibro
Muscle
OLC
Osteochondro
Pericyte
Schwann
MSCs
Perichondrium
Periosteum
Chondro
OLC
Fibro
Peri/Muscle
Schwann
Endo
Ly6a
Kitl
Pdgfra
Cxcl12
Lepr
Fgf9
Fgf18
Hes1
Grem1
Nes
Vim
Acta2
Ctsk
Acan
Sox9
Col10a1
Mmp13
Pthlh
Fgfr3
Sp7
Runx2
Bglap
Spp1
Col2a1
Col1a1
Col6a1
Sparc
Alpl
Ibsp
Dlx5
Sost
Capg
Dcn
S100a4
Fap
Des
Tagln
Myh11
Mag
Cdh5
Pecam1
Flt1
Flt4
Emcn
e
Endo
Fibro-1
Fibro-2
Fibro-3
Muscle
OLC
Osteochondro-1
Osteochondro-2
Osteochondro-3
Osteochondro-4
Osteochondro-5
Osteochondro-6
Osteochondro-7
Pericyte
Schwann
f
UMAP_2
UMAP_1
0, Osteochondro-1
1, Osteochondro-2
2, Fibro-1
3, Fibro-2
4, Osteochondro-3
5, OLC
6, Osteochondro-4
7, Osteochondro-5
8, Osteochondro-6
9, Osteochondro-7
10, Pericyte
11, Fibro-3
12, Muscle
13, Endo
14, Schwann
Expression
2
1
0
-1
-2

P0

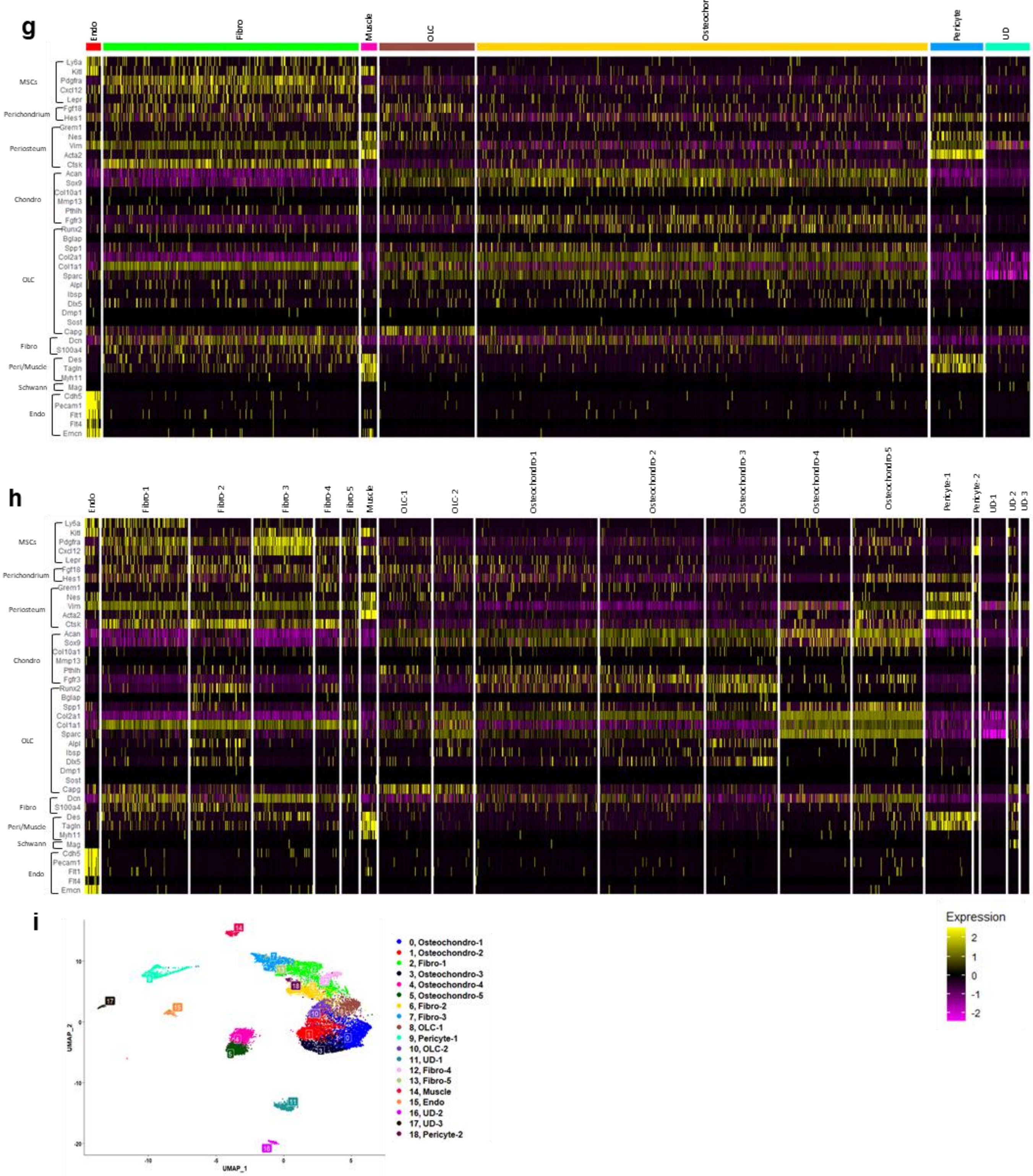
g
Endo
Fibro
Muscle
OLC
Osteochondro
Pericyte
UD
MSCs
Ly6a
Kitl
Pdgfra
Cxcl12
Lepr
Perichondrium
Fgf18
Hes1
Periosteum
Grem1
Nes
Vim
Acta2
Ctsk
Chondro
Acan
Sox9
Col10a1
Mmp13
Pthlh
Fgfr3
OLC
Runx2
Bglap
Spp1
Col2a1
Col1a1
Sparc
Alpl
Ibsp
Dlx5
Dmp1
Sost
Capg
Fibro
Dcn
S100a4
Peri/Muscle
Des
Tagln
Myh11
Schwann
Mag
Endo
Cdh5
Pecam1
Flt1
Flt4
Emcn
h
Endo
Fibro-1
Fibro-2
Fibro-3
Fibro-4
Fibro-5
Muscle
OLC-1
OLC-2
Osteochondro-1
Osteochondro-2
Osteochondro-3
Osteochondro-4
Osteochondro-5
Pericyte-1
Pericyte-2
UD-1
UD-2
UD-3
i
UMAP_2
UMAP_1
0, Osteochondro-1
1, Osteochondro-2
2, Fibro-1
3, Osteochondro-3
4, Osteochondro-4
5, Osteochondro-5
6, Fibro-2
7, Fibro-3
8, OLC-1
9, Pericyte-1
10, OLC-2
11, UD-1
12, Fibro-4
13, Fibro-5
14, Muscle
15, Endo
16, UD-2
17, UD-3
18, Pericyte-2
Expression
2
1
0
-1
-2

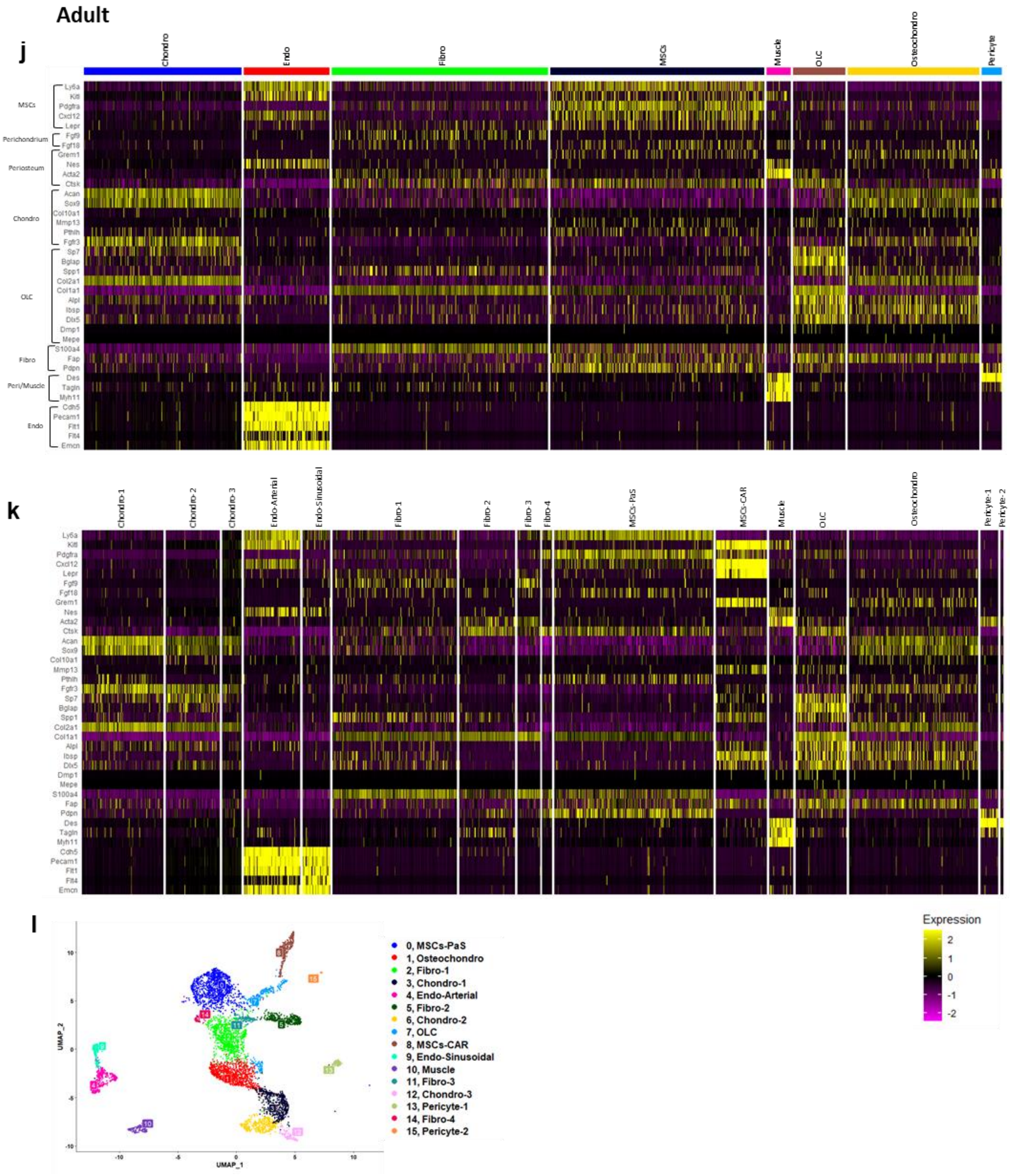
Adult
j
Chondro
Endo
Fibro
MSCs
Muscle
OLC
Osteochondro
Pericyte
MSCs
Perichondrium
Periosteum
Chondro
OLC
Fibro
Peri/Muscle
Endo
Ly6a
Kitl
Pdgfra
Cxcl12
Lepr
Fgf9
Fgf18
Grem1
Nes
Acta2
Ctsk
Acan
Sox9
Col10a1
Mmp13
Pthlh
Fgfr3
Sp7
Bglap
Spp1
Col2a1
Col1a1
Alpl
Ibsp
Dlx5
Dmp1
Mepe
S100a4
Fap
Pdpn
Des
Tagln
Myh11
Cdh5
Pecam1
Flt1
Flt4
Emcn
k
Chondro-1
Chondro-2
Chondro-3
Endo-Arterial
Endo-Sinusoidal
Fibro-1
Fibro-2
Fibro-3
Fibro-4
MSCs-PaS
MSCs-CAR
Muscle
OLC
Osteochondro
Pericyte-1
Pericyte-2
l
UMAP_1
UMAP_2
0, MSCs-PaS
1, Osteochondro
2, Fibro-1
3, Chondro-1
4, Endo-Arterial
5, Fibro-2
6, Chondro-2
7, OLC
8, MSCs-CAR
9, Endo-Sinusoidal
10, Muscle
11, Fibro-3
12, Chondro-3
13, Pericyte-1
14, Fibro-4
15, Pericyte-2
Expression
2
1
0
-1
-2

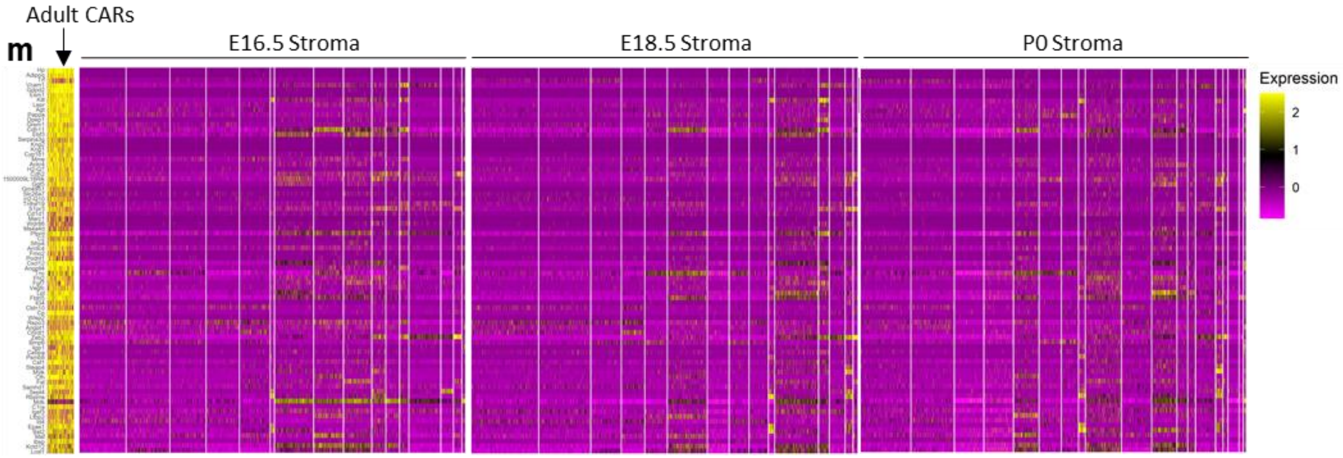

m
Adult CARs
E16.5 Stroma
E18.5 Stroma
P0 Stroma
Expression
2
1
0

Supplementary Figure 6 (Related to Fig. 7). Expression of lineage markers for BM stroma populations and the lack of a defined CAR cell population in the perinatal BM. Heatmap of stoma marker genes for broad populations in E16.5 (a), E18.5 (d), P0 (g), and Adult (j) BM stroma. Heatmap of stroma marker genes in Seurat-defined subclusters in E16.5 (b), E18.5 (e), P0 (h), and Adult (k) FBM stroma. UMAP of Seurat-defined subclusters in E16.5 (c), E18.5 (f), P0 (i), and Adult (l) HPs. m. Expression of an adult CAR signature within different cell types across development. Endo, endothelial; Fibro, fibroblast; Osteochondro, osteochondroprogenitor; UD, undefinded; CAR, CXCL12-abundant reticular cells; MSCs, mesenchymal stem cells; MSCs-PαS, MSCs-Pdgfra^{+}Sca1^{+} cells; Chondro, chondrocyte; OLC, osteolineage cells; Peri, pericytes; BM, bone marrow.

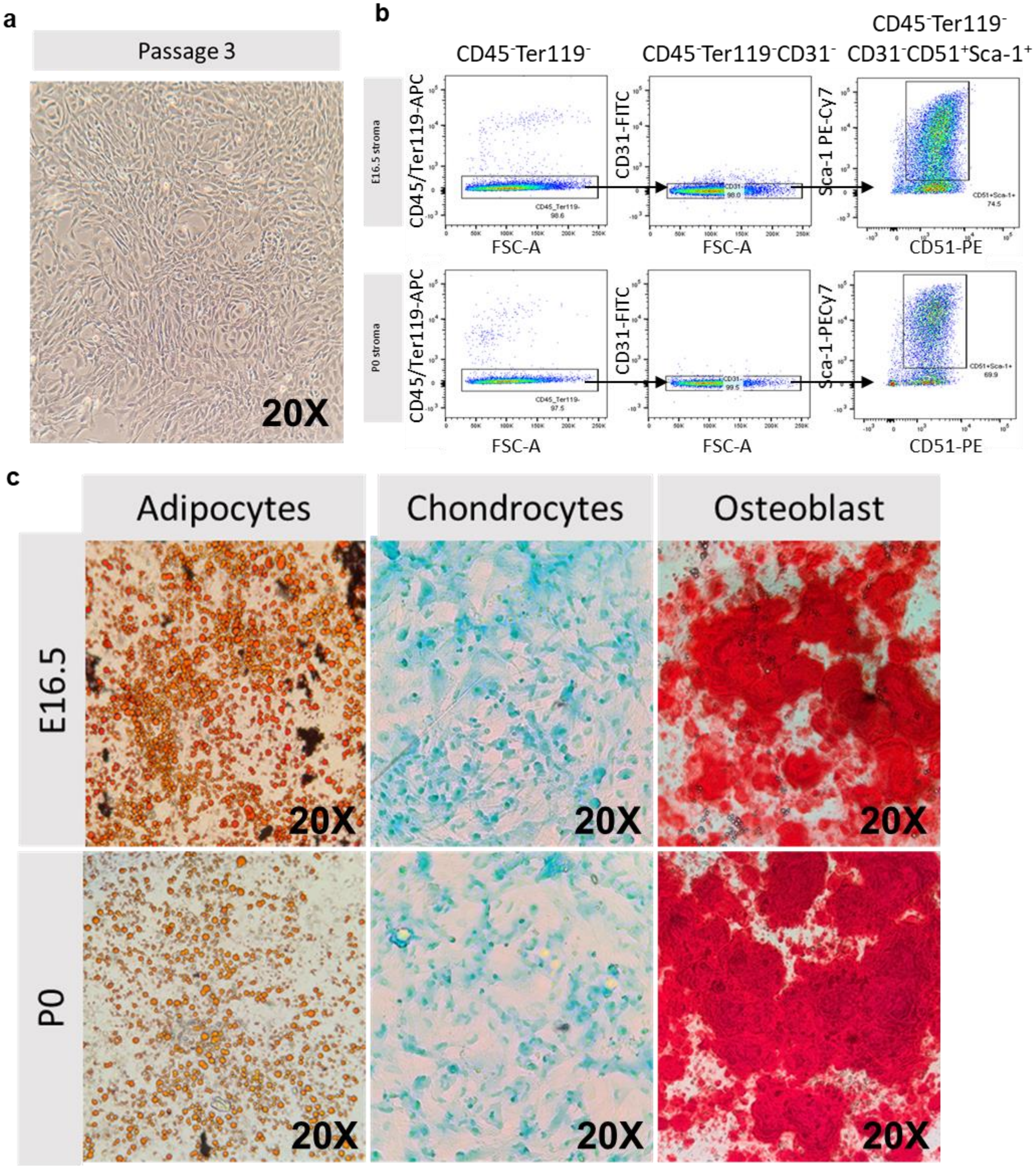
a
Passage 3
20X
b
CD45⁻Ter119⁻
CD45⁻Ter119⁻CD31⁻
CD45⁻Ter119⁻ CD31⁻CD51⁺Sca-1⁺
E16.5 stroma
P0 stroma
CD45/Ter119-APC
CD31-FITC
Sca-1 PE-Cy7
Sca-1-PECy7
FSC-A
CD51-PE
c
Adipocytes
Chondrocytes
Osteoblast
E16.5
P0
20X

Supplementary Figure 7 (Related to Fig. 7). E16.5 and P0 FBM stroma co-culture. a. Representative images of expanded E16.5 and P0 $CD45^{-}Ter119^{-}$ bone marrow cultures from 3 independent experiments. b. Representative flow plots showing an enrichment for MSCs in expanded E16.5 and P0 stroma cultures from 5 independent experiments. c. Representative images of the differentiation potential of expanded E16.5 and P0 $CD45^{-}Ter119^{-}$ bone marrow expanded cultures (passage 2-3) from 3 independent experiments. All images are at 20X magnification. FBM, fetal bone marrow.

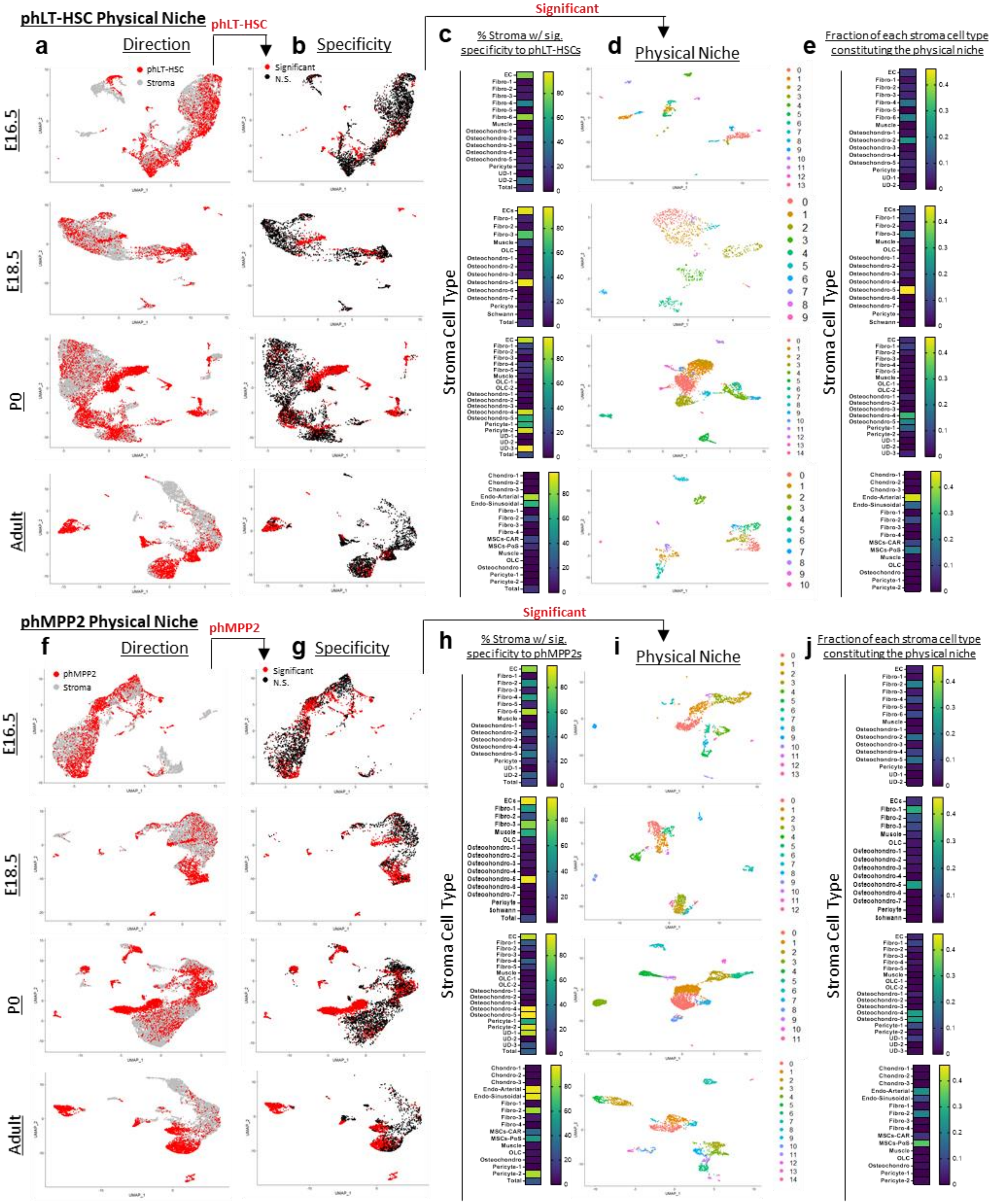
phLT-HSC Physical Niche
phLT-HSC
Significant
a
Direction
b
Specificity
c
% Stroma w/ sig. specificity to phLT-HSCs
d
Physical Niche
e
Fraction of each stroma cell type constituting the physical niche
phLT-HSC
Stroma
Significant
N.S.
E16.5
E18.5
P0
Adult
Stroma Cell Type
phMPP2 Physical Niche
phMPP2
Significant
f
Direction
g
Specificity
h
% Stroma w/ sig. specificity to phMPP2s
i
Physical Niche
j
Fraction of each stroma cell type constituting the physical niche
phMPP2
Stroma
Significant
N.S.
E16.5
E18.5
P0
Adult
Stroma Cell Type

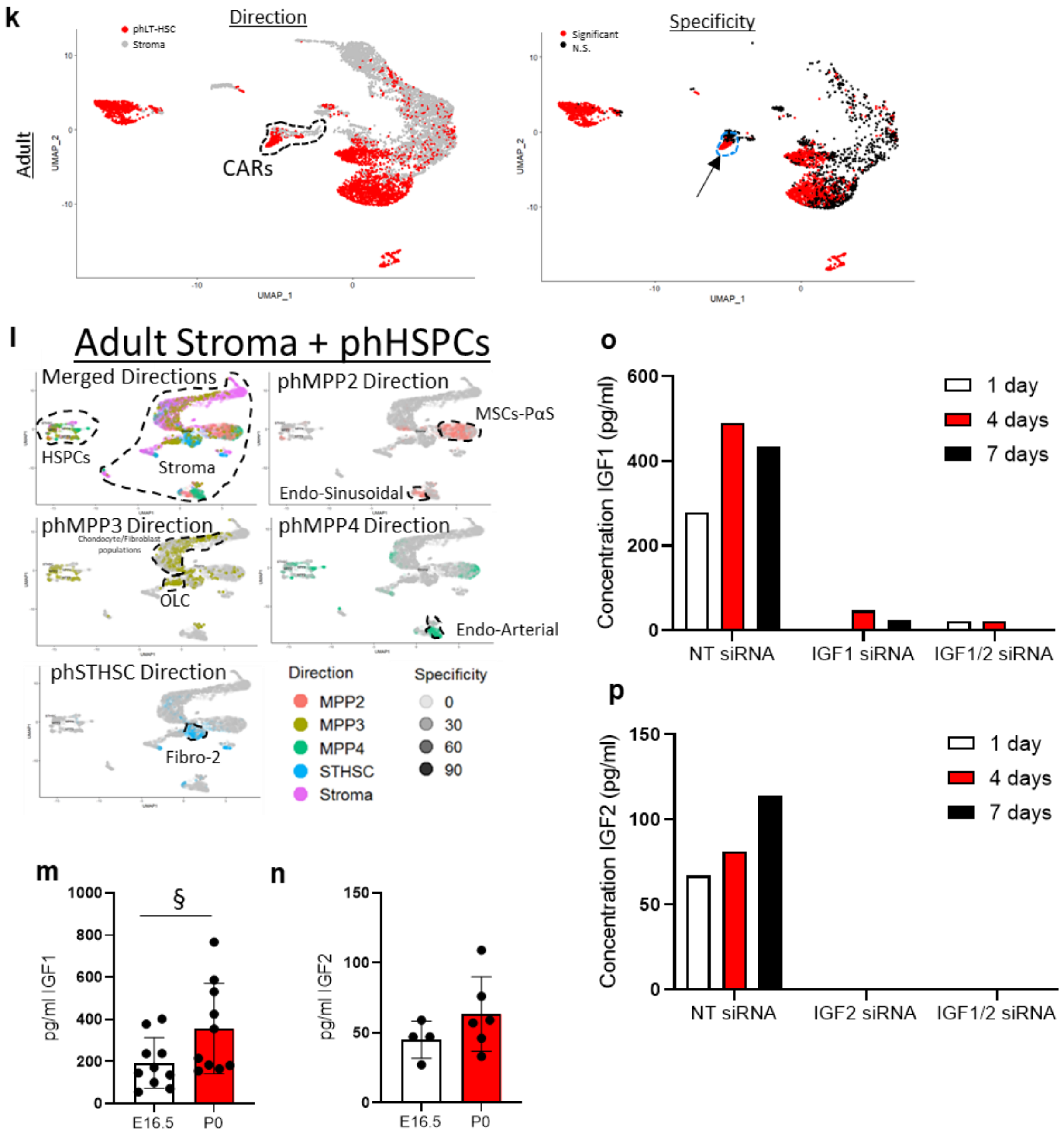
k
Direction
phLT-HSC
Stroma
Adult
UMAP_2
UMAP_1
CARs
Specificity
Significant
N.S.
l
Adult Stroma + phHSPCs
Merged Directions
HSPCs
Stroma
phMPP2 Direction
MSCs-PαS
Endo-Sinusoidal
phMPP3 Direction
Chondocyte/Fibroblast populations
OLC
phMPP4 Direction
Endo-Arterial
phSTHSC Direction
Fibro-2
Direction
MPP2
MPP3
MPP4
STHSC
Stroma
Specificity
0
30
60
90
m
pg/ml IGF1
§
E16.5
P0
n
pg/ml IGF2
E16.5
P0
o
Concentration IGF1 (pg/ml)
1 day
4 days
7 days
NT siRNA
IGF1 siRNA
IGF1/2 siRNA
p
Concentration IGF2 (pg/ml)
1 day
4 days
7 days
NT siRNA
IGF2 siRNA
IGF1/2 siRNA

Supplementary Figure 8 (Related to Fig. 8). Identification of the putative physical niches for E16.5, E18.5, and P0 perinatal BM. RNA-Magnet analysis for stroma cells constituting the phLT-HSC physical niche, including: (a) Direction of cells toward phLT-HSCs (red) or stroma (gray), (b) cells with direction toward phLT-HSCs with significant (red) or not significant (N.S., black) specificity scores, (c) the percentage of each stroma cell type with significant specificity toward phLT-HSCs, (d) clustering of all stroma cells (with phLT-HSCs) that show significant specificity toward phLT-HSCs, and (e) the stroma composition of the predicted niche. RNA-Magnet analysis for stroma cells constituting the phMPP2 physical niche, including: (f) Direction of cells toward phMPP2s (red) or stroma (gray), (g) cells with direction toward phMPP2s with significant (red) or not significant (N.S., black) specificity scores, (h) the percentage of each stroma cell type with significant specificity toward phMPP2s, (i) clustering of all stroma cells (with phMPP2s) that show significant specificity toward phMPP2s, and (j) the stroma composition of the niche. k. Direction and specificity of adult cells toward phLT-HSCs, highlighted the CAR population (encircled in dotted line). l. Direction and specificity of adult cells toward phHSPCs (phST-HSCs, phMPP2s, phMPP3s, phMPP4s). IGF1 (m) ($n = 10$, $P = 0.0524$) and IGF2 (n) ($n = 4\text{-}6$) secreted proteins levels on E16.5 and P0 MSCs supernatants. Data are presented as means ± SD. §, $P < 0.1$. IGF1 (o) and IGF2 (p) secreted proteins levels in BM-MSCs one, four and seven days post-treatment with IGF1, IGF2, or control siRNAs. P-Values determined by Mann-WhitneyTest, two-tailed. BM, bone marrow; HSPCs, hematopoietic stem and progenitor cells; LT-HSC, long-term hematopoietic stem cell; ST-HSC, short-term hematopoietic stem cell; MPP, multipotent progenitor; phLT-HSC, immunophenotypic LT-HSC, phST-HSC, immunophenotypic ST-HSC; phMPP2, immunophenotypic MPP2; phMPP3, immunophenotypic MPP3; phMPP4, immunophenotypic MPP4; EC, endothelial cells; Fibro, fibroblast; Osteochondro, osteochondroprogenitor; UD, undefined; OLC, osteolineage cell; Endo-Arterial, arterial endothelial cells; Endo-Sinusoidal, sinusoidal endothelial cells; MSCs, mesenchymal stem cells; CAR, CXCL12-abundant reticular cells; PαS, $Pdgfra^{+}Sca1^{+}$ cells; NT, non-targeting control. Source data are provided in the Source Data File.
