# Supplementary material for: Murine fetal bone marrow does not support functional hematopoietic stem and progenitor cells until birth
Source: Nat Commun. 2022 Sep 15;13:5403. doi: 10.1038/s41467-022-33092-4 (PMC9477881; doi:10.1038/s41467-022-33092-4)
Supplement: Supplementary file 3 — Description of Additional Supplementary Files [file 41467_2022_33092_MOESM3_ESM.pdf]

Description of Additional Supplementary Files

File Name: Supplementary Data 1
Description: Genes with myAUC >0.7 for all clusters in each HP and stroma library across development.

File Name: Supplementary Data 2
Description: GO Analyses.

File Name: Supplementary Data 3
Description: Mice, antibodies, Reagents and Kits, Oligonucleotides, Software, and Deposited Data information.
