# Supplementary material for: Murine fetal bone marrow does not support functional hematopoietic stem and progenitor cells until birth
Source: Nat Commun. 2022 Sep 15;13:5403. doi: 10.1038/s41467-022-33092-4 (PMC9477881; doi:10.1038/s41467-022-33092-4)
Supplement: Supplementary file 7 — Reporting Summary [file 41467_2022_33092_MOESM7_ESM.pdf]

nature portfolio

Corresponding author(s): Shannon McKinney-Freeman

Last updated by author(s): Aug 26, 2022

Reporting Summary

Nature Portfolio wishes to improve the reproducibility of the work that we publish. This form provides structure for consistency and transparency in reporting. For further information on Nature Portfolio policies, see our Editorial Policies and the Editorial Policy Checklist.

Statistics

For all statistical analyses, confirm that the following items are present in the figure legend, table legend, main text, or Methods section.

n/a	Confirmed	
☐	☒	The exact sample size (*n*) for each experimental group/condition, given as a discrete number and unit of measurement
☐	☒	A statement on whether measurements were taken from distinct samples or whether the same sample was measured repeatedly
☐	☒	The statistical test(s) used AND whether they are one- or two-sided<br>*Only common tests should be described solely by name; describe more complex techniques in the Methods section.*
☐	☒	A description of all covariates tested
☐	☒	A description of any assumptions or corrections, such as tests of normality and adjustment for multiple comparisons
☐	☒	A full description of the statistical parameters including central tendency (e.g. means) or other basic estimates (e.g. regression coefficient) AND variation (e.g. standard deviation) or associated estimates of uncertainty (e.g. confidence intervals)
☐	☒	For null hypothesis testing, the test statistic (e.g. *F*, *t*, *r*) with confidence intervals, effect sizes, degrees of freedom and *P* value noted<br>*Give P values as exact values whenever suitable.*
☒	☐	For Bayesian analysis, information on the choice of priors and Markov chain Monte Carlo settings
☒	☐	For hierarchical and complex designs, identification of the appropriate level for tests and full reporting of outcomes
☒	☐	Estimates of effect sizes (e.g. Cohen's *d*, Pearson's *r*), indicating how they were calculated

Our web collection on statistics for biologists contains articles on many of the points above.

Software and code

Policy information about availability of computer code

Data collection	BD FACSDiva Software Version 9.0 was used for all flow cytometry data collection
Data analysis	R Version 3.6.0/3.6.3/4.0.3<br>RStudio Version 1.3.1093<br>FlowJo v10<br>Seurat v3/4<br>CellRanger<br>GraphPad Prism Version 9<br>RNAMagnet Version 0.1.0<br>SingleCellNet Version 0.1.0<br>Slingshot Version 1.6.1<br>clusterProfiler 3.16.1<br>MAGIC Version 2.0.3<br><br>More detail is available in Supplementary Table 3

For manuscripts utilizing custom algorithms or software that are central to the research but not yet described in published literature, software must be made available to editors and reviewers. We strongly encourage code deposition in a community repository (e.g. GitHub). See the Nature Portfolio guidelines for submitting code & software for further information.

Data

Policy information about availability of data

All manuscripts must include a data availability statement. This statement should provide the following information, where applicable:

- Accession codes, unique identifiers, or web links for publicly available datasets
- A description of any restrictions on data availability
- For clinical datasets or third party data, please ensure that the statement adheres to our policy

The single-cell RNA sequencing datasets generated during this study have been deposited to GEO under accession number GSE178951. Other data analyzed in this manuscript: Li et al. 2020: GSE128761; Baccin et al. 2020: GSE122467. Accession numbers are listed in Supplementary Data 3.

Human research participants

Policy information about studies involving human research participants and Sex and Gender in Research.

Reporting on sex and gender	Not applicable
Population characteristics	Not applicable
Recruitment	Not applicable
Ethics oversight	Not applicable

Note that full information on the approval of the study protocol must also be provided in the manuscript.

Field-specific reporting

Please select the one below that is the best fit for your research. If you are not sure, read the appropriate sections before making your selection.

☒ Life sciences ☐ Behavioural & social sciences ☐ Ecological, evolutionary & environmental sciences

For a reference copy of the document with all sections, see nature.com/documents/nr-reporting-summary-flat.pdf

Life sciences study design

All studies must disclose on these points even when the disclosure is negative.

Sample size	Sample sizes were determined based on previous experience and traditional sample size, but no sample size calculation was performed. For instance, for transplants we attempted to include an n = 5 for each group and for all experiments we attempted to include an n >= 3. For flow cytometry analysis of bone marrow and fetal liver hematopioetic progenitor populations, we always included n >= 5 embryos/pups across multiple litters.
Data exclusions	No data was excluded from the analyses
Replication	Figure 1b: 9-18 primary recipients from pools of E15.5, E16.5, E17.5, E18.5, P0, and adult donors. Also Supplementary Figure 1f<br>Figure 1c: 8-19 primary recipients at 20 weeks post-transplant<br>Figure 1d: 3 independent secondary transplants consisting of 5 recipients/transplant. Also Supplementary Figure 1g-h<br>Figure 2a-d: 10-17 individual embryos from 2-3 independent litters. Also Supplementary Figures 2c-d, 3a-b<br>Figure 2e: n = 2-3, cells pooled from multiple embryos<br>Figure 2f: n = 3 distinct experiments, with each experiment consisting of pools of cells from embryos from distinct litters<br>Figure 3b: 87-171 single cells from 3 experiments, each experiment consisting of cells taken from a pool of cells combined from a distinct litter of embryos<br>Figure 3c: 111-175 single cells from 3 experiments, each experiment consisting of cells taken from a pool of cells combined from a distinct litter of embryos<br>Figure 3e: 9-10 primary recipients from pools of MPP2s from E16.5, E18.5, P0, and adult donors. Also Supplementary Figure 3e.<br>Figure 3f: 5-9 primary recipients from pools of MPP2s from E16.5, E18.5, P0, and adult donors. Also Supplementary Figure 3e.<br>Figure 3g-h: 9-14 primary recipients from pools of LT-HSCs from E16.5, E18.5, P0, and adult donors at 16 weeks post-transplant<br>Figure 4f: For phMPP2s: n = 85 (E16.5), 55 (E18.5), 141 (P0), 147 (Adult); For phLT-HSCs: n = 33 (E16.5), 26 (E18.5), 43 (P0), 46 (Adult)<br>Figure 5d: n = 148 phLT-HSCs<br>Figure 5g: n = 428 phMPP2s<br>Figure 6g: n = 12 (No Feeder), n = 24 (Feeder), with each replicate representing a pool of E16.5 fetal liver LT-HSCs from multiple embryos<br>Figure 7d-f: n = 9-12, with each replication representing a pool of E16.5 or P0 fetal liver LT-HSCs from multiple embryos<br>Figure 7g-h: n = 6, with each replication representing a pool of E16.5 or P0 fetal liver LT-HSCs from multiple embryos<br>Figure 8g: n = 6, with each replication representing a pool of P0 LT-HSCs from multiple embryos<br>Figure 8h: n = 3, with each replication representing a pool of P0 LT-HSCs from multiple embryos<br>Supplementary Figure 1b: n = 10-17 embryos from 3 independent litters

Supplementary Figure 2e-f: n = 3, with each replicate consisting of a pool of HSPCs from multiple embryos
Supplementary Figure 5e: n = 22-24, with each replicate consisting of a pool of E16.5 fetal liver LT-HSCs from multiple embryos
Supplementary Figure 8m: n = 10
Supplementary Figure 8n: n = 4-6
Supplementary Figure 8o-p: Data represent 1 experiment, repeated with 3 independent MSC lines

All replicates are included in the data presented in this manuscript. Due to the scope of the scRNA-Seq experiments, they were not repeated. Instead, for each scRNA-Seq dataset (E16.5, E18.5, P0, adult hematopoietic and stroma cells), multiple mice were pooled together to get sufficient representation of the cell types present.

Randomization

There was no randomization. Embryos and neonates were assigned to groups based on age determined by standard methods (e.g. timed pregnancies). Animals were analyzed based on their assigned group, and randomization was not needed as there were no treatment cohorts. Covariates were controlled by using age matched animals (when fetal/neonatal animals were used) or age and sex-matched animals (when adult animals were used).

Blinding

Although no specific methods were used for blinding, blood samples were typically collected from mice by one individual, data collected by flow cytometry by a different individual, and then data analyzed by a different individual, at which time the assigned group (e.g. for transplants the recipient group) for each particular mouse analyzed was not known. All statistics were performed by an independent biostatistics expert who was unaware of the biological significance of distinct groups of mice (or samples) being examined.

Reporting for specific materials, systems and methods

We require information from authors about some types of materials, experimental systems and methods used in many studies. Here, indicate whether each material, system or method listed is relevant to your study. If you are not sure if a list item applies to your research, read the appropriate section before selecting a response.

Materials & experimental systems

n/a	Involved in the study	
☐	☒	Antibodies
☒	☐	Eukaryotic cell lines
☒	☐	Palaeontology and archaeology
☐	☒	Animals and other organisms
☒	☐	Clinical data
☒	☐	Dual use research of concern

Methods

n/a	Involved in the study	
☒	☐	ChIP-seq
☐	☒	Flow cytometry
☒	☐	MRI-based neuroimaging

Antibodies

Antibodies used

Antibody/Vendor/Clone/Catalog #/(Dilution)
B220-BV605 Biolegend RA3-6B2 103244 (1:400)
B220-PE-Cy7 Tonbo Biosciences RA3-6B2 60-0452-U100 (1:200)
B220-PerCP-Cy5.5 BioGems RA3-6B2 07131-70-100 (1:200)
CD117 Microbeads Miltenyi Biotec 130-091-224 (1:200)
CD117-APCe780 Invitrogen 2B8 47-1171-82 (1:200)
CD11b-PerCP-Cy5.5 Biolegend M1/70 101228 (1:200)
CD135-APC Thermo Fisher Scientific A2F10 135310 (1:50)
CD135-PE Biolegend A2F10 135306 (1:50)
CD135-TotalSeqA Biolegend A2F10 135316 (1:50)
CD150-PE-Cy7 Biolegend TC15-12F12.2 115914 (1:200)
CD150-TotalSeqA Biolegend TC15-12F12.2 115945 (1:200)
CD19-APC Biolegend 1D3 152409 (1:200)
CD31-FITC Biolegend 390 102405 (1:200)
CD32/16-A700 Invitrogen 93 56-0161-82 (1:200)
CD34-A647 BD Biosciences RAM34 560233 (1:50)
CD34-FITC BD Biosciences RAM34 553733 (1:50)
CD3-PE Biolegend 17A2 100206 (1:200)
CD41-BV605 Biolegend MWReg30 133921 (1:400)
CD41-PerCPe710 Invitrogen eBioMWReg30 (MWReg30) 46-0411-82 (1:200)
CD45.1-APC Invitrogen A20 17-0453-82 (1:200)
CD45.1-FITC Biolegend A20 110706 (1:200)
CD45.2-PE Biolegend 104 109807 (1:200)
CD45.2-v500 BD Biosciences 104 562129 (1:200)
CD45-APC Biolegend 30-F11 103112 (1:200)
CD45-FITC Biolegend S18009F 157214 (1:200)
CD45-PE Biolegend 30-F11 103106 (1:200)
CD48-A700 Biolegend HM48-1 103426 (1:200)
CD48-TotalSeqA Biolegend HM48-1 103447 (1:200)
CD4-BV605 Biolegend GK1.5 100451 (1:400)
CD4-PE-Cy7 Tonbo Biosciences GK1.5 60-0042-U100 (1:200)
CD51-PE Biolegend RMV-7 104105 (1:200)

CD8-BV605 Biolegend 53-6.7 100744 (1:400)
CD8-PE-Cy7 Tonbo Biosciences 53-6.7 60-0081-U100 (1:200)
cKIT-APCe780 Invitrogen 2B8 2261910 (1:200)
CXCR4-FITC BD Biosciences 2B11/CXCR4 551967 (1:200)
EPCR STEMCELL Technologies RMEPCR1560 (1560) 60038PE (1:200)
Gr-1-BV605 Biolegend RB6-8C5 108440 (1:400)
Gr-1-PerCP-Cy5.5 BD Biosciences RB6-8C5 552093 (1:200)
Sca-1 -PECy7 Biolegend D7 108113 (1:200)
Sca1-PerCP-Cy5.5 Biolegend D7 108123 (1:200)
Sca1-TotalSeqA Biolegend D7 108147 (1:200)
Ter119-APC Biolegend TER-119 116212 (1:200)
Ter119-BV605 Biolegend TER-119 116239 (1:400)
Ter119-PECy7 Biolegend TER-119 116222 (1:200)

Information on supplier name, catalog number, clone name, and dilution can also be found in Supplementary Data 3

Validation

Antibody/Validation
B220-BV605 Validated by manufacturer against C57BL/6 splenocytes
B220-PE-Cy7 Validated by manufacturer
B220-PerCP-Cy5.5 Validated by manufacturer
CD117 Microbeads Validated by manufacturer against bone marrow cells
CD117-APCe780 Validated by manufacturer against C57BL/6 bone marrow cells
CD11b-PerCP-Cy5.5 Validated by manufacturer against C57BL/6 splenocytes
CD135-APC Validated by manufacturer against C57BL/6 bone marrow cells
CD135-PE Validated by manufacturer against C57BL/6 bone marrow cells
CD135-TotalSeqA Validated by manufacturer
CD150-PE-Cy7 Validated by manufacturer against C57BL/6 mouse bone marrow cells
CD150-TotalSeqA Validated by manufacturer
CD19-APC Validated by manufacturer against C57BL/6 splenocytes
CD31-FITC Validated by manufacturer against C57BL/6 splenocytes
CD32/16-A700 Validated by manufacturer against C57BL/6 splenocytes
CD34-A647 Validated by manufacturer against BALB/c bone marrow cells
CD34-FITC Validated by manufacturer against BALB/c bone marrow cells
CD3-PE Validated by manufacturer against C57BL/6 splenocytes
CD41-BV605 Validated by manufacturer against C57BL/6 platelets
CD41-PerCPe710 Validated by manufacturer against mouse platelets
CD45.1-APC Validated by manufacturer against SJL mouse splenocytes
CD45.1-FITC Validated by manufacturer against SJL mouse splenocytes
CD45.2-PE Validated by manufacturer against BALB/c and SJL splenocytes
CD45.2-v500 Validated by manufacturer against BALB/c splenocytes
CD45-APC Validated by manufacturer against C57BL/6 splenocytes
CD45-FITC Validated by manufacturer against C57BL/6 splenocytes
CD45-PE Validated by manufacturer against C57BL/6 splenocytes
CD48-A700 Validated by manufacturer against C57BL/6 splenocytes
CD48-TotalSeqA Validated by manufacturer
CD4-BV605 Validated by manufacturer against C57BL/6 splenocytes
CD4-PE-Cy7 Validated by manufacturer
CD51-PE Validated by manufacturer against C57BL/6 bone marrow cells
CD8-BV605 Validated by manufacturer against C57BL/6 splenocytes
CD8-PE-Cy7 Validated by manufacturer
cKIT-APCe780 Validated by manufacturer against mouse bone marrow cells
CXCR4-FITC Validated by manufacturer against BALB/c thymocytes
EPCR Validated by manufacturer
Gr-1-BV605 Validated by manufacturer against C57BL/6 bone marrow cells
Gr-1-PerCP-Cy5.5 Validated by manufacturer against BALB/c bone-marrow leukocytes
Sca-1 -PECy7 Validated by manufacturer against C57BL/6 splenocytes
Sca1-PerCP-Cy5.5 Validated by manufacturer against C57BL/6 mouse lineage-negative bone marrow
Sca1-TotalSeqA Validated by manufacturer
Ter119-APC Validated by manufacturer against C57BL/6 bone marrow cells
Ter119-BV605 Validated by manufacturer against C57BL/6 bone marrow cells
Ter119-PECy7 Validated by manufacturer against C57BL/6 bone marrow cells

Animals and other research organisms

Policy information about studies involving animals; ARRIVE guidelines recommended for reporting animal research, and Sex and Gender in Research

Laboratory animals

C57B6/J (Jackson Laboratories, Stock #000664)
B6.SJL-Ptprca Pepcb/BoyJ (Jackson Laboratories, Stock #002014)
C57BL/6-Tg(UBC-GFP)30Scha/J (Jackson Laboratories, Stock #004353)
Only mice were used in our study.
Housing Conditions:
The lights throughout the St. Jude facility are controlled by the automated system, LiteWorks. The lights in animal rooms and

corridors are on an automated 12 hour on, 12 hour off light cycle. Other light cycles can be set if necessary for research objectives. During the off light cycle, the red lights can by used to access animal room. Each animal room has a separate thermostat and humidistat to control temperature and humidity at the room level. Temperature and humidity are continuously monitored and alarms alert personnel to excursions from defined temperature or humidity ranges. Animal care technicians record high and low temperatures and humidity daily on a room log sheet using an electronic digital thermometer/humidistat.

All mice were acquired from Jackson Laboratories (Bar Harbor, Maine) and housed in a pathogen-free facility. Every effort was made to ensure adult control and experimental mice (including recipients) were age and sex matched (6-16 weeks for cell collection; 8-10 weeks for transplantation recipients). Fetal and neonatal mice were age matched based on timed pregnancy. More information of animal models can be found in Supplementary Table 3

Wild animals

This study did not involve wild animals.

Reporting on sex

The findings presented her do not apply to one sex. In experimental design, every effort was made to ensure adult control and experimental mice (including recipients) were sex matched.

Field-collected samples

This study did not involve samples collected from the field.

Ethics oversight

All animal experiments were carried out according to procedures approved by the St. Jude Children's Research Hospital Institutional Animal Care and Use Committee and comply with all relevant ethical regulations regarding animal research. Protocol number: 531.

Note that full information on the approval of the study protocol must also be provided in the manuscript.

Flow Cytometry

Plots

Confirm that:

☒ The axis labels state the marker and fluorochrome used (e.g. CD4-FITC).

☒ The axis scales are clearly visible. Include numbers along axes only for bottom left plot of group (a 'group' is an analysis of identical markers).

☒ All plots are contour plots with outliers or pseudocolor plots.

☒ A numerical value for number of cells or percentage (with statistics) is provided.

Methodology

Sample preparation

To isolate hematopoietic cells in adult mice, tibias, femurs, pelvic bones, and spines were removed and bone marrow (BM) was released by crushing in ice cold PBS, followed by passage through 70µm filters. Cells were then resuspended in red blood cell lysis buffer for 5-10 minutes, washed with PBS/2% fetal calf serum (FCS), and resuspended in PBS/2% FCS. To isolate stomal cells in adult mice, crushed bone fragments were cut into small chips, combined with a portion of the released BM fraction, and placed in pre-warmed digestion media (PBS/2% FCS/0.4% Collagenase II/0.02% DNaseI) followed by gentle shaking (75RPM) at 37oC for 45min-1 hour. After digestion, cells were passed through a 70µm filter and lysed for 5-10 minutes, washed with PBS/2% FCS, and resuspended in PBS/2% FCS. Dissection of fetal tissues was performed under a dissection microscope in ice cold PBS. Briefly, fetal livers (FL) were removed and placed in ice cold PBS, followed by removal of all internal tissues and the skin. The fetal skeleton (except the bones of the head) was carefully removed and cleaned of any remaining tissues, washed in PBS, and placed in ice cold PBS for further processing. The isolation of hematopoietic and stromal cells from the fetal BM (FBM) followed the same protocol used for adult BM with the exclusion of the lysis step, as there was little trace of red blood cells. To isolate hematopoietic cells from FL, livers were gently crushed on a 70µm filter with the rubber end of a 1 mL syringe while passing ice cold PBS over the tissue. Following this isolation step, FL hematopoietic cells were lysed, washed with PBS/2% FCS, and resuspended in PBS/2% FCS.

Transplant recipients were periodically assessed for donor cell contribution to the PB. PB was collected from the retro-orbital plexus in heparinized capillary tubes, followed by lysis in red blood cell lysis buffer. Cells were then resuspended in PBS/2% FCS and analyzed by flow cytometry for donor and lineage contribution on a BD LSR Fortessa. For WBM primary and secondary transplants and separate bone transplants, PB was stained with CD45.1-FITC, CD45.2-v500, [B220, CD11b, Gr-1]-PerCP-Cy5.5, and [B220, CD4, CD8]-PE-Cy7. The myeloid lineage was considered CD11b+Gr-1+, the B cell lineage was considered B220+, and the T cell lineage was considered CD4+CD8+. For MPP2 limiting cell transplants, we also included a separate analysis of non-lysed PB stained with CD41-PerCPe710 (platelets) and Ter119-PECy7 (erythrocytes).

At a terminal (>20 weeks) timepoint after transplantation, recipients of WBM were euthanized and hematopoietic cells were isolated from the BM. Cells were assessed via flow cytometry on a BD LSR Fortessa for the presence of donor-derived CMPs (Lineage-c-Kit+Sca-1-CD34+CD32/16lo), GMPs (Lineage-c-Kit+Sca-1-CD34+CD32/16hi), MEPs (Lineage-c-Kit+Sca-1-CD34-CD32/16-), CLPs (Lineage-c-KitmidSca-1midCD127+), LT-HSCs, (Lineage-Sca-1+c-Kit+ (LSK)Flt3-CD48-CD150+), ST-HSCs (LSKFlt3-CD48-CD150-), MPP2s (LSKFlt3-CD48+CD150+), MPP3s (LSKFlt3-CD48+CD150-), and MPP4s (LSKFlt3+CD48+CD150-). In all cases, the Lineage- fraction showed low expression of CD4, CD8, B220, Gr-1, and Ter119.

Liver and entire skeleton BM hematopoietic cells were isolated from E15.5-postnatal day 0 (P0), P2, P4, P6, P8, P14, P21, and P28 CD45.2 mice and assessed via flow cytometry on a BD LSR Fortessa for the presence of LT-HSCs, ST-HSCs, MPP2s, MPP3s, and MPP4s.

Hematopoietic cells from E15.5-P0 livers were isolated and assessed via flow cytometry on a BD LSR Fortessa for the presence of LT-HSCs, ST-HSCs, MPP2s, MPP3s, and MPP4s, as well as cell-surface CXCR4.

For establishing mesenchymal stromal cell (MSC) cultures, hematopoietic cells were depleted using MojoSort anti-mouse CD45 beads, the remaining cells washed 3 times with PBS/2% FCS and cultured in αMEM + 10% FCS for 24 hours. After 24 hours, non-adherent cells were removed and the stroma monolayer was washed with PBS and cultured again with mesenchymal stroma cells MSC media. Cells were passaged 3 times and used for experiments.

Instrument

All cells were analyzed on a BD LSR Fortessa. For cell sorting, a BD Aria cell sorter was used.

Software

Flow cytometry data was collected using BDFACSDiva Software and analyzed with FlowJo (TreeStar).

Cell population abundance

Where possible, sorted cells were assessed for purity by running a portion of the collected sample through the same instrument used to sort the cells. For single cell and limiting cell transplant sorts, purity was not assessed.

Gating strategy

Gating strategies for all relevant cell populations can be found in the Supplementary Figures. Lymphocytes were first gated by forward scatter and side scatter, followed by viable cells via DAPI and single cells via forward and side scatter. For peripheral blood populations, cells were then gated based on CD45.2+ and GFP expression, followed by B lineage (B220+), myeloid lineage (Gr-1+CD11b+) or T lineage (CD4+CD8+). Unlysed peripheral blood was used to identify erythrocytes (Ter119+) platelets (CD41+). For bone marrow hematopoietic stem and progenitor cell populations, live singlets were gated on lineage low (B220/CD4/CD8/Gr-1/Ter119 neg), followed by c-Kit+ (LK cells). LK cells were further subdivided into GMP (CD34+CD32/16hi), CMP (CD34+CD32/16mid), or MEP (CD34-CD32/16-) cells. LK Sca1+ cells (LSK) were further subdivided into LT-HSC (CD135-CD48-CD150+), ST-HSC (CD135-CD48-CD150-), MPP2 (CD135-CD48+CD150+), MPP3 (CD135-CD48+CD150-) or MPP4 (CD135+CD48+CD150-) cells. Finally, CLPs were defined as Lineage(low)c-Kit(mid)Sca1(mid)CD127+ cells.

☒ Tick this box to confirm that a figure exemplifying the gating strategy is provided in the Supplementary Information.
