# Supplementary material for: Murine fetal bone marrow does not support functional hematopoietic stem and progenitor cells until birth
Source: Nat Commun. 2022 Sep 15;13:5403. doi: 10.1038/s41467-022-33092-4 (PMC9477881; doi:10.1038/s41467-022-33092-4)
Supplement: Supplementary file 2 — Peer Review File [file 41467_2022_33092_MOESM2_ESM.pdf]

Peer Review File

Murine fetal bone marrow does not support functional hematopoietic stem and progenitor cells until birth

Open Access This file is licensed under a Creative Commons Attribution 4.0 International License, which permits use, sharing, adaptation, distribution and reproduction in any medium or format, as long as you give appropriate credit to the original author(s) and the source, provide a link to the Creative Commons license, and indicate if changes were made. In the cases where the authors are anonymous, such as is the case for the reports of anonymous peer reviewers, author attribution should be to 'Anonymous Referee' followed by a clear attribution to the source work. The images or other third party material in this file are included in the article's Creative Commons license, unless indicated otherwise in a credit line to the material. If material is not included in the article's Creative Commons license and your intended use is not permitted by statutory regulation or exceeds the permitted use, you will need to obtain permission directly from the copyright holder. To view a copy of this license, visit http://creativecommons.org/licenses/by/4.0/.

Editorial Note: This manuscript has been previously reviewed at another journal that is not operating a transparent peer review scheme. This document only contains reviewer comments and rebuttal letters for versions considered at *Nature Communications* .

REVIEWERS' COMMENTS

Reviewer #1 (Remarks to the Author):

The authors have thoughtfully addressed the issues in my critique and the manuscript is substantially stronger than when first submitted. I think the data they provide will be an important contribution to the field.

Reviewer #2 (Remarks to the Author):

Authors has largely addressed my concerns.

REVIEWERS' COMMENTS

Reviewer #1 (Remarks to the Author):

The authors have thoughtfully addressed the issues in my critique and the manuscript is substantially stronger than when first submitted. I think the data they provide will be an important contribution to the field.

We appreciate the reviewer's comments and feel that they have strengthened the manuscript.

Reviewer #2 (Remarks to the Author):

Authors has largely addressed my concerns.

We appreciate the reviewer's comments and feel that they have strengthened the manuscript.
